# Pangenome analysis of *Paenibacillus polymyxa* strains reveals the existence of multiple and functionally distinct *Paenibacillus* species

**DOI:** 10.1128/aem.01740-24

**Published:** 2024-10-30

**Authors:** Federica Maggi, Anna Maria Giuliodori, Anna Brandi, Lucia Cimarelli, Roberto Alcántara, Stefano Pallotti, Consuelo Amantini, Dezemona Petrelli, Attilio Fabbretti, Roberto Spurio, Valerio Napolioni

**Affiliations:** 1 School of Biosciences and Veterinary Medicine, University of Camerino, Camerino, Italy; 2 Biomolecules Laboratory, Faculty of Health Sciences, Universidad Peruana de Ciencias Aplicadas, Lima, Peru; Colorado School of Mines, Golden, Colorado, USA

**Keywords:** evolutionary analysis, genomics, *Paenibacillus polymyxa*, pangenome

## Abstract

*Paenibacillus polymyxa*, a Gram-positive bacterium commonly found in soil and plant roots, plays an important role in the environment due to its nitrogen-fixing ability and is renowned for producing antibiotics like polymyxin. In this study, we present a robust framework for investigating the evolutionary and taxonomic connections of strains belonging to *P. polymyxa* available at the National Center for Biotechnology Information, as well as five new additional strains isolated at the University of Camerino (Italy), through pangenome analysis. These strains can produce secondary metabolites active against *Staphylococcus aureus* and *Klebsiella pneumoniae*. Employing techniques such as digital DNA-DNA hybridization (dDDH), average nucleotide identity (ANI) estimation, OrthoFinder, and ribosomal multilocus sequence typing, we consistently divided these *P. polymyxa* strains into four clusters, which differ significantly in terms of ANI and dDDH percentages, both considered as reference indices for separating bacterial species. Moreover, the strains of Cluster 2 were re-classified as belonging to the *Paenibacillus ottowii* species. By comparing the pangenomes, we identified the core genes of each cluster and analyzed them to recognize distinctive features in terms of biosynthetic/metabolic potential. The comparison of pangenomes also allowed us to pinpoint differences between clusters in terms of genetic variability and the percentage of the genome dedicated to core and accessory genes. In conclusion, the data obtained from our analyses of strains belonging to the *P. polymyxa* species converge toward a necessary reclassification, which will require a fundamental contribution from microbiologists in the near future.

**IMPORTANCE:**

The development of sequencing technologies has led to an exponential increase in microbial sequencing data. Accurately identifying bacterial species remains a challenge because of extensive intra-species variability, the need for multiple identification methods, and the rapid rate of taxonomic changes. A substantial contribution to elucidating the relationships among related bacterial strains comes from comparing their genomic sequences. This comparison also allows for the identification of the “pangenome,” which is the set of genes shared by all individuals of a species, as well as the set of genes that are unique to subpopulations. Here, we applied this approach to *Paenibacillus polymyxa*, a species studied for its potential as a biofertilizer and biocontrol agent and known as an antibiotic producer. Our work highlights the need for a more efficient classification of this bacterial species and provides a better delineation of strains with different properties.

## INTRODUCTION

*Paenibacillus polymyxa* is a Gram-positive, rod-shaped bacterium commonly found in soil and plant roots. This bacterium is known for its nitrogen-fixing capacity, which makes it an important microorganism in the agri-environmental sector, as it can improve crop growth and yield. In addition, *P. polymyxa* is useful in industrial and biotechnological sectors, including bioremediation, food chain applications, biofuels production, being a source of enzymes that can break down complex organic compounds such as hydrocarbons and pesticides, or complex sugars, such as amylases, as well as an excellent producer of bioactive compounds, like antimicrobial agents (1).

Pangenome analysis provides a more complete understanding of genomic diversity within a species. The term “pangenome” refers to the entire set of genes shared by all individuals of a particular species, as well as the set of genes that are unique to certain individuals or subpopulations (2, 3). Traditionally, the focus of genome sequencing and analysis has been on the reference genome, i.e., a single representative genome that is used as a basis for comparison with other genomes of the same species. However, it is now widely recognized that a single reference genome cannot capture the entire genomic diversity of a species (4). Pangenome analysis allows researchers to identify the core set of genes, and by inference, the common metabolic capabilities shared by all individuals of a species, as well as variable genes that are unique to certain individuals or populations. This information can be used to better understand the genetic basis of traits and diseases, as well as to develop more effective strategies for crop selection and genetic engineering. Furthermore, pangenome analysis of a target species, by defining the fraction of core genes common to all members, can provide information on the evolutionary history and population dynamics of a species, as well as its adaptation to different environments (2, 3).

To date, only a few pangenome studies on *P. polymyxa* are available, and they are focused on the aspects related to the environmental and agricultural areas (5, 6). Here, we present a robust framework for examining evolutionary and taxonomical relationships by comparing genomes of strains belonging to the species available at the National Center for Biotechnology Information (NCBI), as well as new strains available at the Culture Collection of Microorganisms of the University of Camerino (Camerino, Italy). Based on different phylogenomic metrics, we found *P. polymyxa* strains to be consistently divided into four clusters, which we propose to be different species, with an open pangenome. Given the high dynamism of the genomes belonging to this species, this comparative analysis provides new insight into the genomic content and variability of *P. polymyxa*. The analysis of genes belonging to the core genome can help in distinguishing strains with different properties, opening the possibility not only of a more robust and accurate classification of this biotechnology relevant bacteria but also to uncover relevant mechanisms, which would not be detected by standard microbiological techniques.

## RESULTS

### Isolation and characterization of five new *P. polymyxa* strains

**Fig 1:**
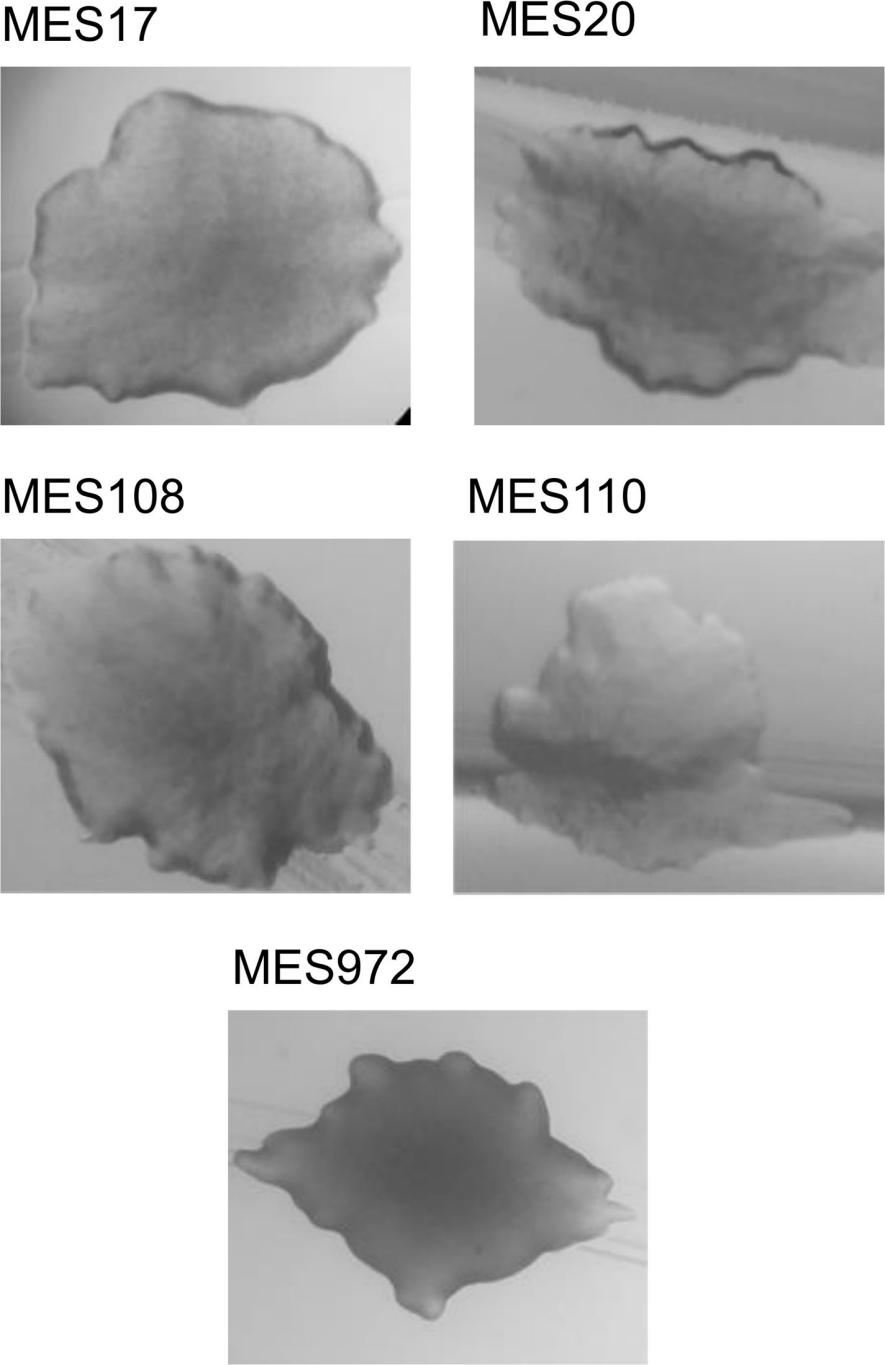
Morphological features of the MES strain colonies. Microscopic images of the colonies of the indicated MES strains grown for 4 days at 15°C on LB plates.

The newly isolated strains MES17, 20, 108, 110, and 972 were identified as Gram-positive, facultative anaerobic bacteria. After 2 days of incubation at 15°C on LB medium, colonies were white and circular with a diameter of 1–2 mm. When observed at the stereomicroscope, all colonies showed raised elevation and irregular margins and were morphologically very similar (Fig. 1).

All strains were able to grow at both 15°C and 30°C on LB, TSA, or R2A plates (File S1; Fig. S1) although MES972 growth was initially slower compared to the other strains, especially at 15°C.

**Fig 2:**
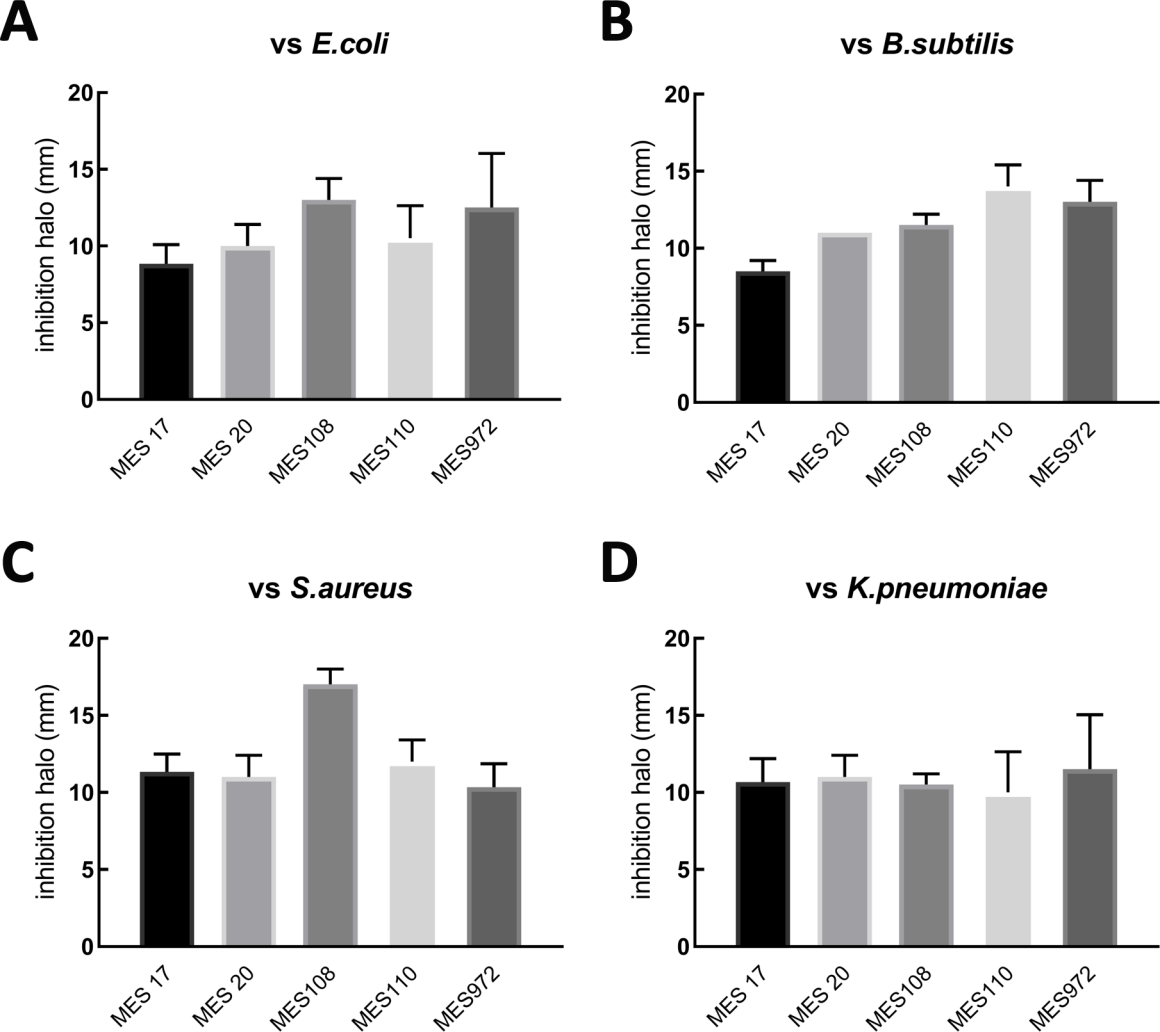
Growth inhibition induced by secondary metabolites released by MES strains on solid medium. Inhibition of *E. coli* ATCC 25922 (**A**), *B. subtilis* ATCC 6633 (**B**), *S. aureus* ATCC 25923 (**C**), and *K. pneumoniae* ATCC13883 (**D**) growth produced by the indicated MES strains grown as a spot of 20 mm at the center of each LB plate. After 24 hours of incubation at 30°C, the zone of growth inhibition was measured in terms of millimeters from the outer edge of the circular area containing the MES strain under examination. The inhibition halo is the average distance between the outer edge of the MES spot and the boundary line of growth of each tester strain. Error bars indicate the standard deviation calculated from triplicate measurements.

Based on the carbon metabolism profile assessed by API50CH test (Table 1), all MES strains were identified as *P. polymyxa*. Among the strains, MES972 was the only one capable of metabolizing L-rhamnose and D-melezitose, while MES108 could not convert methyl-beta-D-xylopyranoside, D-turanose, and potassium gluconate. In addition, all MES strains tested positive for starch hydrolysis and acid production from D-trehalose but tested negative for indole production and citrate utilization (Table 2), a pattern typical of *P. polymyxa* (7). We also performed a growth inhibition assay to assess the production of antimicrobial compounds by the MES strains. As shown in Fig. 2, all five MES strains were able to inhibit the growth, with a comparable activity on four tester strains, namely *Escherichia coli* ATCC 25922, *Staphylococcus aureus* ATCC 25923, *Klebsiella pneumoniae* ATCC 13883, and *Bacillus subtilis* ATCC 6633.

**TABLE 1:** API 50 CH results^*c*^

Substrates	Strains						
	MES17	MES20	MES108	MES110	MES972	*Paenibacillus ottowi* ^*a*^	*P. polymyxa* ATCC 842^*b*^
Glycerol	+	+	+	+	+	+	+
L-arabinose	+	+	+	+	+	+	+
D-ribose	+	+	+	+	+	+	+
D-xylose	+	+	+	+	+	+	+
Methyl-beta-D-xylopyranoside	+	-	-	+	+	+	+
D-galactose	+	+	+	+	+	+	+
D-glucose	+	+	+	+	+	+	+
D-fructose	+	+	+	+	+	+	+
D-mannose	+	+	+	+	+	+	+
L-rhamnose	−	−	−	−	+	−	+/−
D-mannitol	+	+	+	+	+	+	+
Methyl-alpha-D-glucopyranoside	+	+	+	+	+	+	+
Amygdalin	+	+	+	+	+	+	+
Arbutin	+	+	+	+	+	+	+
Esculin ferric citrate	+	+	+	+	+	+	+
Salicin	+	+	+	+	+	+	+
D-cellobiose	+	+	+	+	+	+	+
D-maltose	+	+	+	+	+	+	+
D-lactose	+	+	+	+	+	+	+
D-melibiose	+	+	+	+	+	+	+
D-saccharose (sucrose)	+	+	+	+	+	+	+
D-trehalose	+	+	+	+	+	+	+
Inulin	+	+	+	+	+	−	+/−
D-melezitose	−	−	−	−	+	−	+/−
D-raffinose	+	+	+	+	+	+	+
Amidon (starch)	+	+	+	+	+	+	+
Glycogen	+	+	+	+	+	+	+
Gentiobiose	+	+	+	+	+	+	+
D-turanose	+	+	−	+	+	+	+
Potassium gluconate	+	+	−	+	+	−	+

^*a*^ Reference (8).

^*b*^ https://bacdive.dsmz.de/strain/11487.

^*c*^ −, negative reaction; +, positive reaction.

**TABLE 2:** Biochemical tests^*c*^

	MES17	MES20	MES108	MES110	MES972	*Paenibacillus ottowi* ^*a*^	*P. polymyxa* ATCC 842^*b*^
Isolation source	Soil	Soil	Transitional water	Transitional water	Oak roots	Anaerobic digester processing bovine manure	Soil
Growth at 16°C	++	++	++	++	+	++	n.t.
Growth at 30°C	+++	+++	+++	+++	++	+++	+++
Starch hydrolysis	+	+	+	+	+	+	+
Indole production	−	−	−	−	−	n.t.	−
Acid production from trehalose	+	+	+	+	+	+	+
Citrate utilization	−	−	−	−	−	−	−

^*a*^ Reference (8).

^*b*^ https://bacdive.dsmz.de/strain/11487.

^*c*^ −, negative reaction; +, positive reaction/growth entity; n.t., not tested. For starch hydrolysis, *E. coli* ATCC 25922 and *B. subtilis* ATCC 6633 were used as negative and positive controls, respectively. For indole production, *K. pneumoniae* ATCC13883 and *E. coli* ATCC 25922 were used as negative and positive controls, respectively. For trehalose assay, *E. coli* ATCC 25922 was used as a positive control. For citrate utilization test, *K. pneumoniae* ATCC13883 and *E. coli* ATCC 25922 were used as positive and negative controls, respectively.

Genome sequencing of the MES strains generated the results and assembly statistics shown in File S1 and Table S1. The chromosome size of these strains spans from ~5.66 to ~6.08 Mb, with an average GC content between 45.1% and 45.6%. The annotated protein-coding genes were between 5,120 (MES108) and 5,536 (MES972), while tRNA genes ranged from 83 (MES108) to 93 (MES 17 and MES20).

### Selection of *P. polymyxa* strains for the pangenome analysis

Pangenome analyses can be misinterpreted if confounding, mis-assembled, and duplicate genome strains are included (9). Thus, we performed an extensive quality control (QC) of all the genomes considered, using either evidence from the literature or by using several bioinformatic tools, such as Benchmarking Universal Single-Copy Orthologs (BUSCO) (10), OrthoFinder (11), FastANI (12), autoMLST (13), and Type (Strain) Genome Server (TYGS) (14). The excluded strains are reported in File S4, along with the reasons for exclusion. To summarize, we removed 27 of the 108 available assemblies in the NCBI portal (accessed in February 2023) due to abnormalities (i.e., contaminations or lack of RefSeq), while seven strains were excluded because they were considered duplicates, and three strains were removed due to a low level of completeness according to BUSCO (10). The combination of analyses carried out with OrthoFinder (11) (File S1; Fig. S2) and FastANI (12) (File S1; Fig. S3) indicated that one strain (ISL-58) clustered separately from the rest of the data. Thus, it was repositioned into the correct species using autoMLST (13), displaying the highest identity with *Paenibacillus amylolyticus* (ANI = 94.9%). Then, we used TYGS (14) for genome-based taxonomic classification according to digital DNA-DNA hybridization (dDDH) and FastANI (12) to compute ANI values. Based on the “identification” table results in TYGS, strains ND24 and ZF129 were identified as *Paenibacillus ottowii* (File S1; Fig. S4A). Therefore, the *P. ottowii* type strain MS2379 (8) was included in the analysis, and OrthoFinder (11) was applied to determine its proximity to our data set, as shown in Fig. S4B. Finally, we also excluded 14 duplicated genomes according to ANI and dDDH values; a percentage greater than or equal to 99% of ANI and dDDH was set as the threshold for species duplication in groups with more than three strains. After this final QC, the number of unique genome strains undergoing pangenome analysis was 62. The final complete matrices of ANI and dDDH values are available as Files S2 and S3.

### Pangenome analysis of *P. polymyxa*

Compared to previous studies reporting the pangenome analysis of *P. polymyxa* strains (5, 6), our study extends the investigation to 36 and 52 additional strains publicly available in NCBI, respectively, along with the five genomes sequenced *ex novo* at the University of Camerino and the genome of *P. ottowii* type strain MS2379 (File S4).

**Fig 3:**
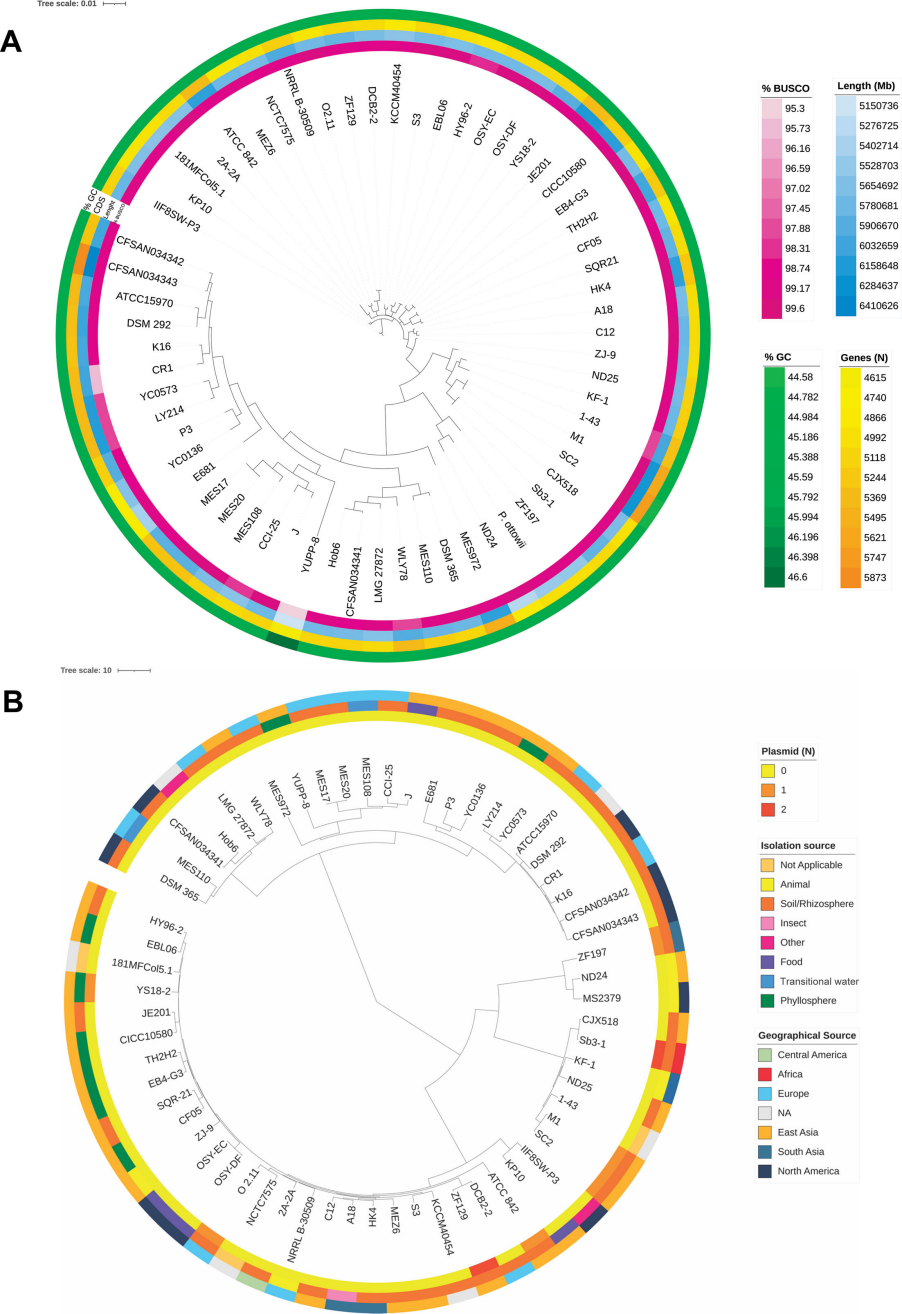
Phylogenetic tree features of the considered 62 strains obtained with OrthoFinder and FastANI. The circular heatmaps, from inside to outside, show (A) the percentage of BUSCO, genome size, number of genes, and GC content for each strain, (B) the number of plasmids, the isolation source, and the geographical origin of each strain.

The average size of the genomes and the GC content of the 62 strains are 5.82 Mb and 45.4%, respectively, with an average BUSCO percentage of 98.9 (Fig. 3A). Of the strains analyzed, nine have complete genomes, while 53 are classified as draft. Moreover, the average non-chromosomal genome size is 25.7 Kb, and 8 of the 62 strains considered (13%) have one or two plasmids (Fig. 3B).

**Fig 4:**
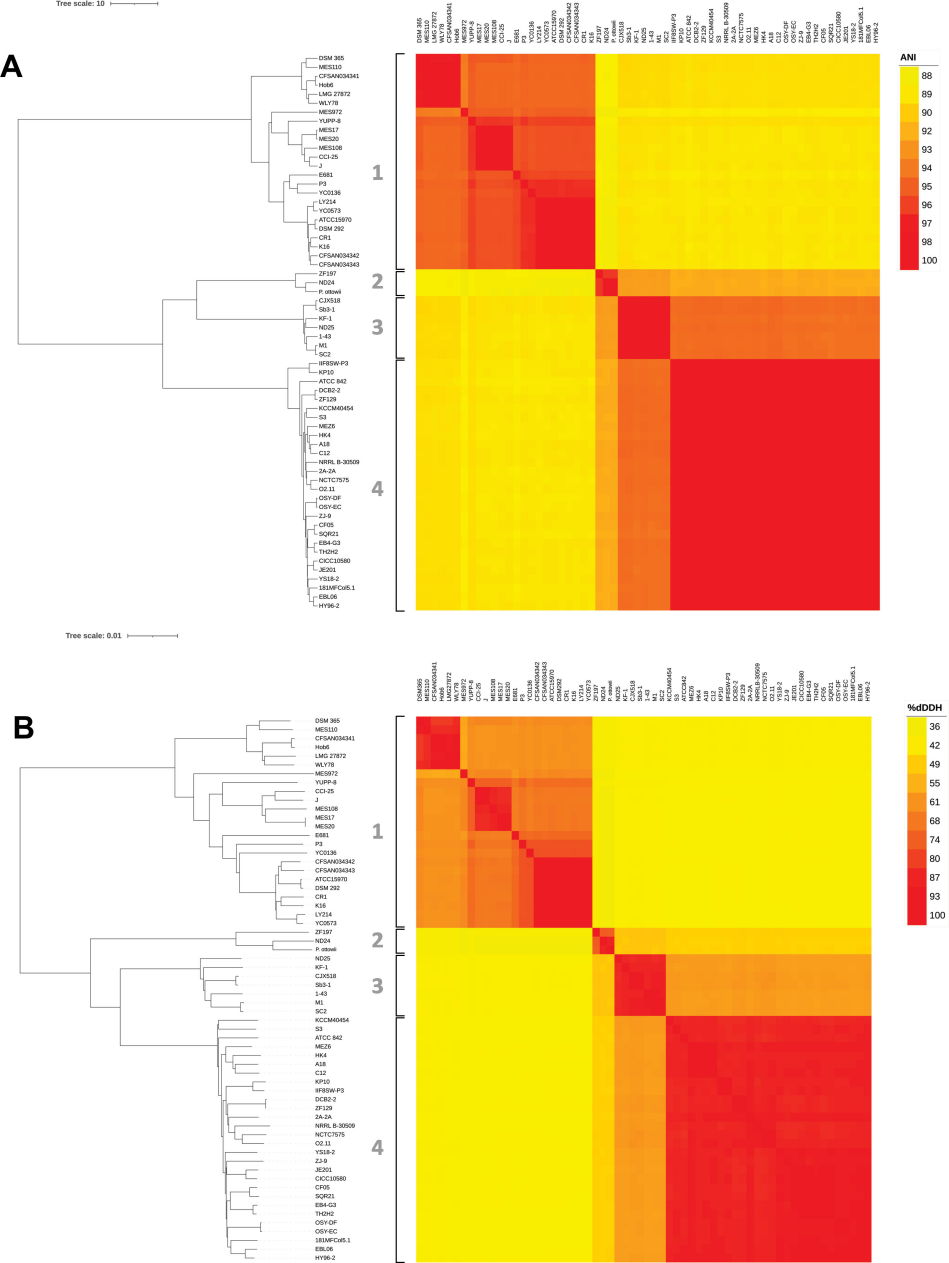
Correlation matrix of the average nucleotide identity (**A**) and the digital DNA-DNA hybridization (**B**) values of the analyzed genomes. The ANI percentage varies between 88% (yellow) and 100% (red), while dDDh varies between 35% (yellow) and 100% (red).

Using ANI and dDDH, species can be accurately delineated and genetic relatedness can be assessed (15). In agreement with the 70% dDDH (16) and 95% ANI (17) thresholds for species delineation, our data set was further divided into four main clusters (Fig. 4): Cluster 1, composed of 24 strains, Cluster 2, composed of 3 strains, including *P. ottowii*, Cluster 3, composed of 7 strains, and Cluster 4, composed of 28 strains.

**Fig 5:**
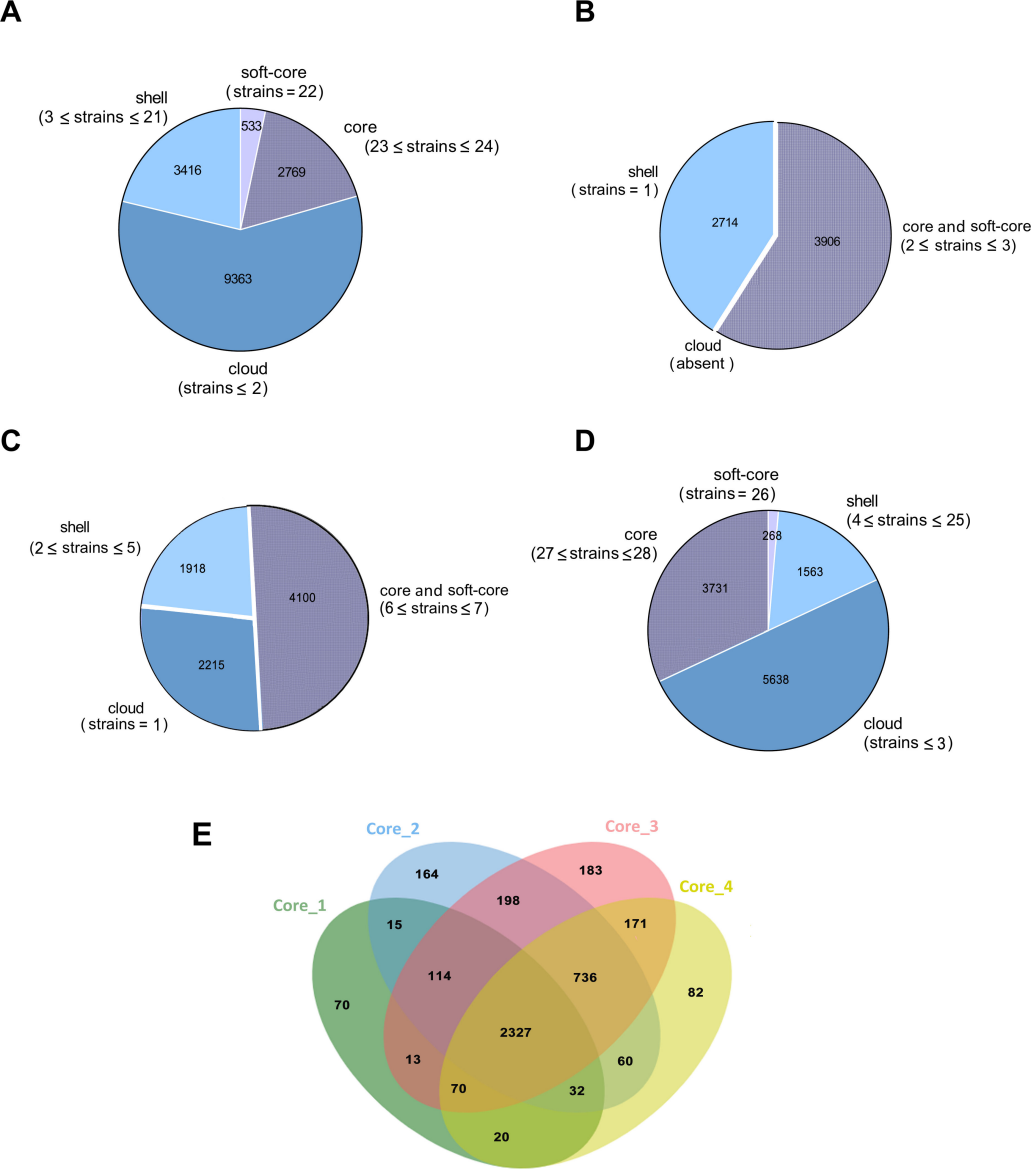
Pangenome of *P. polymyxa*. Representation of the number of genes belonging to the core, soft core, shell, or cloud of the *P. polymyxa* strains by pie chart belonging to Cluster 1 (**A**), Cluster 2 (**B**), Cluster 3 (**C**), and Cluster 4 (**D**). The numbers in parentheses refer to the criteria used to separate the genes into core, soft core, shell, or cloud genomes based on their presence in the strains, as described in Materials and Methods. (**E**) Graphical representation of the number of core genes shared between the four pangenomes using the Jvenn tool (19).

The pangenome analysis of the four clusters, performed using Roary (18), shows that in Cluster 1 there are 2,769 genes linked to the core genome, 533 genes in the soft core, 3,416 linked to the shell genome, and 9,363 cloud genes (Fig. 5A). In Cluster 2, 3,906 genes are linked to the core genome and 2,714 to the shell (Fig. 5B), while in Cluster 3, 4,100 genes are within the core genome, 1,918 to the shell genome, and 2,215 to the cloud genes (Fig. 5C). Finally, in Cluster 4, 3,731 genes fall in the core genome, 268 genes in the soft core, 1,563 in the shell genome, and 5,638 are cloud genes (Fig. 5D). Clusters 1 (*α* = −0.90 ± 0.28), 2 (*α* = −0.53 ± 0.29), 3 (*α* = −0.17 ± 0.03), and 4 (*α* = −0.94 ± 0.38) all show an open pangenome, as indicated by *α* values < 1. This is a typical genomic feature of bacterial species that can easily exchange genes and genetic elements and are able to colonize and thrive in disparate environments.

Cluster 1 allocates 17.4% of its genome to core genes, while Cluster 4 dedicates 33.3% of its genome to them, with this difference being statistically significant (*P* < 0.001). Hence, Cluster 1 is composed of a more heterogeneous group of bacteria that share a narrower set of core genes and enrich the genome with accessory genes, while Cluster 4 consists of overall more homogeneous strains. As highlighted in the Venn diagram (Fig. 5E), the common genes among the core genomes of the four clusters are 2,327, while the genes distinctive of each core genome, which we have named type-core genes, are 70 for Cluster 1, 164 for Cluster 2, 183 for Cluster 3, and 82 for Cluster 4. Note that type-core genes are common to all strains belonging to the same cluster and are not present in the core genome of the other clusters. However, in the latter, they may be present as accessory genes. The list of the type-core genes of the four clusters, as well as the core genes and the common genes, is shown in File S5.

Phylogenetic trees (File S1; Fig. S5 to S7) were constructed using both Roary (18) and Parsnp (20). The latter reconstructs phylogenetic relationships based on nucleotide variants found by comparing only the core genes, which are presumably inherited vertically. Both the Roary and Parsnp analyses showed that Cluster 1 strains (File S1; Fig. S5) display greater genetic variability compared to those belonging to Cluster 4 (File S1; Fig. S7), which are more strictly related with one another.

### Ribosomal gene locus typing for rapid identification of the clusters

**Fig 6:**
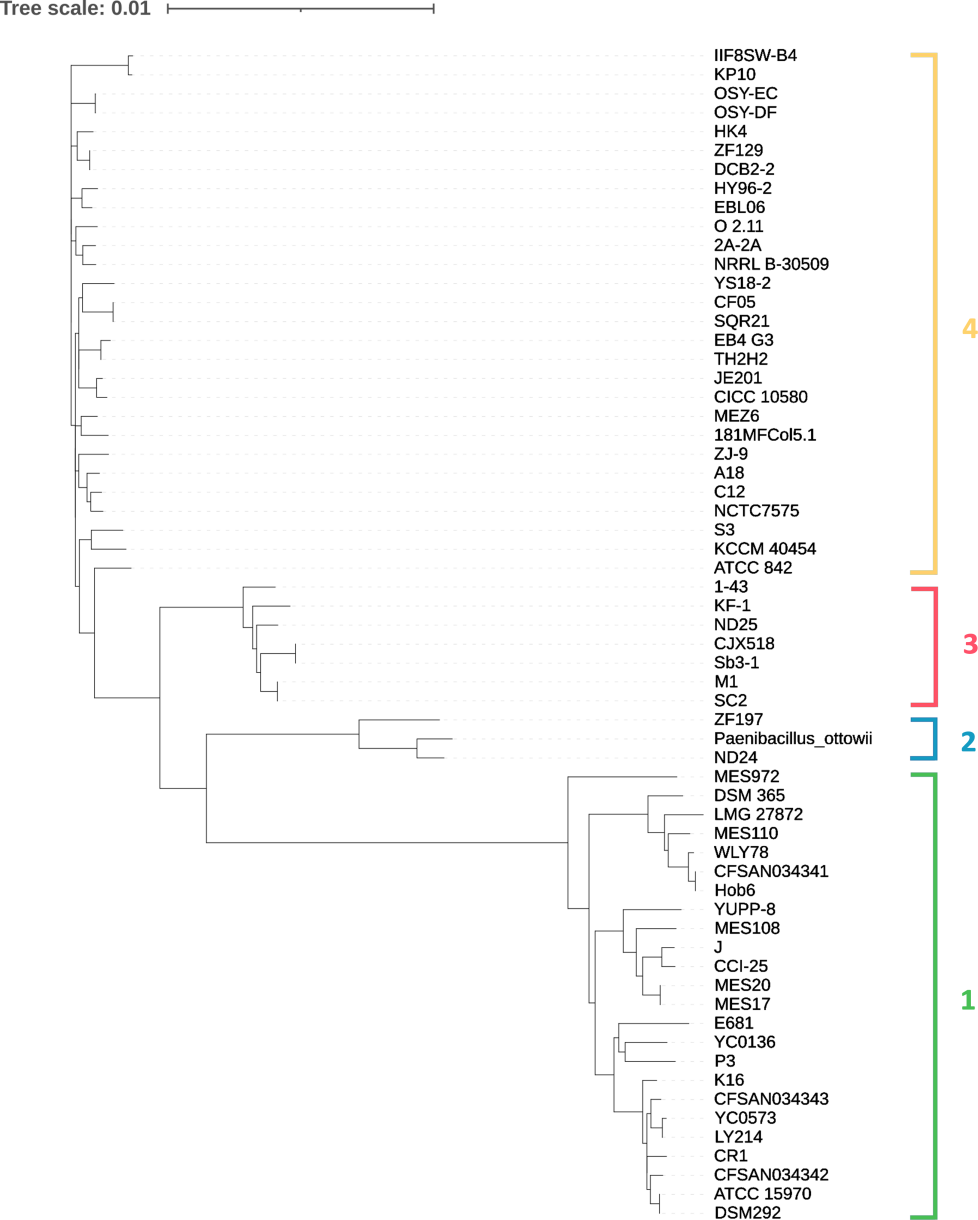
Phylogenetic tree obtained by rMLST investigation. The four clusters previously identified by FastANI and dDDh studies are indicated with the corresponding numbers and colors used in Fig. 5E for the Venn diagram.

The metabolic profiles of the type strain *P. polymyxa* ATCC 842 (Cluster 4), the MES strains we isolated (Cluster 1), and *P. ottowii* (Cluster 2) are extremely similar, as can be seen from Tables 1 and 2. Therefore, such a method does not seem useful for discriminating these different groups. Recently, it has been demonstrated that strains ATCC 842T, DSM 292, and DSM 365 can be distinguished by comparing mass fingerprint profiles obtained through MALDI-TOF analysis (21). The mass fingerprint profile is mainly characterized by ribosomal proteins, which are abundant and subjected to stabilizing selection for functional conservation, making them very useful for establishing phylogenetic relationships. Thus, we applied ribosomal multilocus sequence typing (rMLST) analysis (22) to the *P. polymyxa* strains to verify if the variations present in the *rps* loci were useful for detecting the four groups identified using ANI and dDDH. The result of the analysis conducted using the rMLST database hosted by the PubMLST.org website (23), and shown in Fig. 6, demonstrates that rMLST resolves the *P. polymyxa* strains into the same four clusters. Therefore, *rps* genes can be used as a typing tool for *P. polymyxa* identification. As examples of sequences useful for performing this analysis, Fig. S8 to S12 (File S1) show the multiple alignments of the L13, L14, and L35 ribosomal genes and proteins. From these, it is evident that the single nucleotide variants present in the central region of the L13 gene allow discrimination of the four different clusters, while the sequences of L14 and L35 allow the separation of Clusters 1 and 2 but not of Cluster 3 from 4. The comparison between the protein sequences of these proteins reduces the discriminatory capacity of the analysis to the point that, in the case of L14, no differences are observed between the various sequences.

### *P. polymyxa* clustering groups differ in their functional metabolic potential

**Fig 7:**
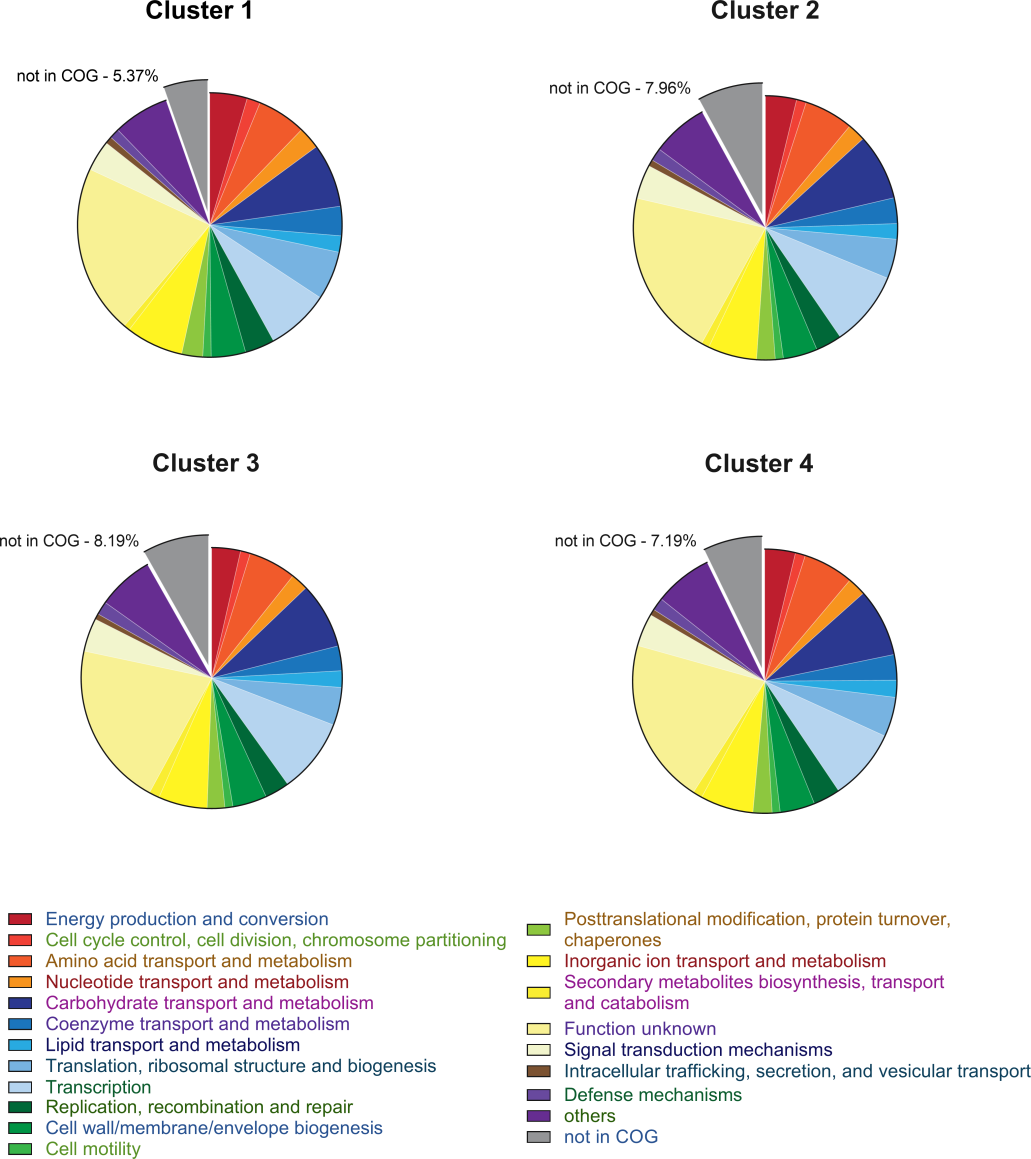
Differences in the number of COG categories identified by EggNOG-mapper annotation in the core genomes of the four clusters. The pie charts depict the distribution of various COG categories indicated in the legend and represented by the corresponding colors. The percentage of each category was calculated based on the number of COG categories assigned to the identified genes (see File S5 and Materials and Methods).

The pangenome analysis separated the strains into four well-defined clusters from a phylogenetic perspective. To investigate the possible functional and metabolic differences of the four core genomes, these were annotated with EggNOG-mapper (24), and subsequently, the COG (Clusters of Orthologous Genes) categories assigned to the identified genes were examined (25, 26). This analysis highlighted a statistically significant difference (*P*-value < 0.0001) only in the genes not associated with any COG category (not in COG), which are less abundant in Cluster 1 (Fig. 7). Furthermore, when we compared the COG categories present in the group of type-core genes with the core genes of each respective cluster, we identified other statistically significant differences (see File S5). In the type-core of Cluster 1, the aforementioned reduction of genes not assigned to any COG is confirmed, along with a simultaneous enrichment of the same COG category in type-core of Clusters 2 and 3. Meanwhile, in the latter type-cores, there is a reduction of genes belonging to category P (inorganic ion transport and metabolism). These differences are statistically significant both when comparing the type-cores with each other and when comparing type-core vs core genes. Finally, type-cores of Clusters 1 and 4 are enriched in genes involved in secondary metabolite biosynthesis compared to their corresponding core genomes (File S5).

**Fig 8:**
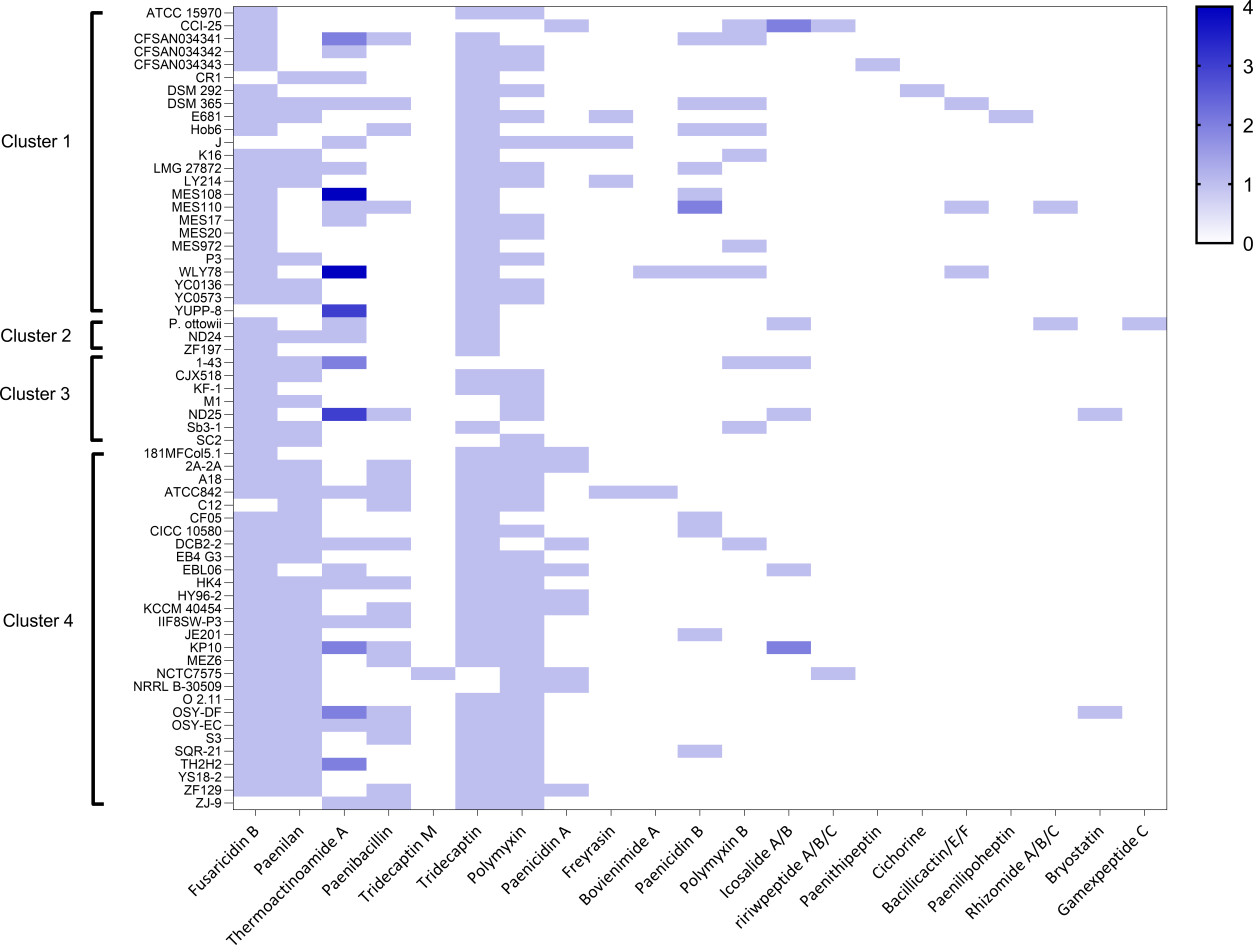
*In silico* prediction of secondary metabolites using antiSMASH. Only the secondary metabolites produced by BGC with a similarity of >80% with known BGC of the Minimum Information about a Biosynthetic Gene Cluster repository were selected and reported. For each strain, the heatmap shows the presence (from 1 to 4) or absence (= 0) of the corresponding BGCs involved in the synthesis of the indicated compound. Heatmap was created using GraphPad version 9.3.

Furthermore, to gain relevant insights into the differential biosynthetic potential of the four clusters, we employed antiSMASH version 7.0 (27). This analysis shows that Cluster 4 harbors a greater number of genes involved in the production of paenilan (*P* = 0.0002) and paenibacillin (*P* = 0.012) (Table 3; Fig. 8). We also observed a difference in the percentage of strains identified as producers of polymyxin A and B in the clusters (Table 3). Although this difference is statistically significant, the high degree of similarity between the biosynthetic gene clusters (BGCs) producing these two highly similar molecules makes the assignment uncertain and requires further confirmation.

**TABLE 3:** AntiSMASH prediction of the secondary metabolites^*a*^

Secondary metabolites	Cluster 1		Cluster 2		Cluster 3		Cluster 4		
	#BGC/strains	%	#BGC/ strains	%	#BGC/strains	%	#BGC/strains	%	*P* value
Fusaricidin B	21/24	87.50	3/3	100.00	6/6	100.00	27/29	93.10	ns
Paenilan	9/24	37.50	1/3	33.33	5/6	83.33	25/29	86.21	0.0004
Thermoactinoamide A	20/24	83.33	2/3	66.67	2/6	33.33	16/29	55.17	0.04
Paenibacillin	4/24	16.67	0/3	0.00	1/6	16.67	15/29	51.72	0.01
Tridecaptin M	0/24	0.00	0/3	0.00	0/6	0.00	1/29	3.45	ns
Tridecaptin	23/24	95.83	3/3	100.00	3/6	50.00	26/29	89.66	ns
Polymyxin	13/24	54.17	0/3	0.00	4/6	66.67	27/29	93.10	0.0014
Paenicidin A	2/24	8.33	0/3	0.00	1/6	16.67	8/29	27.59	ns
Freyrasin	3/24	12.50	0/3	0.00	0/6	0.00	1/29	3.45	ns
Bovienimide A	1/24	4.17	0/3	0.00	0/6	0.00	1/29	3.45	ns
Paenicidin B	8/24	33.33	0/3	0.00	0/6	0.00	4/29	13.79	ns
Polymyxin B	7/24	29.17	0/3	0.00	2/6	33.33	1/29	3.45	0.017
Icosalide A/B	2/24	8.33	1/3	33.33	1/6	16.67	4/29	13.79	ns
Ririwpeptide A/B/C	1/24	4.17	0/3	0.00	0/6	0.00	1/29	3.45	ns
Paenithipeptin	1/24	4.17	0/3	0.00	0/6	0.00	0/29	0.00	ns
Cichorine	1/24	4.17	0/3	0.00	0/6	0.00	0/29	0.00	ns
Bacillicactin/E/F	3/24	12.50	0/3	0.00	0/6	0.00	0/29	0.00	ns
Paenilipoheptin	1/24	4.17	0/3	0.00	0/6	0.00	0/29	0.00	ns
Rhizomide A/B/C	1/24	4.17	1/3	33.33	0/6	0.00	0/29	0.00	ns
Bryostatin	0/24	0.00	0/3	0.00	0/6	0.00	2/29	6.90	ns
Gamexpeptide C	0/24	0.00	1/3	33.33	0/6	0.00	0/29	0.00	0.0002

^*a*^ The *P*-value was calculated using Fisher’s exact test. ns, not significant (*P* > 0.05).

### Screening of genes for antibiotic resistance using CARD tool

The core genomes of the four clusters were analyzed with CARD’s (Comprehensive Antibiotic Resistance Database) Resistance Gene Identifier (RGI main) tool (28) to assess the presence of genes involved in antibiotic resistance. At the core genome level, all clusters have antibiotic resistance genes with “Strict” or “Perfect” search criteria. All clusters carry genes for antibiotic resistance belonging to the LlmA 23S ribosomal RNA methyltransferase family (identity > 80%) involved in resistance to the class of antibiotics known as lincosamides, while Clusters 3 and 4 carry the *fosBx1* and *qacG* genes, which confer resistance against phosphonic acid antibiotics and disinfectant and antiseptic agents, respectively (Table 4).

**TABLE 4:** Detection of antibiotic resistance genes by CARD**^*a*^**

Cluster	ARO term	AMR gene family	Drug class	Resistance mechanism	% Identity	% Length
1	*LimA 23S* ribosomal RNA methyltrasferase	Llm 23S ribosomal RNA methyltransferase	Glycoprotein antibiotic	Antibiotic target alteration	85.0	100.00
2	*LlmA 23S* ribosomal RNA methyltransferase	Llm 23S ribosomal RNA methyltransferase	Lincosamide antibiotic	Antibiotic target alteration	85.0	100.00
3	*FosBx1*	Fosfomycin thiol transferase	Phosphonic acid antibiotic	Antibiotic inactivation	65.0	101.45
	*qacG*	Small multidrug resistance antibiotic efflux pump	Disinfecting agents and antiseptics	Antibiotic efflux	61.7	114.95
	*LlmA 23S* ribosomal RNA methyltransferase	Llm 23S ribosomal RNA methyltransferase	Lincosamide antibiotic	Antibiotic target alteration	85.0	100.00
4	*FosBx1*	Fosfomycin thiol transferase	Phosphonic acid antibiotic	Antibiotic inactivation	64.2	101.45
	*qacG*	Small multidrug resistance antibiotic efflux pump	Disinfecting agents and antiseptics	Antibiotic efflux	61.7	114.95
	*LlmA* 23S ribosomal RNA methyltransferase	Llm 23S ribosomal RNA methyltransferase	Lincosamide antibiotic	Antibiotic target alteration	85.0	100.00

^*a*^ The identity of region matches, and the length of reference sequences is reported as percentage. The analysis was carried out with the Strict RGI and the protein homolog model criteria.

### Plasmid analysis

Based on their size, plasmids can be classified (29) into megaplasmids (size > 350 kb, pPPM1a and pSC2 present in strains M1 and SC2, respectively) and plasmids (size < 350 kb, pSb31l, pSb31s, pPpO211, pPo45, pATCC842, pYS18-2, pAP1, and pAP2 of strains Sb-3, O2.11, 2020, ATCC 842, yS18-2, and ZF129). Among the plasmids, pSb31l and pPpO211 have the largest sizes, while pSb31s has the smallest (Table 5). The GC content of these replicons is generally lower than that of the chromosomes, as expected (29), being approximately 38% for the two megaplasmids and around 42% for the plasmids (Table 5). Exceptions are pPpO211 and pSb31s, which have a GC% comparable to that of the chromosome.

**TABLE 5:** Plasmid features^*a*^

	Replicons									
	pPPM1a	pSC2	pSb31l	pSb31s	pPO211	pPo45	pATCC842	pYS18-2	pAP1	pAP2
Strain	M1	SC2	Sb3-1	Sb31	O 2.11	2020	ATCC842	YS18-2	ZF29	ZF29
Size (bp)	366,576	510,118	223,537	8,109	248,380	45,521	45,524	43,724	79,020	37,602
GC content (%)	38	38	42	45	45	42	42	42	42	42
Replication relaxation motif or Rep protein	+	+	+	+	−	−	−	−	−	−
ParM/StbA or ParM	+	+	−	−	−	+	+	−	+	+
Conjugation-related protein(s)	−	+	+	−	−	+	+	+	−	+
tRNA, rRNA, protein synthesis-related genes	+	+	+	−	+	−	−	−	−	−

^*a*^ +, present; −, absent.

Both pPPM1a and pSC2 megaplasmids, and pSb31l plasmid, contain genes with a motif identified as “Relaxation and Replication.” Additionally, pPPM1a and pSC2, along with pPo45, pATCC842, pAP1, and pAP2, contain genes annotated as encoding ParM/StbA family proteins, presumably involved in replicon segregation. pSb31s carries an annotated Rep gene, while no gene with a domain/motif related to plasmid replication has been identified in pPpO211. Finally, pSC2, pSb31l, pPo45, pATCC842, pYS18-2, and pAP2 include at least one gene annotated as involved in conjugation.

A BLAST analysis revealed that homologs of the Relaxation and Replication and the ParM/StbA proteins of pPPM1a are present in strain 1-43, which therefore might potentially harbor a megaplasmid. Orthologs of the “Relaxation and Replication” and conjugation-related proteins from pSb31l are present in strains H4K, CICC 10580, NRRLB 30509, and TH2H2. The ParM/StbA proteins in pPo45 and pATCC842 are identical and share 95% identity with the one of pAP2, while they show no homology with the proteins present in the other plasmids/megaplasmids. Also, the conjugal transfer proteins of these plasmids are highly related to one another, and their homologs are present in strain hob6.

## DISCUSSION

Recent advances in modern molecular techniques of genomics and proteomics offer stimulating alternatives to conventional procedures for the characterization and identification of microorganisms. Through pangenome analyses, it is possible to gain an overview of the genomic diversity of an organism or an entire bacterial family (3, 5, 30). This approach, which allows for a comprehensive analysis of multiple genomes aiming at defining variability and diversity, has been validated for model organisms like *E. coli* (31), for plants, and more recently for vertebrates, including humans (32).

This study provides an up-to-date and complete pangenome analysis of *P. polymyxa*, in so far as it includes all *P. polymyxa* genomes currently publicly available (*N* = 108), plus the genomes of the five strains named MES17, 20, 108, 110, and 972 isolated at the University of Camerino (Italy), with the aim of generating a solid reference panel for the characterization of new *P. polymyxa* strains.

An average nucleotide identity cutoff of 95%–96% is commonly used to separate bacterial species based on their genome sequences (33, 34), even if this cutoff is rarely checked. Another problem is the lack of systematic comparison of newly sequenced genomes to type strains. In fact, species names are assigned by genome assembly uploaders in the NCBI assembly database often in the absence of microbiological comparisons, based only on DNA sequence analysis. As a matter of fact, the taxonomy check status at NCBI for several *P. polymyxa* strains considered in this work is reported as inconclusive. For these reasons, we applied strict QC rules to provide a robust framework of the *P. polymyxa* genomic and taxonomic features and phylogenomic treeing. Following these rules, we restricted our analysis to 57 strains from NCBI and 5 strains from the University of Camerino. More importantly, these analyses revealed that these strains form four distinct clusters that differ significantly in terms of ANI and dDDH percentages (Fig. 4), both considered as reference indices for separating bacterial species (35) when the similarity at the 16S DNA level is above 98%, as in the case of the analyzed strains. Notably, the presence of *P. ottowi* in Cluster 2 provides an additional valuable clue about the taxonomic and phylogenetic relationship existing among the various strains and clusters. In fact, strains ND24 and ZF129 from Cluster 2 presumably belong to the *P. ottowi* species, given their high degree of identity to the *P. ottowi* genome in terms of ANI and dDDH percentage. Starting from this consideration and observing the degree of relatedness between the various strains (Fig. 4), Cluster 1 likely forms a separate species from that of *P. polymyxa*. Regarding Clusters 3 and 4, the ANI and dDDH criteria for species boundary (95%–96% and 70%, respectively) would also indicate the presence of two different species. It should also be noted that the whole genome-based taxonomic analysis we conducted using TYGS (14) allowed us to associate the type strains *P. ottowi* and *P. polymyxa* ATCC 842 with Clusters 2 and 4, respectively, while the same analysis did not identify any closely related type strain for Clusters 1 and 3. In our opinion, these results could provide taxonomists with insights for reclassifying *P. polymyxa*. Based on our results, we propose maintaining the name *P. polymyxa* for Cluster 4, to which the type strain ATCC 842 belongs.

The main differences that distinguish *P. ottowii* from *P. polymyxa 842* do not concern cell morphology, substrate utilization, or fatty acid profiles but rather their DNA sequence/composition (8). In line with these considerations, no significant difference was detected between strains ATCC 842T, DSM 292, and DSM 365 using classical investigation approaches (21), but only by means of mass MALDI-TOF fingerprint profiles. Considering these data, we applied rMLST analysis to the *P. polymyxa* strains and verified that this typing method is useful for separating the strains into the four clusters. Based on the results of this analysis, we propose the sequencing of the amplicons of some ribosomal protein-encoding genes and/or the development of allele-specific PCRs (see Fig. S8 to S12) as a simple strategy to distinguish these different possible species.

The analysis of the pangenome conducted with Roary on the four different clusters has highlighted similarities and differences. First, all four groups have an open pangenome (*α* < 1). Comparing the genes (core, soft core, shell, and cloud) of the two most populated clusters, namely 1 and 4, it is possible to hypothesize that Cluster 1 is more heterogeneous than Cluster 4, having an overall lower number of core genes and a higher number of accessory genes. According to the Parsnp analysis, also the core genomes of Cluster 1 are more dissimilar than those of Cluster 4 (File S1; Fig. S5 to S7). On the other hand, this greater heterogeneity at both the gene type and sequence level is not surprising considering that Cluster 1 is composed of three subgroups (Fig. 4), unlike Cluster 4.

Regarding the new MES strains we isolated, all analyses performed on the genomes (ANI, dDDH, and rMLST) and the pangenome (Roary and Parsnp) indicate that the new five strains belong to Cluster 1. Specifically, there is a close relationship between strains MES17 and MES20, a phylogenetic proximity between these and strain MES108, while strains MES110 and MES972 have less relatedness compared to the others and to each other.

Functional analysis based on the COG categories showed a reduction of genes not attributable to any COG in the core genome of Cluster 1 compared to the other clusters (Fig. 7). Groups of genes, which we have called type-core (File S5), have also been identified as distinctive of the core genome of each cluster, which could provide a “fingerprint” for each of the four species. In addition to providing the list of these genes (File S5), we verified that the type-core of Clusters 2 and 3 are enriched with the genes not assigned to any COG. Unfortunately, the current scarce knowledge about these proteins does not allow us to make predictions about possible characteristics acquired through the presence/interactions of these genetic materials. Interestingly, the type-core of Clusters 1 and 4 were found to be enriched with genes involved in the biosynthesis of secondary metabolites. By employing the antiSMASH suite (Fig. 8), we verified that Cluster 4, compared to Cluster 1, is characterized by a statistically significantly higher number of potential producer strains of paenilan and paenibacillin. Interestingly, both compounds belong to the group I bacteriocins (lantibiotics), which are a specific class of toxic peptides produced by Gram-positive bacteria; paenilan was first isolated in *P. polymyxa E681* (36), while paenibacillin was isolated in *P. polymyxa OSY-DF* (37). Notably, specific peaks in a region of the mass spectra of *P. polymyxa* associated with antimicrobial compounds were found in the spectrum of ATCC 842 (Cluster 4) but not in those of DSM 292 and DSM 365 (Cluster 2) (21).

Some of the analyzed strains carry plasmids and megaplasmids. BLAST alignments allowed us to hypothesize the presence of plasmids in contig-level genome assemblies of eight strains (1-43, H4K, CICC 10580, NRRLB 30509, TH2H2, and hob6) for which there is no clear direct evidence of the presence of these mobile genetic elements. A peculiar situation arises with plasmid pPpO211. This plasmid does not have any known genes associated with its replication/segregation, yet its DNA sequence shares a high level of identity, throughout its length, with chromosomal regions of various strains. However, plasmid pPpO211 shares only a limited region (~25,000 bp) with the O2.11 chromosome. Very rarely, a chromosome can split into two parts generating a second chromosome (29), and this does not appear to be the case for pPpO211, as its genes group into functional classes typical of plasmids rather than chromosomes. It remains to be understood whether this is a genomic assembly error or if the above-mentioned strains have integrated a plasmid that has excised itself in O2.11.

*P. polymyxa* is a bacterium with broad application potential due to its ability to promote plant growth and produce molecules with antibiotic properties. Similarly, the five new bacterial MES strains have shown the ability to synthesize secondary metabolites capable of inhibiting the growth of a range of tester bacteria, including pathogens, such as *S. aureus* ATCC 25923 and *K. pneumoniae* ATCC 13883 (Fig. 2). The surge in multidrug-resistant bacteria poses a growing concern, standing as one of the main threats to global public health. Hence, efforts to introduce novel compounds and innovative screening methods, seeking untapped solutions and overlooked targets, represent crucial steps to successfully counteract this pressing health issue (38).

In conclusion, the comparative genomic analysis conducted in this work provides new insights into the genomic content and variability of *P. polymyxa*. The approach used made it possible to efficiently classify newly sequenced strains and distinguish strains with different properties and characteristics, offering the possibility of a more efficient classification. The analysis of the pangenome (5, 6) and the comparison of the genomes of *P. polymyxa* strains (39) had already been undertaken, revealing inconsistencies in the species classification. With a larger number of genomes now available, our work not only reaffirms previous findings but also lends robustness to them, expanding on earlier studies by integrating the results of ANI, dDDH, OrthoFinder, and rMLST analyses with those of the pangenome. During the revision of this article, a study on the pangenome of *P. polymyxa* (40) was published, showing results consistent with those presented and discussed here, confirming the validity of the data provided and the need to reclassify the strains grouped within this species.

## MATERIALS AND METHODS

### Isolation of MES strains

Strains MES17 and MES20 were isolated from samples of soil containing plant root fragments collected in Urbino (Italy, 43°43′31″N 12°38′14″E), strains MES108 and MES110 were from transitional water samples collected in the province of Latina (Italy, 41°28′02″N 12°54′13″E), while MES972 was isolated from the roots of an ancient oak tree located in the province of Macerata (Italy, 43°18′01″N 13°27′12″E). Isolation of MES17, 20, 108, and 110 was carried out on Luria-Bertani medium at 15°C for 7 days, while MES972 was isolated on tryptone soy agar (TSA) (30 g/L of Tryptone Soya Broth, Oxoid, supplemented with 17 g/L agar) at 30°C for 4 days. Selected colonies were re-streaked on the same media for further isolation and purification. All these mesophilic strains (MES) belong to the Culture Collection of Microorganisms established at the University of Camerino (Italy).

### Microbiological characterization of MES strains

The growth of the MES strains was tested at 15°C or 30°C by streaking 10 µL of cells at 0.2 OD_600_ on LB, TSA, or R2A (Reasoner’s 2A agar) media (Thermo Fisher Scientific), following colony growth for 4 days, or in 5% CO_2_ at 30°C on LB plates for 18–24 hours. Pictures of single colonies isolated on LB plates and grown at 15°C were taken by using a Lumix DMC-FZ1000 Panasonic camera under an inverted microscope (Axiovert 25 Microscope, Zeiss) using a 5× magnification.

### Biochemical characterization of MES strains

Utilization of carbon sources was assessed by API 50CH test according to the manufacturer (bioMérieux) using MES strains grown on LB plates. The test interpretation was carried out by APIweb software (bioMérieux).

Starch hydrolysis, indole production, trehalose fermentation, and citrate utilization were tested according to the protocols of the American Society for Microbiology [starch agar protocol (https://asm.org/Protocols/Starch-Agar-Protocol), indole test (https://asm.org/Protocols/Indole-Test-Protocol), carbohydrate fermentation protocol (https://asm.org/Protocols/Carbohydrate-Fermentation-Protocol), and citrate test protocol (https://asm.org/Protocols/Citrate-Test-Protocol), respectively].

### Antimicrobial testing by replica plating

The potential production of secondary metabolites with antibacterial activities by MES strains was assessed by inhibition growth assay. Each MES strain was grown as a spot of 20 mm at the center of an LB plate at 16°C for 5 days. In parallel, an aliquot of each tester strain from cultures grown until Abs_600nm_= 0.1 OD (see below) was homogeneously distributed by a sterile swab on an LB plate and incubated overnight at 37°C to produce a master plate. Each tester organism was then transferred by replica plating on the plate with the spotted MES strain. After incubation at 30°C for 24 hours, antibacterial activity was recorded in terms of size of inhibition halo. The bacterial testers were *Escherichia coli* ATCC 25922, *Staphylococcus aureus* ATCC 25923, *Klebsiella pneumoniae* ATCC13883, and *Bacillus subtilis* ATCC6633.

### Genome sequencing and annotation

Genomic DNA was extracted using the chromosomal DNA extraction kit from Sangon Biotech. Whole-genome sequencing was performed by IGAtech (Italy) using an Illumina NovaSeq6000 instrument on paired-end 150-bp mode (∼5.86 Mb; approximately 100-fold coverage). Read quality was assessed using FastQC (41). After trimming and merging paired reads with Trimmomatic version 0.38.1 (42), *de novo* assembly was carried out using Unicycler version 0.5.0 (43), followed by assembly quality check with QUAST version 5.2.0 (44). Genome annotation was carried out using the Prokaryotic genome annotation tool Prokka version 1.14.6 (45).

### Publicly available genome data retrieval, QC, and annotation

The complete genome sequence of the *P. polymyxa* strains was downloaded from NCBI, under the section “*Assembly*,” by querying for “*Paenibacillus polymyxa*.” Genomes were excluded according to NCBI abnormalities. Genome completeness was assessed using Benchmarking Universal Single-Copy Orthologs (10) version 5.4.4 (*Bacillales* data set odb10 and default parameters). All details regarding sample identification, isolation source, sequencing, exclusion, and assembly are described in File S4. Genomes retrieved from NCBI were annotated using Prokka using default settings (45). Information on the presence of plasmids was retrieved from the NCBI page for each strain.

### Phylogenomic and nucleotide identity analysis

We first determined the phylogenetic relationship between the strains retrieved using OrthoFinder version 2.5.4 (11) with predefined parameters, using the protein sequences obtained from the Prokka annotation. Next, average nucleotide identity analysis was performed using FastANI version 1.33 (12), with predefined parameters, using nucleotide sequences retrieved directly from NCBI. The phylogeny of these strains was further analyzed with autoMLST (13). Genome sequence data were also uploaded to the TYGS web server, for a whole genome-based taxonomic analysis (14). Digital DNA-DNA Hybridization values and confidence intervals were calculated using the recommended settings, and the pairwise comparisons of dDDH results were plotted using iTOL version 6.8.1 (46).

### rMLST analysis

PubMLST (23) was searched to produce the list of *P. polymyxa* available in the database. iTol version 6.8.1 (46) was then used to generate the neighbor-joining tree by selecting all ribosomal loci as the analysis criterion and the isolates retrieved from the PubMLST database corresponding to our strains of interest. Strains that were not present in the database were manually uploaded. To produce the multiple alignments shown in File S1, the sequences of the ribosomal genes and proteins of interest were downloaded from PubMLST, while the alignments were produced using Clustal Omega (47).

### Pangenome analysis

After determining the presence of potential confounding strains, strains were analyzed with Roary version 3.13.0 (18), using default settings, to perform pangenome analysis using GFF3 files generated by Prokka (45). Following the analysis, the genes were classified into four different classes: core (99% ≤ strains ≤ 100%), soft core (95% ≤ strains < 99%), shell (15% ≤ strains < 95%), and cloud (0% ≤ strains < 15%). Specifically, for Cluster 1, the adopted criteria were as follows: core genome: genes present in at least 23/24 strains; soft core: genes present in 22/24 strains; shell genome: genes present in 3–21 strains; cloud genome: genes present in fewer than three strains. For Cluster 2, the adopted criteria were as follows: core and soft core: genes present in at least 2/3 strains; shell genome: genes present in 1/3 strains; cloud genes: not present. For Cluster 3, the adopted criteria were as follows: core and soft core: genes present in at least 6/7 strains; shell genome: genes present in two to five strains; cloud genes present in one strain. For Cluster 4, the adopted criteria were as follows: core genome: genes present in at least 27/28 strains; soft core: genes present in 26/28 strains; shell genome: genes present in 4–25 strains; cloud genome: genes present in fewer than four strains. The openness of the pangenome was calculated by fitting the power law model according to the formula Δ*n* = *κN*^-*α*^, where Δ*n* is the number of newly added genes, *N* is the number of genomes used, and *κ* and *α* are the fitting parameters. For *α* > 1, the pangenome is closed, and for α < 1, the pangenome is open. The phylogeny of the strains in each cluster was further determined using both Roary (18) and Parsnp version 1.6.2 (20).

### Core genome annotation by EggNogg Mapper

The core genomes obtained from the pangenome analysis of the *P. polymyxa* strains underwent a further annotation round to determine functional differences across the four identified strain clusters. To this aim, we employed the EggNOG Mapper version 2 (24) searching against the EggNOG 5 database. The tool relies on Orthologous Groups from the EggNOG database and generates a list of annotated genes with an associated COG category. The lists of annotated genes belonging to the four core genomes were analyzed in three distinct ways. First, the identification of common/unique genes among the different clusters was performed both through queries on Excel sheets and using the Jvenn tool (19). In addition, the number of genes attributed to each COG category was used to test for statistically significant differences across the four core genome clusters or across the type-core genes (by means of Chi-square test), or between type-core and core genes belonging to the same cluster (using both Chi-square and Fisher’s exact test).

### Identification of gene clusters for secondary metabolites production

*P. polymyxa* genomes were analyzed to predict secondary metabolites production using the antiSMASH version 7 software (27). The Biosynthetic Gene Clusters detected by antiSMASH in the *P. polymyxa* strains were selected to keep only the BGCs exhibiting a similarity exceeding 80% with recognized BGCs from the Minimum Information about a Biosynthetic Gene Cluster version 2.0 repository (48). Their frequency in each cluster was then expressed both as a ratio (number of BGCs/total number of strains in that cluster) and as a percentage relative to the overall number of strains in the cluster.

### Screening of genes for antibiotic resistance

The core genomes of *P. polymyxa* were analyzed for the presence of antibiotic resistance genes by means of the web tool CARD version 3.2.6 (28) using RGI version 6.0.1 as an analysis option by selecting “Strict” and “Perfect” as search criteria. The CARD algorithm predicts a potential resistome for each genomic or plasmid sequence, genomic cluster, or whole-genome shotgun assembly using the anti-microbial resistance (AMR) detection patterns curated in CARD and the “Perfect” and “Strict” annotations of RGI, where the “Perfect” algorithm detects perfect matches with reference sequences and CARD-curated mutations, while the “Strict” algorithm uses CARD-curated bit-score cutoffs to predict known AMR variants, including key mutation screens. The threshold for the minimum identity rate was set at 50% (49).

### Plasmid analysis

The RefSeq FASTA files of the plasmid sequences were retrieved from the NCBI genome assembly web page for each strain and downloaded. The identification of the genes associated with specific functions was carried out by querying the NCBI files annotated in GenBank format for key terms. For ortholog detection, the identified protein sequences were used as queries in BLASTp (NCBI) alignments vs the nr database, using the default setting, while the comparison at nucleotide level was performed with BLASTn suite (NCBI) by uploading the plasmid fasta files. For the MES strains not included in the NCBI database, NCBI BLAST+ makeblastdb version 2.14.1 (50) was used to create a BLAST database starting from the *.faa files, which was searched using the above-mentioned protein sequences as queries. The search for the relaxation-replication motif in the MES strains was also carried out using PfamScan version 1.6 (51), searching the genomic sequences against the Pfam HMM PF13814.9 data set. Finally, the GC content of each plasmid was determined using geecee version 5.0.0 (52).

### Statistical analysis

Fisher’s exact test and Chi-square test were applied to contingency tables using GraphPad Prism 9.3 software (GraphPad Software, USA), with a statistical significance threshold set at *P* < 0.05 (two-tailed).

## Data Availability

The complete genome sequences of the MES strains isolated at the University of Camerino (Camerino, Italy) described here have been deposited in NCBI GenBank under BioProject number PRJNA1049263 and BioSample numbers SAMN38691874, SAMN38691875, SAMN38691876, SAMN38691877, and SAMN38691878.

## SUPPLEMENTAL MATERIAL

File S1 aem.01740-24-s0001.pdf Table S1; Figures S1 to S12.

File S2 aem.01740-24-s0002.xlsx Complete matrix of ANI values.

File S3 aem.01740-24-s0003.xlsx Complete matrix of dDDH values.

File S4 aem.01740-24-s0004.xlsx Strain information, including initial full data set and final QC'd data set.

File S5 aem.01740-24-s0005.xlsx Core genes, common genes, and type-core genes in the four clusters.

ASM does not own the copyrights to Supplemental Material that may be linked to, or accessed through, an article. The authors have granted ASM a non-exclusive, world-wide license to publish the Supplemental Material files. Please contact the corresponding author directly for reuse.
