# Supplementary material for: Pangenome analysis of Paenibacillus polymyxa strains reveals the existence of multiple and functionally distinct Paenibacillus species
Source: Appl Environ Microbiol. 2024 Oct 30;90(11):e01740-24. doi: 10.1128/aem.01740-24 (PMC11577871; doi:10.1128/aem.01740-24)
Supplement: File S1 — Table S1; Figures S1 to S12. [file aem.01740-24-s0001.pdf]

Table S1. Assembly statistics

	MES17	MES20	MES108	MES110	MES972
# paired-end reads	15,827,092	8,236,434	6,499,124	21,062,244	9,296,048
Sequencing depth	~400X	~210X	~170X	~540x	~230X
# contigs[a]	92	90	105	57	70
Total length[a]	5,858,763	5,857,725	5,668,879	5,808,840	6,081,788
GC (%)[a]	45.40	45.39	45.57	45.50	45.14
N50[a]	259,003	265,343	261,401	413,718	235,203
Largest contig[a]	714,337	714,337	672,509	918,158	1,237,086
CDS[b]	5,286	5,288	5,120	5,175	5,536
tRNAs[b]	93	93	83	87	88
tmRNA[b]	1	1	1	1	1
rRNAs	incomplete	incomplete	incomplete	incomplete	incomplete

[a]Statistics based on contigs of size >= 500 bp (Quast report)

[b] Based on Prokka annotation

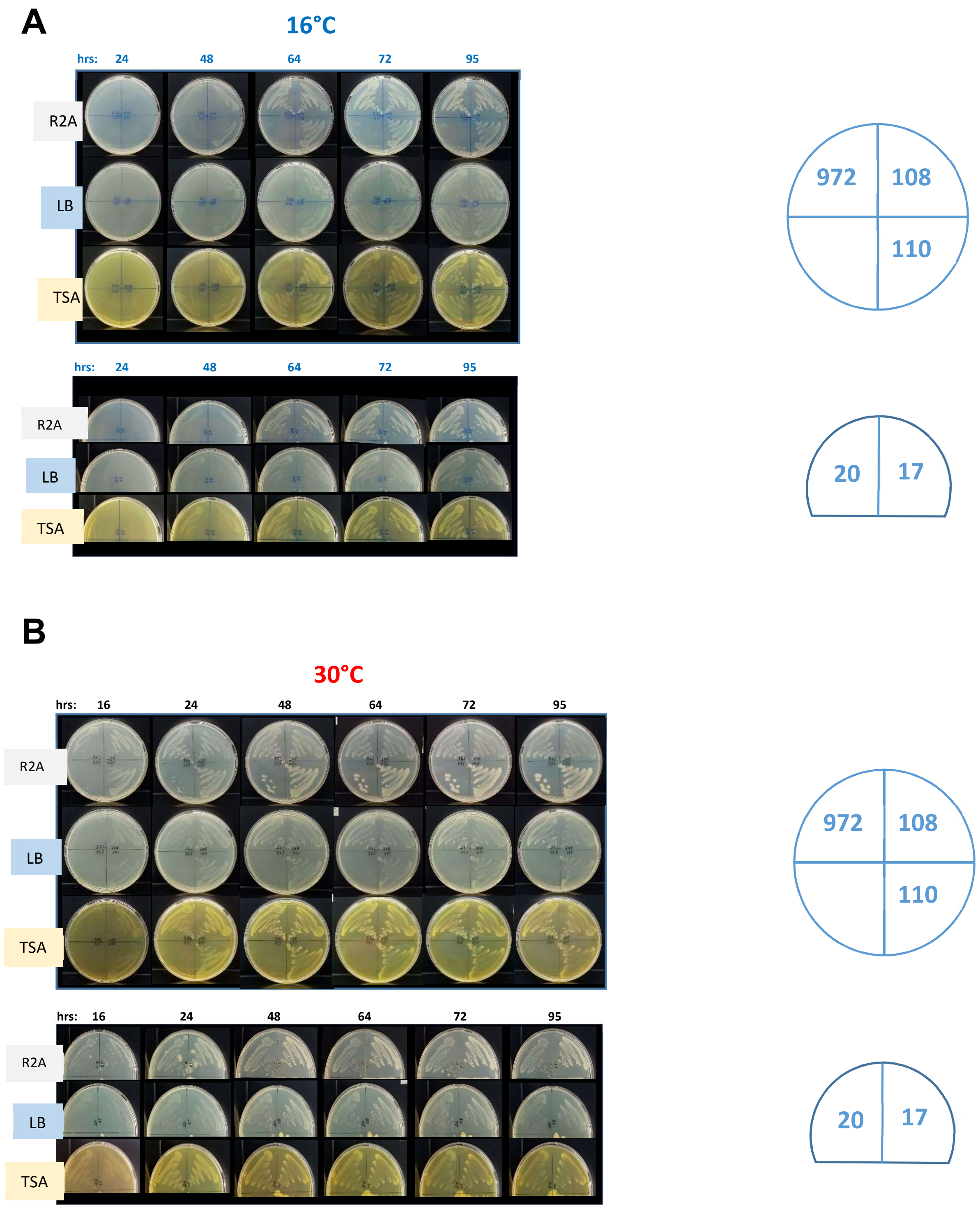

Figure S1. Photographs of MES strains grown at the indicated time points on LB, TSA, or R2A media at 15°C (A) and 30°C (B). The accompanying diagram indicates which strain was grown on the respective section of the plate.

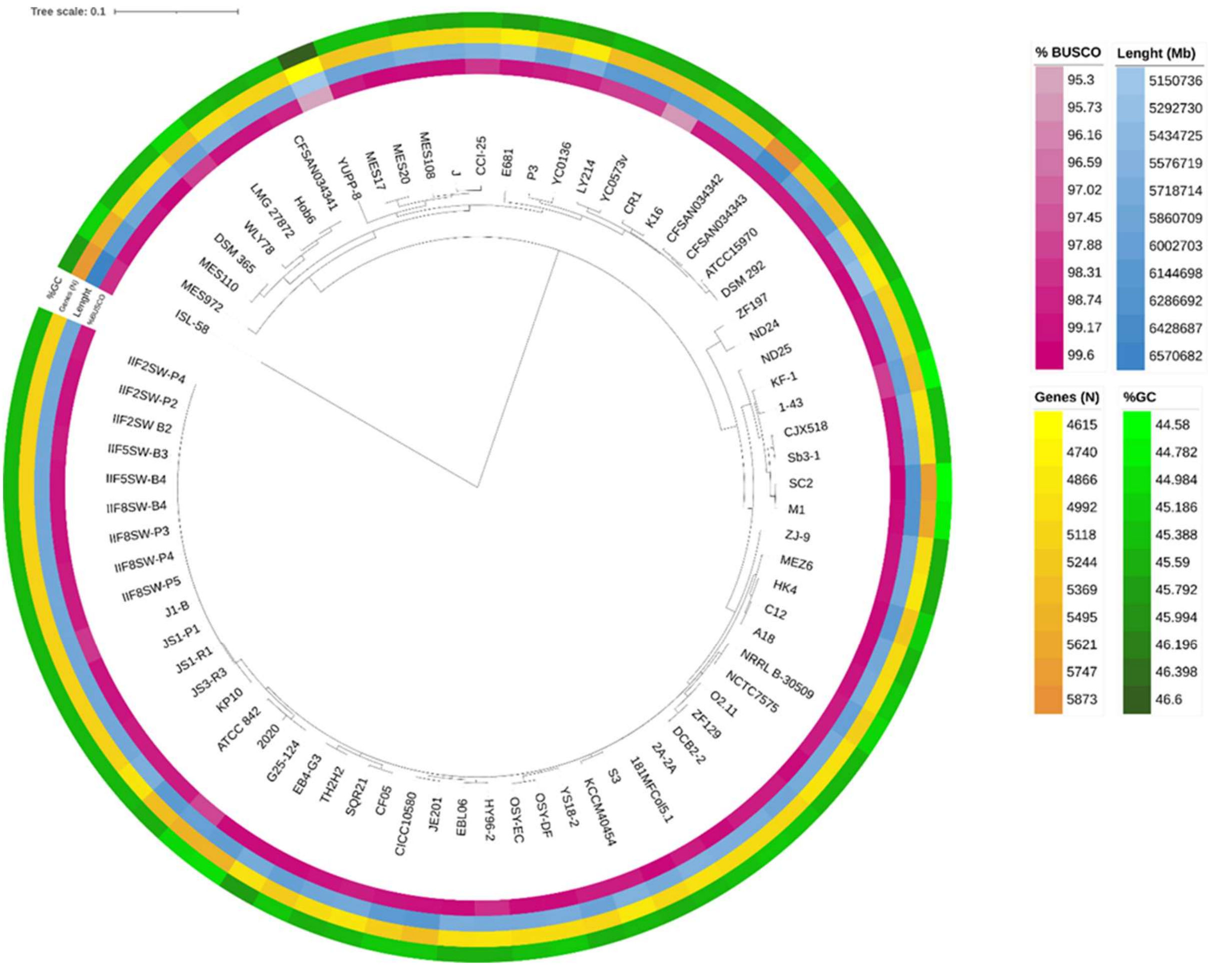

Figure S2. Phylogenetic tree features of the 76 strains of *P. polymyxa* obtained with Orthofinder. The circular heatmaps from inside to outside show the percentage of BUSCO, genome size, number of genes, and GC content, for each strain.

A

B

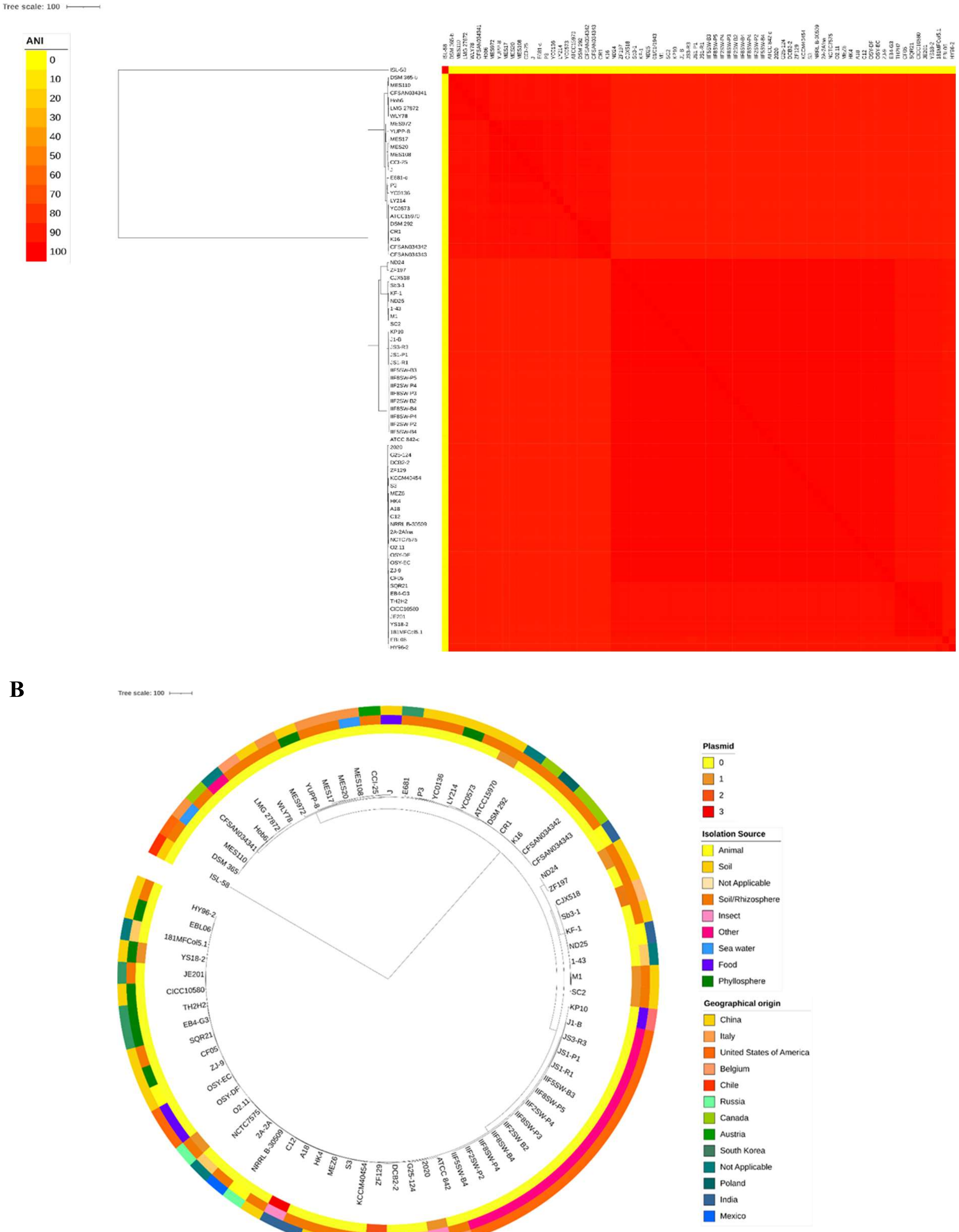

Figure S3. Correlation matrix of the 76 strains of *P. polymyxa* obtained with FastANI. A) In red strains with low nucleotide similarity, in yellow strains with high similarity. B) The circular heatmaps from inside to outside show the number of plasmids, isolation source and geographical origin, for each strain

A

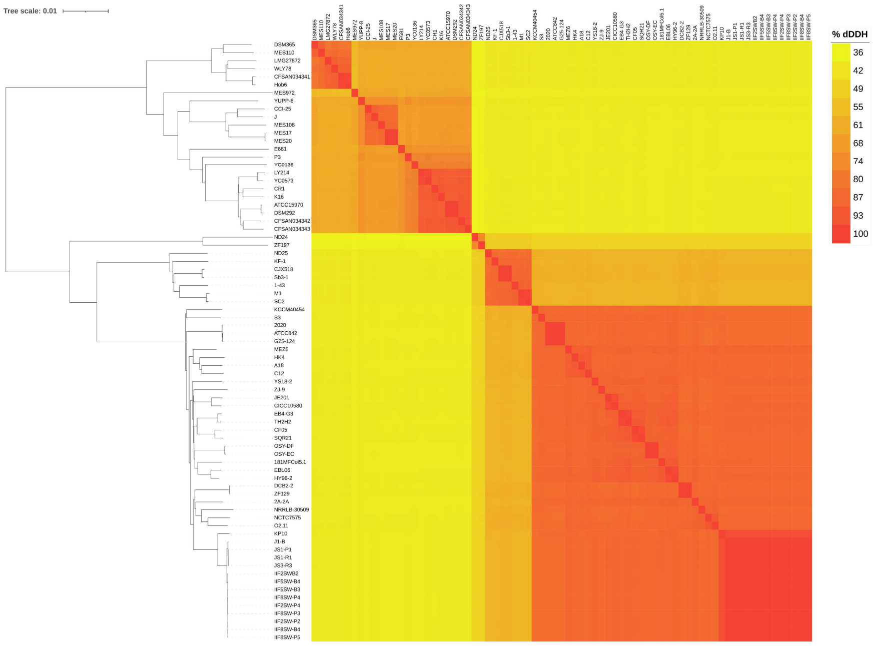

B

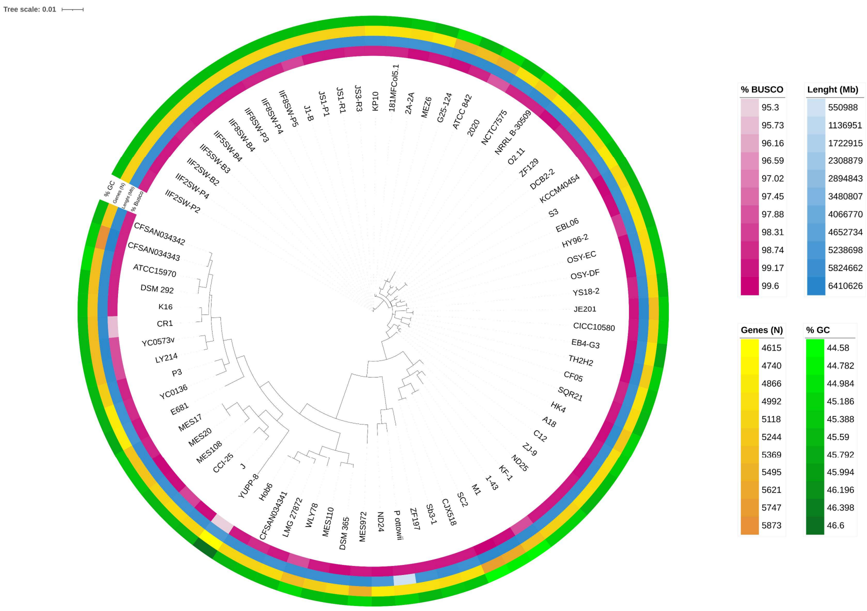

Figure S4. A) Heatmap of digital DNA-DNA hybridization values of the 75 genomes of *P. polymy*xa. B) Phylogenetic tree features of the 75 strains of *P. polymyxa* and *P. ottowii* obtained with OrthoFinder. The circular heatmap from inside to outside show the percentage of BUSCO, genome size, number of genes, and GC content, for each strain.

A

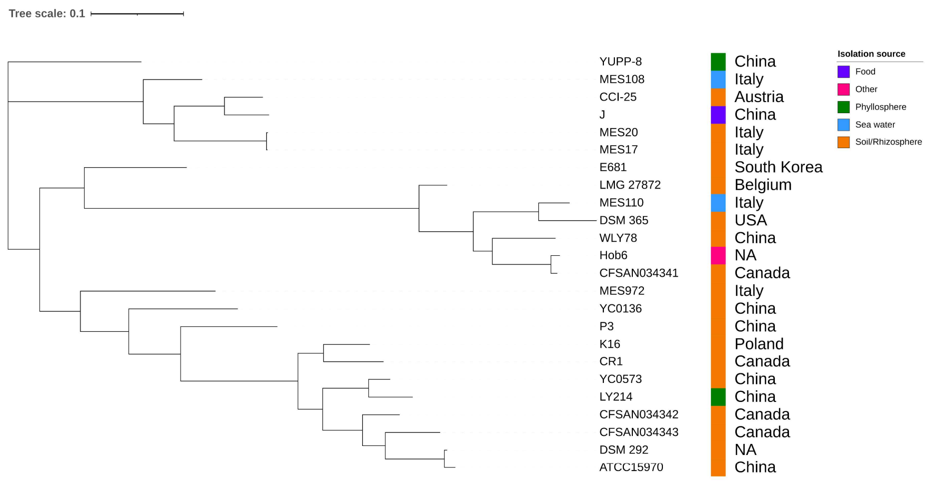

B

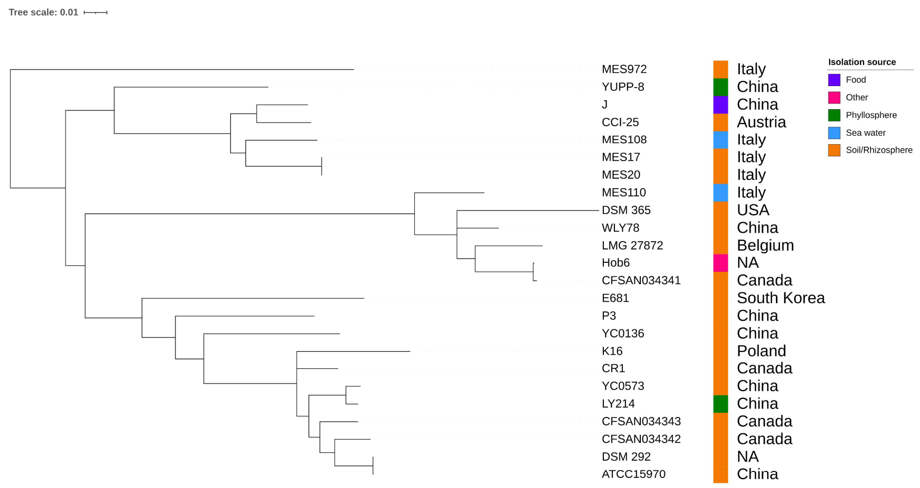

Figure S5. Phylogenetic relationships of Cluster 1 strains obtained with Roary (A) or Parsnp (B).

A

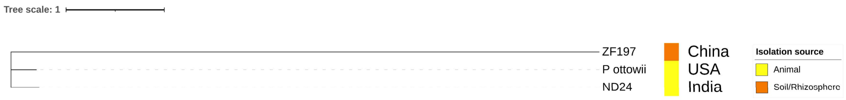

B

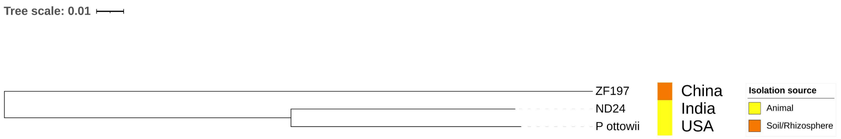

C

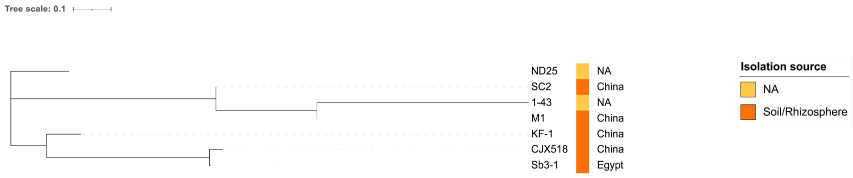

D

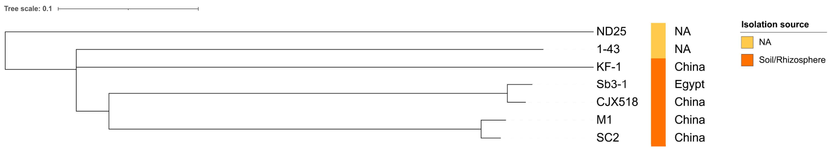

Figure S6. Phylogenetic relationships of Cluster 2 (A and B) and Cluster 3 (C and D) strains obtained with Roary (A and C) or Parsnp (B and D).

A

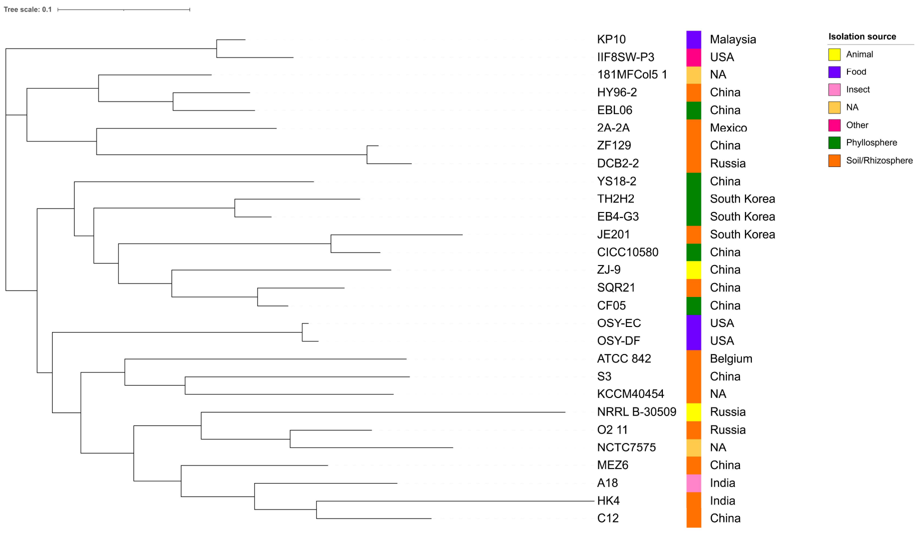

B

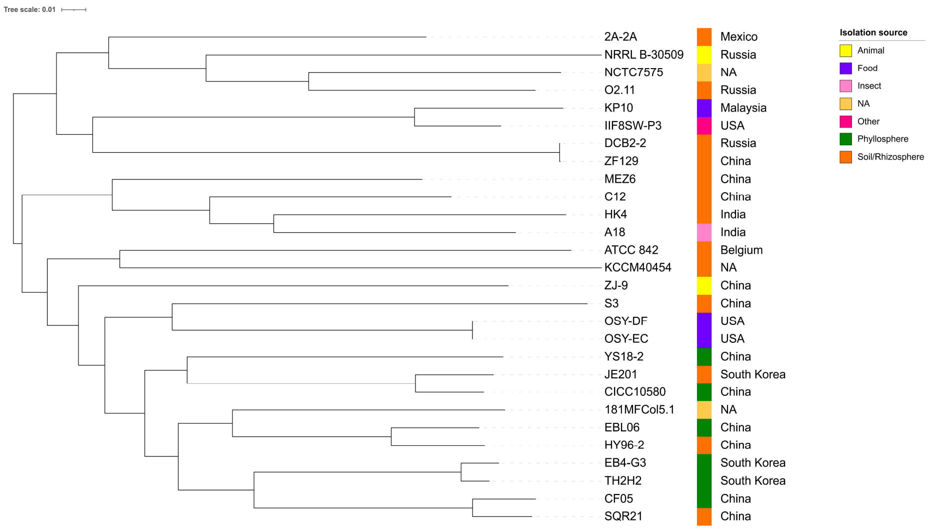

Figure S7. Phylogenetic relationships of Cluster 4 strains obtained with Roary (A) or Parsnp (B).

rplM

```
MES972      ATGCGTACTACGTATATGGCGAAGCCAAATGAAGTTGAGCGCAACTGGCACATCATTGAT GCCGAAGGCAAAACGCTCGGCCGTCTGGCAAGCGAAGCGGCTGCGTTGATTCGCGGCAAA
DSM_365     ATGCGTACTACGTATATGGCGAAGCCAAATGAAGTTGAGCGCAACTGGCACATCATTGAT GCCGAAGGCAAAACGCTCGGTCGTCTGGCAAGCGAAGCGGCTGCGTTGATTCGCGGCAAA
WLY78       ATGCGTACTACGTATATGGCGAAGCCAAATGAAGTTGAGCGCAACTGGCACATCATTGAT GCCGAAGGCAAAACGCTCGGTCGTCTGGCAAGCGAAGCGGCTGCGTTGATTCGCGGCAAA
MES110      ATGCGTACTACGTATATGGCGAAGCCAAATGAAGTTGAGCGCAACTGGCACATCATTGAT GCCGAAGGCAAAACGCTCGGTCGTCTGGCAAGCGAAGCGGCTGCGTTGATTCGCGGCAAA
CFSAN0343   ATGCGTACTACGTATATGGCGAAGCCAAATGAAGTTGAGCGCAACTGGCACATCATTGAT GCCGAAGGCAAAACGCTCGGTCGTCTGGCAAGCGAAGCGGCTGCGTTGATTCGCGGCAAA
Hob6        ATGCGTACTACGTATATGGCGAAGCCAAATGAAGTTGAGCGCAACTGGCACATCATTGAT GCCGAAGGCAAAACGCTCGGTCGTCTGGCAAGCGAAGCGGCTGCGTTGATTCGCGGCAAA
LMG_27872   ATGCGTACTACGTATATGGCGAAGCCAAATGAAGTTGAGCGCAACTGGCACATCATTGAT GCCGAAGGCAAAACGCTCGGTCGTCTGGCAAGCGAAGCGGCTGCGTTGATTCGCGGCAAA
E681        ATGCGTACTACGTATATGGCGAAGCCAAATGAAGTTGAGCGCAACTGGCACATCATTGAT GCCGAAGGCAAAACGCTCGGCCGTCTGGCAAGCGAAGCGGCTGCGTTGATTCGCGGCAAA
J           ATGCGTACTACGTATATGGCGAAGCCAAATGAAGTTGAGCGCAACTGGCACATCATTGAT GCCGAAGGCAAAACGCTCGGCCGTCTGGCAAGCGAAGCGGCTGCGTTGATTCGCGGCAAA
YC0573      ATGCGTACTACGTATATGGCGAAGCCAAATGAAGTTGAGCGCAACTGGCACATCATTGAT GCCGAAGGCAAAACGCTCGGCCGTCTGGCAAGCGAAGCGGCTGCGTTGATTCGCGGCAAA
LY214       ATGCGTACTACGTATATGGCGAAGCCAAATGAAGTTGAGCGCAACTGGCACATCATTGAT GCCGAAGGCAAAACGCTCGGCCGTCTGGCAAGCGAAGCGGCTGCGTTGATTCGCGGCAAA
CR1         ATGCGTACTACGTATATGGCGAAGCCAAATGAAGTTGAGCGCAACTGGCACATCATTGAT GCCGAAGGCAAAACGCTCGGCCGTCTGGCAAGCGAAGCGGCTGCGTTGATTCGCGGCAAA
K16         ATGCGTACTACGTATATGGCGAAGCCAAATGAAGTTGAGCGCAACTGGCACATCATTGAT GCCGAAGGCAAAACGCTCGGCCGTCTGGCAAGCGAAGCGGCTGCGTTGATTCGCGGCAAA
CFSAN0343   ATGCGTACTACGTATATGGCGAAGCCAAATGAAGTTGAGCGCAACTGGCACATCATTGAT GCCGAAGGCAAAACGCTCGGCCGTCTGGCAAGCGAAGCGGCTGCGTTGATTCGCGGCAAA
CFSAN0343   ATGCGTACTACGTATATGGCGAAGCCAAATGAAGTTGAGCGCAACTGGCACATCATTGAT GCCGAAGGCAAAACGCTCGGCCGTCTGGCAAGCGAAGCGGCTGCGTTGATTCGCGGCAAA
ATCC_1597   ATGCGTACTACGTATATGGCGAAGCCAAATGAAGTTGAGCGCAACTGGCACATCATTGAT GCCGAAGGCAAAACGCTCGGCCGTCTGGCAAGCGAAGCGGCTGCGTTGATTCGCGGCAAA
DSM_292     ATGCGTACTACGTATATGGCGAAGCCAAATGAAGTTGAGCGCAACTGGCACATCATTGAT GCCGAAGGCAAAACGCTCGGCCGTCTGGCAAGCGAAGCGGCTGCGTTGATTCGCGGCAAA
MES20       ATGCGTACTACGTATATGGCGAAGCCAAATGAAGTTGAGCGCAACTGGCACATCATTGAT GCCGAAGGCAAAACGCTCGGCCGTCTGGCAAGCGAAGCGGCTGCGTTGATTCGCGGCAAA
MES17       ATGCGTACTACGTATATGGCGAAGCCAAATGAAGTTGAGCGCAACTGGCACATCATTGAT GCCGAAGGCAAAACGCTCGGCCGTCTGGCAAGCGAAGCGGCTGCGTTGATTCGCGGCAAA
MES108      ATGCGTACTACGTATATGGCGAAGCCAAATGAAGTTGAGCGCAACTGGCACATCATTGAT GCCGAAGGCAAAACGCTCGGCCGTCTGGCAAGCGAAGCGGCTGCGTTGATTCGCGGCAAA
YUPP_8      ATGCGTACTACGTATATGGCGAAGCCAAATGAAGTTGAGCGCAACTGGCACATCATTGAT GCCGAAGGCAAAACGCTCGGCCGTCTGGCAAGCGAAGCGGCTGCGTTGATTCGCGGCAAA
YC0136      ATGCGTACTACGTATATGGCGAAGCCAAATGAAGTTGAGCGCAACTGGCACATCATTGAT GCCGAAGGCAAAACGCTCGGCCGTCTGGCAAGCGAAGCGGCTGCGTTGATTCGCGGCAAA
P3          ATGCGTACTACGTATATGGCGAAGCCAAATGAAGTTGAGCGCAACTGGCACATCATTGAT GCCGAAGGCAAAACGCTCGGCCGTCTGGCAAGCGAAGCGGCTGCGTTGATTCGCGGCAAA
CCI_25      ATGCGTACTACGTATATGGCGAAGCCAAATGAAGTTGAGCGCAACTGGCACATCATTGAT GCCGAAGGCAAAACGCTCGGCCGTCTGGCAAGCGAAGCGGCTGCGTTGATTCGCGGCAAA
ND24        ATGCGTACTACGTATATGGCGAAGCCAAATGAAGTTGAGCGCAACTGGCACATCATTGAT GCCGAAGGCAAAACGCTCGGCCGTCTGGCAAGCGAAGCGGCTGCGTTGATCCGCGGCAAA
ZF197       ATGCGTACTACGTATATGGCGAAGCCAAATGAAGTTGAGCGCAACTGGCACATCATTGAT GCCGAAGGCAAAACGCTCGGCCGTCTGGCAAGCGAAGCGGCTGCGTTGATTCGCGGCAAA
P.ottowii   ATGCGTACTACGTATATGGCGAAGCCAAATGAAGTTGAGCGCAACTGGCACATCATTGAT GCCGAAGGCAAAACGCTCGGCCGTCTGGCAAGCGAAGCGGCTGCGTTGATTCGCGGCAAA
181MFCol5   ATGCGTACTACGTATATGGCGAAGCCAAATGAAGTTGAGCGCAATTGGCACATCATTGAT GCCGAAGGCAAAACGCTCGGCCGTCTGGCAAGCGAAGCGGCTGCGTTGATTCGCGGCAAA
SQR_21      ATGCGTACTACGTATATGGCGAAGCCAAATGAAGTTGAGCGCAATTGGCACATCATTGAT GCCGAAGGCAAAACGCTCGGCCGTCTGGCAAGCGAAGCGGCTGCGTTGATTCGCGGCAAA
CF05        ATGCGTACTACGTATATGGCGAAGCCAAATGAAGTTGAGCGCAATTGGCACATCATTGAT GCCGAAGGCAAAACGCTCGGCCGTCTGGCAAGCGAAGCGGCTGCGTTGATTCGCGGCAAA
CICC_1058   ATGCGTACTACGTATATGGCGAAGCCAAATGAAGTTGAGCGCAATTGGCACATCATTGAT GCCGAAGGTAAAACGCTCGGCCGTCTGGCAAGCGAAGCGGCTGCGTTGATTCGCGGCAAA
JE201       ATGCGTACTACGTATATGGCGAAGCCAAATGAAGTTGAGCGCAATTGGCACATCATTGAT GCCGAAGGTAAAACGCTCGGCCGTCTGGCAAGCGAAGCGGCTGCGTTGATTCGCGGCAAA
ATCC_842    ATGCGTACTACGTATATGGCGAAGCCAAATGAAGTTGAGCGCAATTGGCACATCATTGAT GCCGAAGGCAAAACGCTCGGTCGTCTGGCAAGCGAAGCGGCTGCGTTGATTCGCGGCAAA
S3          ATGCGTACTACGTATATGGCGAAGCCAAATGAAGTTGAGCGCAATTGGCACATCATTGAT GCCGAAGGCAAAACGCTCGGTCGTCTGGCAAGCGAAGCGGCTGCGTTGATTCGCGGCAAA
OSY_DF      ATGCGTACTACGTATATGGCGAAGCCAAATGAAGTTGAGCGCAATTGGCACATCATTGAT GCCGAAGGCAAAACGCTCGGCCGTCTGGCAAGCGAAGCGGCTGCGTTGATTCGCGGCAAA
OSY_EC      ATGCGTACTACGTATATGGCGAAGCCAAATGAAGTTGAGCGCAATTGGCACATCATTGAT GCCGAAGGCAAAACGCTCGGCCGTCTGGCAAGCGAAGCGGCTGCGTTGATTCGCGGCAAA
C12         ATGCGTACTACGTATATGGCGAAGCCAAATGAAGTTGAGCGCAATTGGCACATCATTGAT GCCGAAGGCAAAACGCTCGGCCGTCTGGCAAGCGAAGCGGCTGCGTTGATTCGCGGCAAA
NRRL_B_30   ATGCGTACTACGTATATGGCGAAGCCAAATGAAGTTGAGCGCAATTGGCACATCATTGAT GCCGAAGGCAAAACGCTCGGCCGTCTGGCAAGCGAAGCGGCTGCGTTGATTCGCGGCAAA
NCTC7575    ATGCGTACTACGTATATGGCGAAGCCAAATGAAGTTGAGCGCAATTGGCACATCATTGAT GCCGAAGGCAAAACGCTCGGCCGTCTGGCAAGCGAAGCGGCTGCGTTGATTCGCGGCAAA
MEZ6        ATGCGTACTACGTATATGGCGAAGCCAAATGAAGTTGAGCGCAATTGGCACATCATTGAT GCCGAAGGCAAAACGCTCGGCCGTCTGGCAAGCGAAGCGGCTGCGTTGATTCGCGGCAAA
O_211       ATGCGTACTACGTATATGGCGAAGCCAAATGAAGTTGAGCGCAATTGGCACATCATTGAT GCCGAAGGCAAAACGCTCGGCCGTCTGGCAAGCGAAGCGGCTGCGTTGATTCGCGGCAAA
HK4         ATGCGTACTACGTATATGGCGAAGCCAAATGAAGTTGAGCGCAATTGGCACATCATTGAT GCCGAAGGCAAAACGCTCGGCCGTCTGGCAAGCGAAGCGGCTGCGTTGATTCGCGGCAAA
A18         ATGCGTACTACGTATATGGCGAAGCCAAATGAAGTTGAGCGCAATTGGCACATCATTGAT GCCGAAGGCAAAACGCTCGGCCGTCTGGCAAGCGAAGCGGCTGCGTTGATTCGCGGCAAA
TH2H2       ATGCGTACTACGTATATGGCGAAGCCAAATGAAGTTGAGCGCAATTGGCACATCATTGAT GCCGAAGGCAAAACGCTCGGCCGTCTGGCAAGCGAAGCGGCTGCGTTGATTCGCGGCAAA
EB4_G3      ATGCGTACTACGTATATGGCGAAGCCAAATGAAGTTGAGCGCAATTGGCACATCATTGAT GCCGAAGGCAAAACGCTCGGCCGTCTGGCAAGCGAAGCGGCTGCGTTGATTCGCGGCAAA
DCB2        ATGCGTACTACGTATATGGCGAAGCCAAATGAAGTTGAGCGCAATTGGCACATCATTGAT GCCGAAGGCAAAACGCTCGGCCGTCTGGCAAGCGAAGCGGCTGCGTTGATTCGCGGCAAA
ZF129       ATGCGTACTACGTATATGGCGAAGCCAAATGAAGTTGAGCGCAATTGGCACATCATTGAT GCCGAAGGCAAAACGCTCGGCCGTCTGGCAAGCGAAGCGGCTGCGTTGATTCGCGGCAAA
KP10        ATGCGTACTACGTATATGGCGAAGCCAAATGAAGTTGAGCGCAATTGGCACATCATTGAT GCCGAAGGCAAAACGCTCGGCCGTCTGGCAAGCGAAGCGGCTGCGTTGATTCGCGGCAAA
IIF8SW      ATGCGTACTACGTATATGGCGAAGCCAAATGAAGTTGAGCGCAATTGGCACATCATTGAT GCCGAAGGCAAAACGCTCGGCCGTCTGGCAAGCGAAGCGGCTGCGTTGATTCGCGGCAAA
2A_2A       ATGCGTACTACGTATATGGCGAAGCCAAATGAAGTTGAGCGCAATTGGCACATCATTGAT GCCGAAGGCAAAACGCTCGGCCGTCTGGCAAGCGAAGCGGCTGCGTTGATTCGCGGCAAA
YS18        ATGCGTACTACGTATATGGCGAAGCCAAATGAAGTTGAGCGCAATTGGCACATCATTGAT GCCGAAGGCAAAACGCTCGGCCGTCTGGCAAGCGAAGCGGCTGCGTTGATTCGCGGCAAA
EBL06       ATGCGTACTACGTATATGGCGAAGCCAAATGAAGTTGAGCGCAATTGGCACATCATTGAT GCCGAAGGCAAAACGCTCGGCCGTCTGGCAAGCGAAGCGGCTGCGTTGATTCGCGGCAAA
HY96        ATGCGTACTACGTATATGGCGAAGCCAAATGAAGTTGAGCGCAATTGGCACATCATTGAT GCCGAAGGCAAAACGCTCGGCCGTCTGGCAAGCGAAGCGGCTGCGTTGATTCGCGGCAAA
ZJ_9        ATGCGTACTACGTATATGGCGAAGCCAAATGAAGTTGAGCGCAATTGGCACATCATTGAT GCCGAAGGCAAAACGCTCGGCCGTCTGGCAAGCGAAGCGGCTGCGTTGATTCGCGGCAAA
KCCM_4045   ATGCGTACTACGTATATGGCGAAGCCAAATGAAGTTGAGCGCAATTGGCACATCATTGAT GCCGAAGGCAAAACGCTCGGCCGTCTGGCAAGCGAAGCGGCTGCGTTGATTCGCGGCAAA
Sb3         ATGCGTACTACGTATATGGCGAAGCCAAATGAAGTTGAGCGCAACTGGCACATCATTGAT GCCGAAGGCAAAACGCTCGGCCGTCTGGCAAGCGAAGCGGCTGCGTTGATTCGCGGCAAA
CJX518      ATGCGTACTACGTATATGGCGAAGCCAAATGAAGTTGAGCGCAACTGGCACATCATTGAT GCCGAAGGCAAAACGCTCGGCCGTCTGGCAAGCGAAGCGGCTGCGTTGATTCGCGGCAAA
KF_1        ATGCGTACTACGTATATGGCGAAGCCAAATGAAGTTGAGCGCAACTGGCACATCATTGAT GCCGAAGGCAAAACGCTCGGCCGTCTGGCAAGCGAAGCGGCTGCGTTGATTCGCGGCAAA
ND25        ATGCGTACTACGTATATGGCGAAGCCAAATGAAGTTGAGCGCAACTGGCACATCATTGAT GCCGAAGGCAAAACGCTCGGCCGTCTGGCAAGCGAAGCGGCTGCGTTGATTCGCGGCAAA
1_43        ATGCGTACTACGTATATGGCGAAGCCAAATGAAGTTGAGCGCAACTGGCACATCATTGAT GCCGAAGGCAAAACGCTCGGCCGTCTGGCAAGCGAAGCGGCTGCGTTGATTCGCGGCAAA
SC2         ATGCGTACTACGTATATGGCGAAGCCAAATGAAGTTGAGCGCAACTGGCACATCATTGAT GCCGAAGGCAAAACGCTCGGCCGTCTGGCAAGCGAAGCGGCTGCGTTGATTCGCGGCAAA
M1          ATGCGTACTACGTATATGGCGAAGCCAAATGAAGTTGAGCGCAACTGGCACATCATTGAT GCCGAAGGCAAAACGCTCGGCCGTCTGGCAAGCGAAGCGGCTGCGTTGATTCGCGGCAAA
            ******************************************** *************** ******** *********** ***************************** *********
```

Cluster 1 AACTGGTGGCACATCATTGATGCCGAAGGC AAAACGCTCGG$^{C/T}$CGTCTGGCAAGCGAAGCGGCTGCGTTGATT CGCGGCAAA
Cluster 2 AACTGGTGGCACATCATTGATGCCGAAGGC AAAACGCTCGGC CGTCTGGCAAGCGAAGCGGCTGCGTTGAT$^{T/C}$CGCGGCAAA
Cluster 3 AACTGGTGGCACATCATTGATGCCGAAGGC AAAACGCTCGGC CGTCTGGCAAGCGAAGCGGCTGCGTTGATT CGCGGCAAA
Cluster 4 AATTGGTGGCACATCATTGATGCCGAAGG$^{C/T}$AAAACGCTCGG$^{C/T}$CGTCTGGCAAGCGAAGCGGCTGCGTTGATT CGCGGCAAA

```
MES972          CACAAGCCACAATTTACACCACATGTGGACACAGGTGATTTTGTTGTTGTAATCAATGCG GAAAAAATCGTATTGACCGGAAAAAAAAATGGAGAACAAGAAATACTATCGTCACTCCCTG
DSM_365         CACAAGCCACAATTTACACCACATGTGGACACAGGTGATTTTGTTGTTGTAATCAATGCG GAAAAAATCGTATTGACCGGTAAAAAAATGGAGAACAAGAAATACTACCGTCACTCCCTG
WLY78           CACAAGCCACAGTTTACACCACATGTGGACACAGGTGATTTTGTTGTTGTAATCAATGCG GAAAAAATCGTATTGACCGGTAAAAAAATGGAGAACAAGAAATACTACCGTCACTCCCTG
MES110          CACAAGCCACAGTTTACACCACATGTGGACACAGGTGATTTTGTTGTTGTAATCAATGCG GAAAAAATCGTATTGACCGGTAAAAAAATGGAGAACAAGAAATACTACCGTCACTCCCTG
CFSAN034341     CACAAGCCACAGTTTACACCACATGTGGACACAGGTGATTTTGTTGTTGTAATCAATGCG GAAAAAATCGTATTGACCGGTAAAAAAATGGAGAACAAGAAATACTACCGTCACTCCCTG
Hob6            CACAAGCCACAGTTTACACCACATGTGGACACAGGTGATTTTGTTGTTGTAATCAATGCG GAAAAAATCGTATTGACCGGTAAAAAAATGGAGAACAAGAAATACTACCGTCACTCCCTG
LMG_27872       CACAAGCCACAATTTACACCACATGTGGACACAGGTGATTTTGTTGTTGTAATCAATGCG GAAAAAATCGTATTGACCGGTAAAAAAATGGAGAACAAGAAATACTACCGTCACTCCCTG
E681            CACAAGCCACAATTTACACCACATGTGGACACAGGTGATTTTGTTGTTGTAATCAATGCG GAAAAAATCGTATTGACCGGTAAAAAAATGGAGAACAAGAAATACTACCGTCACTCCCTG
J               CACAAGCCACAATTTACACCACATGTGGACACAGGTGATTTTGTTGTTGTAATCAATGCG GAAAAAATCGTATTGACCGGTAAAAAAATGGAGAACAAGAAATACTACCGTCACTCCCTG
YC0573          CACAAGCCACAATTTACACCACATGTGGACACAGGTGATTTTGTTGTTGTAATCAATGCG GAAAAAATCGTATTGACCGGTAAAAAAATGGAGAACAAGAAATACTACCGTCACTCCCTG
LY214           CACAAGCCACAATTTACACCACATGTGGACACAGGTGATTTTGTTGTTGTAATCAATGCG GAAAAAATCGTATTGACCGGTAAAAAAATGGAGAACAAGAAATACTACCGTCACTCCCTG
CR1             CACAAGCCACAATTTACACCACATGTGGACACAGGTGATTTTGTTGTTGTAATCAATGCG GAAAAAATCGTATTGACCGGTAAAAAAATGGAGAACAAGAAATACTACCGTCACTCCCTG
K16             CACAAGCCACAATTTACACCACATGTGGACACAGGTGATTTTGTTGTTGTAATCAATGCG GAAAAAATCGTATTGACCGGTAAAAAAATGGAGAACAAGAAATACTACCGTCACTCCCTG
CFSAN034343     CACAAGCCACAATTTACACCACATGTGGACACAGGTGATTTTGTTGTTGTAATCAATGCG GAAAAAATCGTATTGACCGGTAAAAAAATGGAGAACAAGAAATACTACCGTCACTCCCTG
CFSAN034342     CACAAGCCACAATTTACACCACATGTGGACACAGGTGATTTTGTTGTTGTAATCAATGCG GAAAAAATCGTATTGACCGGTAAAAAAATGGAGAACAAGAAATACTACCGTCACTCCCTG
ATCC_15970      CACAAGCCACAATTTACACCACATGTGGACACAGGTGATTTTGTTGTTGTAATCAATGCG GAAAAAATCGTATTGACCGGTAAAAAAATGGAGAACAAGAAATACTACCGTCACTCCCTG
DSM_292         CACAAGCCACAATTTACACCACATGTGGACACAGGTGATTTTGTTGTTGTAATCAATGCG GAAAAAATCGTATTGACCGGTAAAAAAATGGAGAACAAGAAATACTACCGTCACTCCCTG
MES20           CACAAGCCACAATTTACACCACATGTGGACACAGGTGATTTTGTTGTTGTAATCAATGCG GAAAAAATCGTATTGACCGGTAAAAAAATGGAGAACAAGAAATACTACCGTCACTCCCTG
MES17           CACAAGCCACAATTTACACCACATGTGGACACAGGTGATTTTGTTGTTGTAATCAATGCG GAAAAAATCGTATTGACCGGTAAAAAAATGGAGAACAAGAAATACTACCGTCACTCCCTG
MES108          CACAAGCCACAATTTACACCACATGTGGACACAGGTGATTTTGTTGTTGTAATCAATGCG GAAAAAATCGTATTGACCGGTAAAAAAATGGAGAACAAGAAATACTACCGTCACTCCCTG
YUPP_8          CACAAGCCACAATTTACACCACATGTGGACACAGGTGATTTTGTTGTTGTAATCAATGCG GAAAAAATCGTATTGACCGGTAAAAAAATGGAGAACAAGAAATACTACCGTCACTCCCTG
YC0136          CACAAGCCACAATTTACACCACATGTGGACACAGGTGATTTTGTTGTTGTAATCAATGCG GAAAAAATCGTATTGACCGGTAAAAAAATGGAGAACAAGAAATACTACCGTCACTCCCTG
P3              CACAAGCCACAATTTACACCACATGTGGACACAGGTGATTTTGTTGTTGTAATCAATGCG GAAAAAATCGTATTGACCGGTAAAAAAATGGAGAACAAGAAATACTACCGTCACTCCCTG
CCI_25          CACAAGCCACAATTTACACCACATGTGGACACAGGTGATTTTGTTGTTGTAATCAATGCG GAAAAAATCGTATTGACCGGTAAAAAAATGGAGAACAAGAAATACTACCGTCACTCCCTG
ND24            CACAAGCCACAATTTACACCACATGTGGACACAGGTGATTTTGTTATTGTAATCAATGCG GAGAAAATCGTATTGACCGGTAAAAAAATGGAGAACAAGAAATACTATCGTCACTCCATG
ZF197           CATAAGCCACAATTTACACCACATGTGGACACAGGTGATTTTGTTATTGTAATCAATGCG GAGAAAATCGTATTGACCGGTAAAAAAATGGAGAACAAGAAATACTATCGTCACTCCATG
P.ottowii       CATAAGCCACAATTTACACCACATGTGGACACAGGTGATTTTGTTATTGTAATCAATGCG GAGAAAATCGTATTGACCGGTAAAAAAATGGAGAACAAGAAATACTATCGTCACTCCATG
181MFCol51      CACAAGCCACAGTTTACACCACATGTGGACACAGGTGATTTTGTTGTTGTAATCAATGCG GAGAAAATCGTATTGACCGGTAAAAAAATGGAGAACAAAAAATACTATCGTCACTCCCTG
SQR_21          CACAAGCCACAGTTTACACCACATGTGGACACAGGTGATTTTGTTGTTGTAATCAATGCG GAGAAAATCGTATTGACCGGTAAAAAAATGGAGAACAAAAAATACTATCGTCACTCCCTG
CF05            CACAAGCCACAGTTTACACCACATGTGGACACAGGTGATTTTGTTGTTGTAATCAATGCG GAGAAAATCGTATTGACCGGTAAAAAAATGGAGAACAAAAAATACTATCGTCACTCCCTG
CICC_10580      CACAAGCCACAGTTTACACCACATGTGGACACAGGTGATTTTGTTGTTGTAATCAATGCG GAGAAAATCGTATTGACCGGTAAAAAAATGGAGAACAAAAAATACTATCGTCACTCCCTG
JE201           CACAAGCCACAGTTTACACCACATGTGGACACAGGTGATTTTGTTGTTGTAATCAATGCG GAGAAAATCGTATTGACCGGTAAAAAAATGGAGAACAAAAAATACTATCGTCACTCCCTG
ATCC_842        CACAAGCCACAGTTTACACCACATGTGGACACAGGTGATTTTGTTGTTGTAATCAATGCG GAGAAAATCGTATTGACCGGTAAAAAAATGGAGAACAAAAAATACTATCGTCACTCCCTG
S3              CACAAGCCACAGTTTACACCACATGTGGACACAGGTGATTTTGTTGTTGTAATCAATGCG GAGAAAATCGTATTGACCGGTAAAAAAATGGAGAACAAAAAATACTATCGTCACTCCCTG
OSY_DF          CACAAGCCACAGTTTACACCACATGTGGATACAGGTGATTTTGTTGTTGTAATCAATGCG GAGAAAATCGTATTGACCGGTAAAAAAATGGAGAACAAAAAATACTATCGTCACTCCCTG
OSY_EC          CACAAGCCACAGTTTACACCACATGTGGATACAGGTGATTTTGTTGTTGTAATCAATGCG GAGAAAATCGTATTGACCGGTAAAAAAATGGAGAACAAAAAATACTATCGTCACTCCCTG
C12             CACAAGCCACAGTTTACACCACATGTGGATACAGGTGATTTTGTTGTTGTAATCAATGCG GAGAAAATCGTATTGACCGGTAAAAAAATGGAGAACAAAAAATACTATCGTCACTCCCTG
NRRL_B_30509    CACAAGCCACAGTTTACACCACATGTGGACACAGGTGATTTTGTTGTTGTAATCAATGCG GAGAAAATCGTATTGACCGGTAAAAAAATGGAGAACAAAAAATACTATCGTCACTCCCTG
NCTC7575        CACAAGCCACAGTTTACACCACATGTGGACACAGGTGATTTTGTTGTTGTAATCAATGCG GAGAAAATCGTATTGACCGGTAAAAAAATGGAGAACAAAAAATACTATCGTCACTCCCTG
MEZ6            CACAAGCCACAGTTTACACCACATGTGGACACAGGTGATTTTGTTGTTGTAATCAATGCG GAGAAAATCGTATTGACCGGTAAAAAAATGGAGAACAAAAAATACTATCGTCACTCCCTG
O_211           CACAAGCCACAGTTTACACCACATGTGGACACAGGTGATTTTGTTGTTGTAATCAATGCG GAGAAAATCGTATTGACCGGTAAAAAAATGGAGAACAAAAAATACTATCGTCACTCCCTG
HK4             CACAAGCCACAGTTTACACCACATGTGGACACAGGTGATTTTGTTGTTGTAATCAATGCG GAGAAAATCGTATTGACCGGTAAAAAAATGGAGAACAAAAAATACTATCGTCACTCCCTG
A18             CACAAGCCACAGTTTACACCACATGTGGACACAGGTGATTTTGTTGTTGTAATCAATGCG GAGAAAATCGTATTGACCGGTAAAAAAATGGAGAACAAAAAATACTATCGTCACTCCCTG
TH2H2           CACAAGCCACAGTTTACACCACATGTGGACACAGGTGATTTTGTTGTTGTAATCAATGCG GAGAAAATCGTATTGACCGGTAAAAAAATGGAGAACAAAAAATACTATCGTCACTCCCTG
EB4_G3          CACAAGCCACAGTTTACACCACATGTGGACACAGGTGATTTTGTTGTTGTAATCAATGCG GAGAAAATCGTATTGACCGGTAAAAAAATGGAGAACAAAAAATACTATCGTCACTCCCTG
DCB2            CACAAGCCACAGTTTACACCACATGTGGACACAGGTGATTTTGTTGTTGTAATCAATGCG GAGAAAATCGTATTGACCGGTAAAAAAATGGAGAACAAAAAATACTATCGTCACTCCCTG
ZF129           CACAAGCCACAGTTTACACCACATGTGGACACAGGTGATTTTGTTGTTGTAATCAATGCG GAGAAAATCGTATTGACCGGTAAAAAAATGGAGAACAAAAAATACTATCGTCACTCCCTG
KP10            CACAAGCCACAGTTTACACCACATGTGGACACAGGTGATTTTGTTGTTGTAATCAATGCG GAGAAAATCGTATTGACCGGTAAAAAAATGGAGAACAAAAAATACTATCGTCACTCCCTG
IIF8SW          CACAAGCCACAGTTTACACCACATGTGGACACAGGTGATTTTGTTGTTGTAATCAATGCG GAGAAAATCGTATTGACCGGTAAAAAAATGGAGAACAAAAAATACTATCGTCACTCCCTG
2A_2A           CACAAGCCACAGTTTACACCACATGTGGACACAGGTGATTTTGTTGTTGTAATCAATGCG GAGAAAATCGTATTGACCGGTAAAAAAATGGAGAACAAAAAATACTATCGTCACTCCCTG
YS18            CACAAGCCACAGTTTACACCACATGTGGACACAGGTGATTTTGTTGTTGTAATCAATGCG GAGAAAATCGTATTGACCGGTAAAAAAATGGAGAACAAAAAATACTATCGTCACTCCCTG
EBL06           CACAAGCCACAGTTTACACCACATGTGGACACAGGTGATTTTGTTGTTGTAATCAATGCG GAGAAAATCGTATTGACCGGTAAAAAAATGGAGAACAAAAAATACTATCGTCACTCCCTG
HY96            CACAAGCCACAGTTTACACCACATGTGGACACAGGTGATTTTGTTGTTGTAATCAATGCG GAGAAAATCGTATTGACCGGTAAAAAAATGGAGAACAAAAAATACTATCGTCACTCCCTG
ZJ_9            CACAAGCCACAGTTTACACCACATGTGGACACAGGTGATTTTGTTGTTGTAATCAATGCG GAGAAAATCGTATTGACCGGTAAAAAAATGGAGAACAAAAAATACTATCGTCACTCCCTG
KCCM_40454      CACAAGCCACAGTTTACACCACATGTGGACACAGGTGATTTTGTTGTTGTAATCAATGCG GAGAAAATCGTATTGACCGGTAAAAAAATGGAGAACAAAAAATACTATCGTCACTCCCTG
Sb3             CACAAGCCACAGTTTACACCACATGTGGACACAGGTGATTTTGTTGTTGTAATCAATGCG GAGAAAATCGTATTGACCGGTAAAAAAATGGAGAACAAGAAATACTATCGTCACTCCCTG
CJX518          CACAAGCCACAGTTTACACCACATGTGGACACAGGTGATTTTGTTGTTGTAATCAATGCG GAGAAAATCGTATTGACCGGTAAAAAAATGGAGAACAAGAAATACTATCGTCACTCCCTG
KF_1            CACAAGCCACAGTTTACACCACATGTGGACACAGGTGATTTTGTTGTTGTAATCAATGCG GAGAAAATCGTATTGACCGGTAAAAAAATGGAGAACAAGAAATACTATCGTCACTCCCTG
ND25            CACAAGCCACAGTTTACACCACATGTGGACACAGGTGATTTTGTTGTTGTAATCAATGCG GAGAAAATCGTATTGACCGGTAAAAAAATGGAGAACAAGAAATACTATCGTCACTCCCTG
1_43            CACAAGCCACAGTTTACACCACATGTGGACACAGGTGATTTTGTTGTTGTAATCAATGCG GAGAAAATCGTATTGACCGGTAAAAAAATGGAGAACAAGAAATACTATCGTCACTCCCTG
SC2             CACAAGCCACAGTTTACACCACATGTGGACACAGGTGATTTTGTTGTTGTAATCAATGCG GAGAAAATCGTATTGACCGGTAAAAAAATGGAGAACAAGAAATACTATCGTCACTCCCTG
M1              CACAAGCCACAGTTTACACCACATGTGGACACAGGTGATTTTGTTGTTGTAATCAATGCG GAGAAAATCGTATTGACCGGTAAAAAAATGGAGAACAAGAAATACTATCGTCACTCCCTG
                ** ******** **************** *************** ************** ** ***************** ***************** ******** ********* **
```

Cluster 1 CAC AAGCCACA$^{A/G}$TTTACACCACATGTGGACAC AGGTGATTTTGTTGTTGTAATCAATGCGGAAAAAATCGTATTGACCGG$^{A/T}$AAAAAAATGGAGAACAAGAAATACTA$^{T/C}$CGTCACTCCCTG
Cluster 2 CA$^{C/T}$AAGCCACAA TTTACACCACATGTGGACAC AGGTGATTTTGTTATTGTAATCAATGCGGAGAAAATCGTATTGACCGGT AAAAAAATGGAGAACAAGAAATACTAT CGTCACTCCATG
Cluster 3 CAC AAGCCACAG TTTACACCACATGTGGACAC AGGTGATTTTGTTGTTGTAATCAATGCGGAGAAAATCGTATTGACCGGT AAAAAAATGGAGAACAAGAAATACTAT CGTCACTCCCTG
Cluster 4 CAC AAGCCACAG TTTACACCACATGTGGACA$^{C/T}$AGGTGATTTTGTTGTTGTAATCAATGCGGAGAAAATCGTATTGACCGGT AAAAAAATGGAGAACAAAAAATACTAT CGTCACTCCCTG

```
MES972         CATCCGGGTGGTCTGAAAGTAACGGTTGCACAAGAAATGGTTAAGAACAAGCCTGAGCGT ATGTTGGAGTTGGCTGTTCACGGTATGCTTCCTAAAACTCGCCAAGGCGATAAAATGAAG
DSM_365        CATCCGGGTGGTCTGAAAGTAACGGTTGCACAAGAAATGGTTAAGAACAAGCCTGAGCGC ATGTTGGAATTGGCTGTTCACGGTATGCTTCCTAAAACTCGTCAAGGCGAGAAAATGAAG
WLY78          CATCCGGGTGGTCTGAAAGTAACGGTTGCACAAGAAATGGTTAAGAACAAGCCTGAGCGC ATGTTGGAATTGGCTGTTCACGGTATGCTTCCTAAAACTCGTCAAGGCGAGAAAATGAAG
MES110         CATCCGGGTGGTCTGAAAGTAACGGTTGCACAAGAAATGGTTAAGAACAAGCCTGAGCGC ATGTTGGAATTGGCTGTTCACGGTATGCTTCCTAAAACTCGTCAAGGCGAGAAAATGAAG
CFSAN034341    CATCCGGGTGGTCTGAAAGTAACGGTTGCACAAGAAATGGTTAAGAACAAGCCTGAGCGC ATGTTGGAATTGGCTGTTCACGGTATGCTTCCTAAAACTCGTCAAGGCGAGAAAATGAAG
Hob6           CATCCGGGTGGTCTGAAAGTAACGGTTGCACAAGAAATGGTTAAGAACAAGCCTGAGCGC ATGTTGGAATTGGCTGTTCACGGTATGCTTCCTAAAACTCGTCAAGGCGAGAAAATGAAG
LMG_27872      CATCCGGGTGGTCTGAAAGTAACGGTTGCACAAGAAATGGTTAAGAACAAGCCTGAGCGC ATGTTGGAATTGGCTGTTCACGGTATGCTTCCTAAAACTCGTCAAGGCGAGAAAATGAAG
E681           CATCCGGGTGGTCTGAAAGTAACGGTTGCACAAGAAATGGTTAAGAACAAGCCTGAGCGT ATGTTGGAATTGGCTGTTCATGGTATGCTTCCTAAAACTCGTCAAGGCGAGAAAATGAAG
J              CATCCGGGTGGTCTGAAAGTAACGGTTGCACAAGAAATGGTTAAGAACAAGCCTGAGCGT ATGTTAGAATTGGCTGTTCACGGTATGCTTCCTAAAACTCGTCAAGGCGAGAAAATGAAG
YC0573         CATCCGGGTGGTCTGAAAGTAACGGTTGCACAAGAAATGGTTAAGAACAAGCCTGAGCGT ATGTTGGAATTGGCTGTTCACGGTATGCTTCCTAAAACTCGTCAAGGCGAGAAAATGAAG
LY214          CATCCGGGTGGTCTGAAAGTAACGGTTGCACAAGAAATGGTTAAGAACAAGCCTGAGCGT ATGTTGGAATTGGCTGTTCACGGTATGCTTCCTAAAACTCGTCAAGGCGAGAAAATGAAG
CR1            CATCCGGGTGGTCTGAAAGTAACGGTTGCACAAGAAATGGTTAAGAACAAGCCTGAGCGT ATGTTGGAATTGGCTGTTCACGGTATGCTTCCTAAAACTCGTCAAGGCGAGAAAATGAAG
K16            CATCCGGGTGGTCTGAAAGTAACGGTTGCACAAGAAATGGTTAAGAACAAGCCTGAGCGT ATGTTGGAATTGGCTGTTCACGGTATGCTTCCTAAAACTCGTCAAGGCGAGAAAATGAAG
CFSAN034343    CATCCGGGTGGTCTGAAAGTAACGGTTGCACAAGAAATGGTTAAGAACAAGCCTGAGCGT ATGTTGGAATTGGCTGTTCACGGTATGCTTCCTAAAACTCGTCAAGGCGAGAAAATGAAG
CFSAN034342    CATCCGGGTGGTCTGAAAGTAACGGTTGCACAAGAAATGGTTAAGAACAAGCCTGAGCGT ATGTTGGAATTGGCTGTTCACGGTATGCTTCCTAAAACTCGTCAAGGCGAGAAAATGAAG
ATCC_15970     CATCCGGGTGGTCTGAAAGTAACGGTTGCACAAGAAATGGTTAAGAACAAGCCTGAGCGT ATGTTGGAATTGGCTGTTCACGGTATGCTTCCTAAAACTCGTCAAGGCGAGAAAATGAAG
DSM_292        CATCCGGGTGGTCTGAAAGTAACGGTTGCACAAGAAATGGTTAAGAACAAGCCTGAGCGT ATGTTGGAATTGGCTGTTCACGGTATGCTTCCTAAAACTCGTCAAGGCGAGAAAATGAAG
MES20          CATCCGGGTGGTCTGAAAGTAACGGTTGCACAAGAAATGGTTAAGAACAAGCCTGAGCGT ATGTTGGAATTGGCTGTTCACGGTATGCTTCCTAAAACTCGTCAAGGCGAGAAAATGAAG
MES17          CATCCGGGTGGTCTGAAAGTAACGGTTGCACAAGAAATGGTTAAGAACAAGCCTGAGCGT ATGTTGGAATTGGCTGTTCACGGTATGCTTCCTAAAACTCGTCAAGGCGAGAAAATGAAG
MES108         CATCCGGGTGGTCTGAAAGTAACGGTTGCACAAGAAATGGTTAAGAACAAGCCTGAGCGT ATGTTGGAATTGGCTGTTCACGGTATGCTTCCTAAAACTCGTCAAGGCGAGAAAATGAAG
YUPP_8         CATCCGGGTGGTCTGAAAGTAACGGTTGCACAAGAAATGGTTAAGAACAAGCCTGAGCGT ATGTTGGAATTGGCTGTTCACGGTATGCTTCCTAAAACTCGTCAAGGCGAGAAAATGAAG
YC0136         CATCCGGGTGGTCTGAAAGTAACGGTTGCACAAGAAATGGTTAAGAACAAGCCTGAGCGT ATGTTGGAATTGGCTGTTCACGGTATGCTTCCTAAAACTCGTCAAGGCGAGAAAATGAAG
P3             CATCCGGGTGGTCTGAAAGTAACGGTTGCACAAGAAATGGTTAAGAACAAGCCTGAGCGT ATGTTGGAATTGGCTGTTCACGGTATGCTTCCTAAAACTCGTCAAGGCGAGAAAATGAAG
CCI_25         CATCCGGGTGGTCTGAAAGTAACGGTTGCACAAGAAATGGTTAAGAACAAGCCTGAGCGC ATGTTGGAATTGGCTGTTCACGGTATGCTTCCTAAAACTCGTCAAGGCGAGAAAATGAAG
ND24           CACCCAGGTGGTCTGAAAGTAACAGTTGCACAAGATATGGTTAAGAACAAACCTGAACGT ATGTTGGAATTGGCTGTTCACGGTATGCTTCCTAAAACTCGTCAAGGCGAAAAAATGAAG
ZF197          CACCCAGGTGGTCTGAAAGTAACAGTTGCACAAGATATGGTTAAGAACAAACCTGAACGT ATGTTGGAATTGGCTGTTCACGGTATGCTTCCTAAAACTCGTCAAGGCGACAAAATGAAG
P.ottowii      CACCCAGGTGGTCTGAAAGTAACAGTTGCACAAGATATGGTTAAGAACAAACCTGAACGT ATGTTGGAATTGGCTGTTCACGGTATGCTTCCTAAAACTCGTCAAGGCGAAAAAATGAAG
181MFCol51     CACCCAGGTGGTCTGAAAGTAACAGTTGCACAAGATATGGTTAAGAACAAACCTGAACGT ATGTTGGAATTGGCTGTTCACGGTATGCTTCCTAAAACTCGTCAAGGTGACAAAATGAAG
SQR_21         CACCCAGGTGGTCTGAAAGTAACAGTTGCACAAGATATGGTTAAGAACAAACCTGAACGT ATGTTGGAATTGGCTGTTCACGGTATGCTTCCTAAAACTCGTCAAGGTGACAAAATGAAG
CF05           CACCCAGGTGGTCTGAAAGTAACAGTTGCACAAGATATGGTTAAGAACAAACCTGAACGT ATGTTGGAATTGGCTGTTCACGGTATGCTTCCTAAAACTCGTCAAGGTGACAAAATGAAG
CICC_10580     CACCCAGGTGGTCTGAAAGTAACAGTTGCACAAGATATGGTTAAGAACAAACCTGAACGT ATGTTGGAATTGGCTGTTCACGGTATGCTTCCTAAAACTCGTCAAGGCGACAAAATGAAG
JE201          CACCCAGGTGGTCTGAAAGTAACAGTTGCACAAGATATGGTTAAGAACAAACCTGAACGT ATGTTGGAATTGGCTGTTCACGGTATGCTTCCTAAAACTCGTCAAGGCGACAAAATGAAG
ATCC_842       CACCCAGGTGGTCTGAAAGTAACAGTTGCACAAGATATGGTTAAGAACAAACCTGAACGT ATGTTGGAATTGGCTGTTCACGGTATGCTTCCTAAAACTCGTCAAGGCGACAAAATGAAG
S3             CACCCAGGTGGTCTGAAAGTAACAGTTGCACAAGATATGGTTAAGAACAAACCTGAACGT ATGTTGGAATTGGCTGTTCACGGTATGCTTCCTAAAACTCGTCAAGGCGACAAAATGAAG
OSY_DF         CACCCAGGTGGTCTGAAAGTAACAGTTGCACAAGATATGGTTAAGAACAAACCTGAACGT ATGTTGGAATTGGCTGTTCACGGTATGCTTCCTAAAACTCGTCAAGGCGACAAAATGAAG
OSY_EC         CACCCAGGTGGTCTGAAAGTAACAGTTGCACAAGATATGGTTAAGAACAAACCTGAACGT ATGTTGGAATTGGCTGTTCACGGTATGCTTCCTAAAACTCGTCAAGGCGACAAAATGAAG
C12            CACCCAGGTGGTCTGAAAGTAACAGTTGCACAAGATATGGTTAAGAACAAACCTGAACGT ATGTTGGAATTGGCTGTTCACGGTATGCTTCCTAAAACTCGTCAAGGCGACAAAATGAAG
NRRL_B_30509   CACCCAGGTGGTCTGAAAGTAACAGTTGCACAAGATATGGTTAAGAACAAACCTGAACGT ATGTTGGAATTGGCTGTTCACGGTATGCTTCCTAAAACTCGTCAAGGCGACAAAATGAAG
NCTC7575       CACCCAGGTGGTCTGAAAGTAACAGTTGCACAAGATATGGTTAAGAACAAACCTGAACGT ATGTTGGAATTGGCTGTTCACGGTATGCTTCCTAAAACTCGTCAAGGCGACAAAATGAAG
MEZ6           CACCCAGGTGGTCTGAAAGTAACAGTTGCACAAGATATGGTTAAGAACAAACCTGAACGT ATGTTGGAATTGGCTGTTCACGGTATGCTTCCTAAAACTCGTCAAGGCGACAAAATGAAG
O_211          CACCCAGGTGGTCTGAAAGTAACAGTTGCACAAGATATGGTTAAGAACAAACCTGAACGT ATGTTGGAATTGGCTGTTCACGGTATGCTTCCTAAAACTCGTCAAGGCGACAAAATGAAG
HK4            CACCCAGGTGGTCTGAAAGTAACAGTTGCACAAGATATGGTTAAGAACAAACCTGAACGT ATGTTGGAATTGGCTGTTCACGGTATGCTTCCTAAAACTCGTCAAGGCGACAAAATGAAG
A18            CACCCAGGTGGTCTGAAAGTAACAGTTGCACAAGATATGGTTAAGAACAAACCTGAACGT ATGTTGGAATTGGCTGTTCACGGTATGCTTCCTAAAACTCGTCAAGGCGACAAAATGAAG
TH2H2          CACCCAGGTGGTCTGAAAGTAACAGTTGCACAAGATATGGTTAAGAACAAACCTGAACGT ATGTTGGAATTGGCTGTTCACGGTATGCTTCCTAAAACTCGTCAAGGCGACAAAATGAAG
EB4_G3         CACCCAGGTGGTCTGAAAGTAACAGTTGCACAAGATATGGTTAAGAACAAACCTGAACGT ATGTTGGAATTGGCTGTTCACGGTATGCTTCCTAAAACTCGTCAAGGCGACAAAATGAAG
DCB2           CACCCAGGTGGTCTGAAAGTAACAGTTGCACAAGATATGGTTAAGAACAAACCTGAACGT ATGTTGGAATTGGCTGTTCACGGTATGCTTCCTAAAACTCGTCAAGGCGACAAAATGAAG
ZF129          CACCCAGGTGGTCTGAAAGTAACAGTTGCACAAGATATGGTTAAGAACAAACCTGAACGT ATGTTGGAATTGGCTGTTCACGGTATGCTTCCTAAAACTCGTCAAGGCGACAAAATGAAG
KP10           CACCCAGGTGGTCTGAAAGTAACAGTTGCACAAGATATGGTTAAGAACAAACCTGAACGT ATGTTGGAATTGGCTGTTCACGGTATGCTTCCTAAAACTCGTCAAGGCGACAAAATGAAG
IIF8SW         CACCCAGGTGGTCTGAAAGTAACAGTTGCACAAGATATGGTTAAGAACAAACCTGAACGT ATGTTGGAATTGGCTGTTCACGGTATGCTTCCTAAAACTCGTCAAGGCGACAAAATGAAG
2A_2A          CACCCAGGTGGTCTGAAAGTAACAGTTGCACAAGATATGGTTAAGAACAAACCTGAACGT ATGTTGGAATTGGCTGTTCACGGTATGCTTCCTAAAACTCGTCAAGGCGACAAAATGAAG
YS18           CACCCAGGTGGTCTGAAAGTAACAGTTGCACAAGATATGGTTAAGAACAAACCTGAACGT ATGTTGGAATTGGCTGTTCACGGTATGCTTCCTAAAACTCGTCAAGGCGACAAAATGAAG
EBL06          CACCCAGGTGGTCTGAAAGTAACAGTTGCACAAGATATGGTTAAGAACAAACCTGAACGT ATGTTGGAATTGGCTGTTCACGGTATGCTTCCTAAAACTCGTCAAGGCGACAAAATGAAG
HY96           CACCCAGGTGGTCTGAAAGTAACAGTTGCACAAGATATGGTTAAGAACAAACCTGAACGT ATGTTGGAATTGGCTGTTCACGGTATGCTTCCTAAAACTCGTCAAGGCGACAAAATGAAG
ZJ_9           CACCCAGGTGGTCTGAAAGTAACAGTTGCACAAGATATGGTTAAGAACAAACCTGAACGT ATGTTGGAATTGGCTGTTCACGGTATGCTTCCTAAAACTCGTCAAGGCGACAAAATGAAG
KCCM_40454     CACCCAGGTGGTCTGAAAGTAACAGTTGCACAAGATATGGTTAAGAACAAACCTGAACGT ATGTTGGAATTGGCTGTTCACGGTATGCTTCCTAAAACTCGTCAAGGCGACAAAATGAAG
Sb3            CACCCAGGTGGTCTGAAAGTAACAGTTGCACAAGATATGGTTAAGAACAAACCTGAACGT ATGTTGGAATTGGCTGTTCACGGTATGCTTCCTAAAACTCGTCAAGGCGACAAAATGAAG
CJX518         CACCCAGGTGGTCTGAAAGTAACAGTTGCACAAGATATGGTTAAGAACAAACCTGAACGT ATGTTGGAATTGGCTGTTCACGGTATGCTTCCTAAAACTCGTCAAGGCGACAAAATGAAG
KF_1           CACCCAGGTGGTCTGAAAGTAACAGTTGCACAAGATATGGTTAAGAACAAACCTGAACGT ATGTTGGAATTGGCTGTTCACGGTATGCTTCCTAAAACTCGTCAAGGCGACAAAATGAAG
ND25           CACCCAGGTGGTCTGAAAGTAACAGTTGCACAAGATATGGTTAAGAACAAACCTGAACGT ATGTTGGAATTGGCTGTTCACGGTATGCTTCCTAAAACTCGTCAAGGCGACAAAATGAAG
1_43           CACCCAGGTGGTCTGAAAGTAACAGTTGCACAAGATATGGTTAAGAACAAACCTGAACGT ATGTTGGAATTGGCTGTTCACGGTATGCTTCCTAAAACTCGTCAAGGCGACAAAATGAAG
SC2            CACCCAGGTGGTCTGAAAGTAACAGTTGCACAAGATATGGTTAAGAACAAACCTGAACGT ATGTTGGAATTGGCTGTTCACGGTATGCTTCCTAAAACTCGTCAAGGCGACAAAATGAAG
M1             CACCCAGGTGGTCTGAAAGTAACAGTTGCACAAGATATGGTTAAGAACAAACCTGAACGT ATGTTGGAATTGGCTGTTCACGGTATGCTTCCTAAAACTCGTCAAGGCGACAAAATGAAG
               ** ** ***************** *********** ************** ***** ** ***** ** *********** ******************** ***** ** *********
```

```
Cluster 1      CATCCGGGTGGTCTGAAAGTAACGGTTGCACAAGAAATGGTTAAGAACAAGCCTGAGCGT/CATGTTG/AGAA/GTTGGCTGTTCAC/TGGTATGCTTCCTAAAACTCGT/CCAAGGC
Cluster 2,3    CACCCAGGTGGTCTGAAAGTAACAGTTGCACAAGATATGGTTAAGAACAAACCTGAACGT ATGTTG GAA TTGGCTGTTCAC GGTATGCTTCCTAAAACTCGT CAAGGC
Cluster 4      CACCCAGGTGGTCTGAAAGTAACAGTTGCACAAGATATGGTTAAGAACAAACCTGAACGT ATGTTG GAA TTGGCTGTTCAC GGTATGCTTCCTAAAACTCGT CAAGGC/T
```

```
MES972       CTGAGACTTAAAGCTTACAGAGGCACTGAGCATCCACATGCAGCACAAAAACCTGAAGTT TACGAACTTCGCGGATAA
DSM_365      CTGAGACTTAAAGCTTACAGAGGCACTGAGCATCCACATGTAGCACAAAAACCTGAAGTT TACGAACTTCGCGGATAA
WLY78        CTGAGACTTAAAGCTTACAGAGGCACTGAGCATCCACATGCAGCACAAAAACCTGAAGTT TACGAACTTCGCGGATAA
MES110       CTGAGACTTAAAGCTTACAGAGGCACTGAGCATCCACATGCAGCACAAAAACCTGAAGTT TACGAACTTCGCGGATAA
CFSAN034341  CTGAGACTTAAAGCTTACAGAGGCACTGAGCATCCACATGCAGCACAAAAACCTGAAGTT TACGAACTTCGCGGATAA
Hob6         CTGAGACTTAAAGCTTACAGAGGCACTGAGCATCCACATGCAGCACAAAAACCTGAAGTT TACGAACTTCGCGGATAA
LMG_27872    CTGAGACTTAAAGCTTACAGAGGCACTGAGCATCCACATGCAGCACAAAAACCTGAAGTT TACGAACTTCGCGGATAA
E681         CTGAGACTTAAAGCTTACAGAGGCACTGAGCATCCACATGCAGCACAAAAACCTGAAGTT TACGAACTTCGCGGATAA
J            CTGAGACTTAAAGCTTACAGAGGCACTGAGCATCCACATGCAGCACAAAAACCTGAAGTT TACGAACTTCGCGGATAA
YC0573       CTGAGACTTAAAGCTTACAGAGGCACTGAGCATCCACATGCAGCACAAAAACCTGAAGTT TACGAACTTCGCGGATAA
LY214        CTGAGACTTAAAGCTTACAGAGGCACTGAGCATCCACATGCAGCACAAAAACCTGAAGTT TACGAACTTCGCGGATAA
CR1          CTGAGACTTAAAGCTTACAGAGGCACTGAGCATCCACATGCAGCACAAAAACCTGAAGTT TACGAACTTCGCGGATAA
K16          CTGAGACTTAAAGCTTACAGAGGCACTGAGCATCCACATGCAGCACAAAAACCTGAAGTT TACGAACTTCGCGGATAA
CFSAN034343  CTGAGACTTAAAGCTTACAGAGGCACTGAGCATCCACATGCAGCACAAAAACCTGAAGTT TACGAACTTCGCGGATAA
CFSAN034342  CTGAGACTTAAAGCTTACAGAGGCACTGAGCATCCACATGCAGCACAAAAACCTGAAGTT TACGAACTTCGCGGATAA
ATCC_15970   CTGAGACTTAAAGCTTACAGAGGCACTGAGCATCCACATGCAGCACAAAAACCTGAAGTT TACGAACTTCGCGGATAA
DSM_292      CTGAGACTTAAAGCTTACAGAGGCACTGAGCATCCACATGCAGCACAAAAACCTGAAGTT TACGAACTTCGCGGATAA
MES20        CTGAGACTTAAAGCTTACAGAGGCACTGAGCATCCACATGCAGCACAAAAACCTGAAGTT TACGAACTTCGCGGATAA
MES17        CTGAGACTTAAAGCTTACAGAGGCACTGAGCATCCACATGCAGCACAAAAACCTGAAGTT TACGAACTTCGCGGATAA
MES108       CTGAGACTTAAAGCTTACAGAGGCACTGAGCATCCACATGCAGCACAAAAACCTGAAGTT TACGAACTTCGCGGATAA
YUPP_8       CTGAGACTTAAAGCTTACAGAGGCACTGAGCATCCACATGCAGCACAAAAACCTGAAGTT TACGAACTTCGCGGATAA
YC0136       CTGAGACTTAAAGCTTACAGAGGCACTGAGCATCCACATGCAGCACAAAAACCTGAAGTT TACGAACTTCGCGGATAA
P3           CTGAGACTTAAAGCTTACAGAGGCACTGAGCATCCACATGCAGCACAAAAACCTGAAGTT TACGAACTTCGCGGATAA
CCI_25       CTGAGACTTAAAGCTTACAGAGGCACTGAGCATCCACATGCAGCACAAAAACCTGAAGTT TACGAACTTCGCGGATAA
ND24         CTGAGACTTAAAGCTTACAGAGGTACAGAGCATCCACATGCAGCACAAAAACCTGAAGTT TACGAACTTCGCGGATAA
ZF197        CTGAGACTTAAAGCTTACAGAGGTACAGAGCATCCACATGCAGCACAAAAACCTGAAGTT TACGAACTTCGCGGATAA
P.ottowii    CTGAGACTTAAAGCTTACAGAGGTACAGAGCATCCACATGCAGCACAAAAACCTGAAGTT TACGAACTTCGCGGATAA
181MFCol51   CTGAGACTTAAAGCTTACAGAGGTACAGAGCATCCACATGCAGCACAAAAACCTGAAGTT TACGAACTTCGCGGATAA
SQR_21       CTGAGACTTAAAGCTTACAGAGGTACAGAGCATCCACATGCAGCACAAAAACCTGAAGTT TACGAACTTCGCGGATAA
CF05         CTGAGACTTAAAGCTTACAGAGGTACAGAGCATCCACATGCAGCACAAAAACCTGAAGTT TACGAACTTCGCGGATAA
CICC_10580   CTGAGACTTAAAGCTTACAGAGGTACAGAGCATCCACATGCAGCACAAAAACCTGAAGTT TACGAACTTCGCGGATAA
JE201        CTGAGACTTAAAGCTTACAGAGGTACAGAGCATCCACATGCAGCACAAAAACCTGAAGTT TACGAACTTCGCGGATAA
ATCC_842     CTGAGACTTAAAGCTTACAGAGGTACAGAGCATCCACATGCAGCACAAAAACCTGAAGTT TACGAACTTCGCGGATAA
S3           CTGAGACTTAAAGCTTACAGAGGTACAGAGCATCCACATGCAGCACAAAAACCTGAAGTT TACGAACTTCGCGGATAA
OSY_DF       CTGAGACTTAAAGCTTACAGAGGTACAGAGCATCCACATGCAGCACAAAAACCTGAAGTT TACGAACTTCGCGGATAA
OSY_EC       CTGAGACTTAAAGCTTACAGAGGTACAGAGCATCCACATGCAGCACAAAAACCTGAAGTT TACGAACTTCGCGGATAA
C12          CTGAGACTTAAAGCTTACAGAGGTACAGAGCATCCACATGCAGCACAAAAACCTGAAGTT TACGAACTTCGCGGATAA
NRRL_B_30509 CTGAGACTTAAAGCTTACAGAGGTACAGAGCATCCACATGCAGCACAAAAACCTGAAGTT TACGAACTTCGCGGATAA
NCTC7575     CTGAGACTTAAAGCTTACAGAGGTACAGAGCATCCACATGCAGCACAAAAACCTGAAGTT TACGAACTTCGCGGATAA
MEZ6         CTGAGACTTAAAGCTTACAGAGGTACAGAGCATCCACATGCAGCACAAAAACCTGAAGTT TACGAACTTCGCGGATAA
O_211        CTGAGACTTAAAGCTTACAGAGGTACAGAGCATCCACATGCAGCACAAAAACCTGAAGTT TACGAACTTCGCGGATAA
HK4          CTGAGACTTAAAGCTTACAGAGGTACAGAGCATCCACATGCAGCACAAAAACCTGAAGTT TACGAACTTCGCGGATAA
A18          CTGAGACTTAAAGCTTACAGAGGTACAGAGCATCCACATGCAGCACAAAAACCTGAAGTT TACGAACTTCGCGGATAA
TH2H2        CTGAGACTTAAAGCTTACAGAGGTACAGAGCATCCACATGCAGCACAAAAACCTGAAGTT TACGAACTTCGCGGATAA
EB4_G3       CTGAGACTTAAAGCTTACAGAGGTACAGAGCATCCACATGCAGCACAAAAACCTGAAGTT TACGAACTTCGCGGATAA
DCB2         CTGAGACTTAAAGCTTACAGAGGTACAGAGCATCCACATGCAGCACAAAAACCTGAAGTT TACGAACTTCGCGGATAA
ZF129        CTGAGACTTAAAGCTTACAGAGGTACAGAGCATCCACATGCAGCACAAAAACCTGAAGTT TACGAACTTCGCGGATAA
KP10         CTGAGACTTAAAGCTTACAGAGGTACAGAGCATCCACATGCAGCACAAAAACCTGAAGTT TACGAACTTCGCGGATAA
IIF8SW       CTGAGACTTAAAGCTTACAGAGGTACAGAGCATCCACATGCAGCACAAAAACCTGAAGTT TACGAACTTCGCGGATAA
2A_2A        CTGAGACTTAAAGCTTACAGAGGTACAGAGCATCCACATGCAGCACAAAAACCTGAAGTT TACGAACTTCGCGGATAA
YS18         CTGAGACTTAAAGCTTACAGAGGTACAGAGCATCCACATGCAGCACAAAAACCTGAAGTT TACGAACTTCGCGGATAA
EBL06        CTGAGACTTAAAGCTTACAGAGGTACAGAGCATCCACATGCAGCACAAAAACCTGAAGTT TACGAACTTCGCGGATAA
HY96         CTGAGACTTAAAGCTTACAGAGGTACAGAGCATCCACATGCAGCACAAAAACCTGAAGTT TACGAACTTCGCGGATAA
ZJ_9         CTGAGACTTAAAGCTTACAGAGGTACAGAGCATCCACATGCAGCACAAAAACCTGAAGTT TACGAACTTCGCGGATAA
KCCM_40454   CTGAGACTTAAAGCTTACAGAGGTACAGAGCATCCACATGCAGCACAAAAACCTGAAGTT TACGAACTTCGCGGATAA
Sb3          CTGAGACTTAAAGCTTACAGAGGTACAGAACATCCACATGCAGCACAAAAACCTGAAGTT TACGAACTTCGCGGATAA
CJX518       CTGAGACTTAAAGCTTACAGAGGTACAGAACATCCACATGCAGCACAAAAACCTGAAGTT TACGAACTTCGCGGATAA
KF_1         CTGAGACTTAAAGCTTACAGAGGTACAGAGCATCCACATGCAGCACAAAAACCTGAAGTT TACGAACTTCGCGGATAA
ND25         CTGAGACTTAAAGCTTACAGAGGTACAGAGCATCCACATGCAGCACAAAAACCTGAAGTT TACGAACTTCGCGGATAA
1_43         CTGAGACTTAAAGCTTACAGAGGTACAGAGCATCCACATGCAGCACAAAAACCTGAAGTT TACGAACTTCGCGGATAA
SC2          CTGAGACTTAAAGCTTACAGAGGTACAGAGCATCCACATGCAGCACAAAAACCTGAAGTT TACGAACTTCGCGGATAA
M1           CTGAGACTTAAAGCTTACAGAGGTACAGAGCATCCACATGCAGCACAAAAACCTGAAGTT TACGAACTTCGCGGATAA
             *********************** ** ** ********** *******************  ******************
```

Figure S8. **Multiple alignment of the *rplM* gene.** The nucleotide sequences were obtained from the strains belonging to Cluster 1 (yellow), Cluster 2 (gray), Cluster 3 (green), and Cluster 4 (cyan). The red asterisks indicate conserved regions that can be used to design primers for gene amplification. The consensus sequences of each Cluster are displayed in the box below the alignment. Cluster-specific SNVs are highlighted using the same colors of the Clusters.

<u>Ribosomal protein L13</u>

```
OSY_DF       MRTTYMAKPNEVERNWHIIDAEGKTLGRLASEAAALIRGKHKPQFTPHVDTGDFVVVINA EKIVLTGKKMENKKYYRHSLHPGGLKVTVAQDMVKNKPERMLELAVHGMLPKTRQGDKMK
OSY_EC       MRTTYMAKPNEVERNWHIIDAEGKTLGRLASEAAALIRGKHKPQFTPHVDTGDFVVVINA EKIVLTGKKMENKKYYRHSLHPGGLKVTVAQDMVKNKPERMLELAVHGMLPKTRQGDKMK
ATCC_842     MRTTYMAKPNEVERNWHIIDAEGKTLGRLASEAAALIRGKHKPQFTPHVDTGDFVVVINA EKIVLTGKKMENKKYYRHSLHPGGLKVTVAQDMVKNKPERMLELAVHGMLPKTRQGDKMK
NRRL_B_30509 MRTTYMAKPNEVERNWHIIDAEGKTLGRLASEAAALIRGKHKPQFTPHVDTGDFVVVINA EKIVLTGKKMENKKYYRHSLHPGGLKVTVAQDMVKNKPERMLELAVHGMLPKTRQGDKMK
NCTC7575     MRTTYMAKPNEVERNWHIIDAEGKTLGRLASEAAALIRGKHKPQFTPHVDTGDFVVVINA EKIVLTGKKMENKKYYRHSLHPGGLKVTVAQDMVKNKPERMLELAVHGMLPKTRQGDKMK
MEZ6         MRTTYMAKPNEVERNWHIIDAEGKTLGRLASEAAALIRGKHKPQFTPHVDTGDFVVVINA EKIVLTGKKMENKKYYRHSLHPGGLKVTVAQDMVKNKPERMLELAVHGMLPKTRQGDKMK
O_211        MRTTYMAKPNEVERNWHIIDAEGKTLGRLASEAAALIRGKHKPQFTPHVDTGDFVVVINA EKIVLTGKKMENKKYYRHSLHPGGLKVTVAQDMVKNKPERMLELAVHGMLPKTRQGDKMK
HK4          MRTTYMAKPNEVERNWHIIDAEGKTLGRLASEAAALIRGKHKPQFTPHVDTGDFVVVINA EKIVLTGKKMENKKYYRHSLHPGGLKVTVAQDMVKNKPERMLELAVHGMLPKTRQGDKMK
A18          MRTTYMAKPNEVERNWHIIDAEGKTLGRLASEAAALIRGKHKPQFTPHVDTGDFVVVINA EKIVLTGKKMENKKYYRHSLHPGGLKVTVAQDMVKNKPERMLELAVHGMLPKTRQGDKMK
C12          MRTTYMAKPNEVERNWHIIDAEGKTLGRLASEAAALIRGKHKPQFTPHVDTGDFVVVINA EKIVLTGKKMENKKYYRHSLHPGGLKVTVAQDMVKNKPERMLELAVHGMLPKTRQGDKMK
TH2H2        MRTTYMAKPNEVERNWHIIDAEGKTLGRLASEAAALIRGKHKPQFTPHVDTGDFVVVINA EKIVLTGKKMENKKYYRHSLHPGGLKVTVAQDMVKNKPERMLELAVHGMLPKTRQGDKMK
EB4_G3       MRTTYMAKPNEVERNWHIIDAEGKTLGRLASEAAALIRGKHKPQFTPHVDTGDFVVVINA EKIVLTGKKMENKKYYRHSLHPGGLKVTVAQDMVKNKPERMLELAVHGMLPKTRQGDKMK
CICC_10580   MRTTYMAKPNEVERNWHIIDAEGKTLGRLASEAAALIRGKHKPQFTPHVDTGDFVVVINA EKIVLTGKKMENKKYYRHSLHPGGLKVTVAQDMVKNKPERMLELAVHGMLPKTRQGDKMK
JE201        MRTTYMAKPNEVERNWHIIDAEGKTLGRLASEAAALIRGKHKPQFTPHVDTGDFVVVINA EKIVLTGKKMENKKYYRHSLHPGGLKVTVAQDMVKNKPERMLELAVHGMLPKTRQGDKMK
DCB2         MRTTYMAKPNEVERNWHIIDAEGKTLGRLASEAAALIRGKHKPQFTPHVDTGDFVVVINA EKIVLTGKKMENKKYYRHSLHPGGLKVTVAQDMVKNKPERMLELAVHGMLPKTRQGDKMK
ZF129        MRTTYMAKPNEVERNWHIIDAEGKTLGRLASEAAALIRGKHKPQFTPHVDTGDFVVVINA EKIVLTGKKMENKKYYRHSLHPGGLKVTVAQDMVKNKPERMLELAVHGMLPKTRQGDKMK
KP10         MRTTYMAKPNEVERNWHIIDAEGKTLGRLASEAAALIRGKHKPQFTPHVDTGDFVVVINA EKIVLTGKKMENKKYYRHSLHPGGLKVTVAQDMVKNKPERMLELAVHGMLPKTRQGDKMK
IIF8SW       MRTTYMAKPNEVERNWHIIDAEGKTLGRLASEAAALIRGKHKPQFTPHVDTGDFVVVINA EKIVLTGKKMENKKYYRHSLHPGGLKVTVAQDMVKNKPERMLELAVHGMLPKTRQGDKMK
2A_2A        MRTTYMAKPNEVERNWHIIDAEGKTLGRLASEAAALIRGKHKPQFTPHVDTGDFVVVINA EKIVLTGKKMENKKYYRHSLHPGGLKVTVAQDMVKNKPERMLELAVHGMLPKTRQGDKMK
YS18         MRTTYMAKPNEVERNWHIIDAEGKTLGRLASEAAALIRGKHKPQFTPHVDTGDFVVVINA EKIVLTGKKMENKKYYRHSLHPGGLKVTVAQDMVKNKPERMLELAVHGMLPKTRQGDKMK
181MFCol51   MRTTYMAKPNEVERNWHIIDAEGKTLGRLASEAAALIRGKHKPQFTPHVDTGDFVVVINA EKIVLTGKKMENKKYYRHSLHPGGLKVTVAQDMVKNKPERMLELAVHGMLPKTRQGDKMK
EBL06        MRTTYMAKPNEVERNWHIIDAEGKTLGRLASEAAALIRGKHKPQFTPHVDTGDFVVVINA EKIVLTGKKMENKKYYRHSLHPGGLKVTVAQDMVKNKPERMLELAVHGMLPKTRQGDKMK
HY96         MRTTYMAKPNEVERNWHIIDAEGKTLGRLASEAAALIRGKHKPQFTPHVDTGDFVVVINA EKIVLTGKKMENKKYYRHSLHPGGLKVTVAQDMVKNKPERMLELAVHGMLPKTRQGDKMK
ZJ_9         MRTTYMAKPNEVERNWHIIDAEGKTLGRLASEAAALIRGKHKPQFTPHVDTGDFVVVINA EKIVLTGKKMENKKYYRHSLHPGGLKVTVAQDMVKNKPERMLELAVHGMLPKTRQGDKMK
SQR_21       MRTTYMAKPNEVERNWHIIDAEGKTLGRLASEAAALIRGKHKPQFTPHVDTGDFVVVINA EKIVLTGKKMENKKYYRHSLHPGGLKVTVAQDMVKNKPERMLELAVHGMLPKTRQGDKMK
CF05         MRTTYMAKPNEVERNWHIIDAEGKTLGRLASEAAALIRGKHKPQFTPHVDTGDFVVVINA EKIVLTGKKMENKKYYRHSLHPGGLKVTVAQDMVKNKPERMLELAVHGMLPKTRQGDKMK
KCCM_40454   MRTTYMAKPNEVERNWHIIDAEGKTLGRLASEAAALIRGKHKPQFTPHVDTGDFVVVINA EKIVLTGKKMENKKYYRHSLHPGGLKVTVAQDMVKNKPERMLELAVHGMLPKTRQGDKMK
S3           MRTTYMAKPNEVERNWHIIDAEGKTLGRLASEAAALIRGKHKPQFTPHVDTGDFVVVINA EKIVLTGKKMENKKYYRHSLHPGGLKVTVAQDMVKNKPERMLELAVHGMLPKTRQGDKMK
Sb3          MRTTYMAKPNEVERNWHIIDAEGKTLGRLASEAAALIRGKHKPQFTPHVDTGDFVVVINA EKIVLTGKKMENKKYYRHSLHPGGLKVTVAQDMVKNKPERMLELAVHGMLPKTRQGDKMK
CJX518       MRTTYMAKPNEVERNWHIIDAEGKTLGRLASEAAALIRGKHKPQFTPHVDTGDFVVVINA EKIVLTGKKMENKKYYRHSLHPGGLKVTVAQDMVKNKPERMLELAVHGMLPKTRQGDKMK
KF_1         MRTTYMAKPNEVERNWHIIDAEGKTLGRLASEAAALIRGKHKPQFTPHVDTGDFVVVINA EKIVLTGKKMENKKYYRHSLHPGGLKVTVAQDMVKNKPERMLELAVHGMLPKTRQGDKMK
ND25         MRTTYMAKPNEVERNWHIIDAEGKTLGRLASEAAALIRGKHKPQFTPHVDTGDFVVVINA EKIVLTGKKMENKKYYRHSLHPGGLKVTVAQDMVKNKPERMLELAVHGMLPKTRQGDKMK
1_43         MRTTYMAKPNEVERNWHIIDAEGKTLGRLASEAAALIRGKHKPQFTPHVDTGDFVVVINA EKIVLTGKKMENKKYYRHSLHPGGLKVTVAQDMVKNKPERMLELAVHGMLPKTRQGDKMK
SC2          MRTTYMAKPNEVERNWHIIDAEGKTLGRLASEAAALIRGKHKPQFTPHVDTGDFVVVINA EKIVLTGKKMENKKYYRHSLHPGGLKVTVAQDMVKNKPERMLELAVHGMLPKTRQGDKMK
M1           MRTTYMAKPNEVERNWHIIDAEGKTLGRLASEAAALIRGKHKPQFTPHVDTGDFVVVINA EKIVLTGKKMENKKYYRHSLHPGGLKVTVAQDMVKNKPERMLELAVHGMLPKTRQGDKMK
MES972       MRTTYMAKPNEVERNWHIIDAEGKTLGRLASEAAALIRGKHKPQFTPHVDTGDFVVVINA EKIVLTGKKMENKKYYRHSLHPGGLKVTVAQEMVKNKPERMLELAVHGMLPKTRQGDKMK
YC0573       MRTTYMAKPNEVERNWHIIDAEGKTLGRLASEAAALIRGKHKPQFTPHVDTGDFVVVINA EKIVLTGKKMENKKYYRHSLHPGGLKVTVAQEMVKNKPERMLELAVHGMLPKTRQGEKMK
LY214        MRTTYMAKPNEVERNWHIIDAEGKTLGRLASEAAALIRGKHKPQFTPHVDTGDFVVVINA EKIVLTGKKMENKKYYRHSLHPGGLKVTVAQEMVKNKPERMLELAVHGMLPKTRQGEKMK
CR1          MRTTYMAKPNEVERNWHIIDAEGKTLGRLASEAAALIRGKHKPQFTPHVDTGDFVVVINA EKIVLTGKKMENKKYYRHSLHPGGLKVTVAQEMVKNKPERMLELAVHGMLPKTRQGEKMK
K16          MRTTYMAKPNEVERNWHIIDAEGKTLGRLASEAAALIRGKHKPQFTPHVDTGDFVVVINA EKIVLTGKKMENKKYYRHSLHPGGLKVTVAQEMVKNKPERMLELAVHGMLPKTRQGEKMK
CFSAN034343  MRTTYMAKPNEVERNWHIIDAEGKTLGRLASEAAALIRGKHKPQFTPHVDTGDFVVVINA EKIVLTGKKMENKKYYRHSLHPGGLKVTVAQEMVKNKPERMLELAVHGMLPKTRQGEKMK
CFSAN034342  MRTTYMAKPNEVERNWHIIDAEGKTLGRLASEAAALIRGKHKPQFTPHVDTGDFVVVINA EKIVLTGKKMENKKYYRHSLHPGGLKVTVAQEMVKNKPERMLELAVHGMLPKTRQGEKMK
ATCC_15970   MRTTYMAKPNEVERNWHIIDAEGKTLGRLASEAAALIRGKHKPQFTPHVDTGDFVVVINA EKIVLTGKKMENKKYYRHSLHPGGLKVTVAQEMVKNKPERMLELAVHGMLPKTRQGEKMK
DSM_292      MRTTYMAKPNEVERNWHIIDAEGKTLGRLASEAAALIRGKHKPQFTPHVDTGDFVVVINA EKIVLTGKKMENKKYYRHSLHPGGLKVTVAQEMVKNKPERMLELAVHGMLPKTRQGEKMK
WLY78        MRTTYMAKPNEVERNWHIIDAEGKTLGRLASEAAALIRGKHKPQFTPHVDTGDFVVVINA EKIVLTGKKMENKKYYRHSLHPGGLKVTVAQEMVKNKPERMLELAVHGMLPKTRQGEKMK
MES110       MRTTYMAKPNEVERNWHIIDAEGKTLGRLASEAAALIRGKHKPQFTPHVDTGDFVVVINA EKIVLTGKKMENKKYYRHSLHPGGLKVTVAQEMVKNKPERMLELAVHGMLPKTRQGEKMK
LMG_27872    MRTTYMAKPNEVERNWHIIDAEGKTLGRLASEAAALIRGKHKPQFTPHVDTGDFVVVINA EKIVLTGKKMENKKYYRHSLHPGGLKVTVAQEMVKNKPERMLELAVHGMLPKTRQGEKMK
CFSAN034341  MRTTYMAKPNEVERNWHIIDAEGKTLGRLASEAAALIRGKHKPQFTPHVDTGDFVVVINA EKIVLTGKKMENKKYYRHSLHPGGLKVTVAQEMVKNKPERMLELAVHGMLPKTRQGEKMK
Hob6         MRTTYMAKPNEVERNWHIIDAEGKTLGRLASEAAALIRGKHKPQFTPHVDTGDFVVVINA EKIVLTGKKMENKKYYRHSLHPGGLKVTVAQEMVKNKPERMLELAVHGMLPKTRQGEKMK
MES20        MRTTYMAKPNEVERNWHIIDAEGKTLGRLASEAAALIRGKHKPQFTPHVDTGDFVVVINA EKIVLTGKKMENKKYYRHSLHPGGLKVTVAQEMVKNKPERMLELAVHGMLPKTRQGEKMK
MES17        MRTTYMAKPNEVERNWHIIDAEGKTLGRLASEAAALIRGKHKPQFTPHVDTGDFVVVINA EKIVLTGKKMENKKYYRHSLHPGGLKVTVAQEMVKNKPERMLELAVHGMLPKTRQGEKMK
MES108       MRTTYMAKPNEVERNWHIIDAEGKTLGRLASEAAALIRGKHKPQFTPHVDTGDFVVVINA EKIVLTGKKMENKKYYRHSLHPGGLKVTVAQEMVKNKPERMLELAVHGMLPKTRQGEKMK
CCI_25       MRTTYMAKPNEVERNWHIIDAEGKTLGRLASEAAALIRGKHKPQFTPHVDTGDFVVVINA EKIVLTGKKMENKKYYRHSLHPGGLKVTVAQEMVKNKPERMLELAVHGMLPKTRQGEKMK
J            MRTTYMAKPNEVERNWHIIDAEGKTLGRLASEAAALIRGKHKPQFTPHVDTGDFVVVINA EKIVLTGKKMENKKYYRHSLHPGGLKVTVAQEMVKNKPERMLELAVHGMLPKTRQGEKMK
YUPP_8       MRTTYMAKPNEVERNWHIIDAEGKTLGRLASEAAALIRGKHKPQFTPHVDTGDFVVVINA EKIVLTGKKMENKKYYRHSLHPGGLKVTVAQEMVKNKPERMLELAVHGMLPKTRQGEKMK
E681         MRTTYMAKPNEVERNWHIIDAEGKTLGRLASEAAALIRGKHKPQFTPHVDTGDFVVVINA EKIVLTGKKMENKKYYRHSLHPGGLKVTVAQEMVKNKPERMLELAVHGMLPKTRQGEKMK
YC0136       MRTTYMAKPNEVERNWHIIDAEGKTLGRLASEAAALIRGKHKPQFTPHVDTGDFVVVINA EKIVLTGKKMENKKYYRHSLHPGGLKVTVAQEMVKNKPERMLELAVHGMLPKTRQGEKMK
P3           MRTTYMAKPNEVERNWHIIDAEGKTLGRLASEAAALIRGKHKPQFTPHVDTGDFVVVINA EKIVLTGKKMENKKYYRHSLHPGGLKVTVAQEMVKNKPERMLELAVHGMLPKTRQGEKMK
DSM_365      MRTTYMAKPNEVERNWHIIDAEGKTLGRLASEAAALIRGKHKPQFTPHVDTGDFVVVINA EKIVLTGKKMENKKYYRHSLHPGGLKVTVAQEMVKNKPERMLELAVHGMLPKTRQGEKMK
ZF197        MRTTYMAKPNEVERNWHIIDAEGKTLGRLASEAAALIRGKHKPQFTPHVDTGDFVIVINA EKIVLTGKKMENKKYYRHSMHPGGLKVTVAQDMVKNKPERMLELAVHGMLPKTRQGDKMK
ND24         MRTTYMAKPNEVERNWHIIDAEGKTLGRLASEAAALIRGKHKPQFTPHVDTGDFVIVINA EKIVLTGKKMENKKYYRHSMHPGGLKVTVAQDMVKNKPERMLELAVHGMLPKTRQGEKMK
P.ottowii    MRTTYMAKPNEVERNWHIIDAEGKTLGRLASEAAALIRGKHKPQFTPHVDTGDFVIVINA EKIVLTGKKMENKKYYRHSMHPGGLKVTVAQDMVKNKPERMLELAVHGMLPKTRQGEKMK
             *******************************************************:**** *******************:***********:************************:***
```

```
Cluster 1      MRTTYMAKPNEVERNWHIIDAEGKTLGRLASEAAALIRGKHKPQFTPHVDTGDFVVVINAEKIVLTGKKMENKKYYRHSLHPGGLKVTVAQEMVKNKPERMLELAVHGMLPKTRQGEKMK
Cluster 2      MRTTYMAKPNEVERNWHIIDAEGKTLGRLASEAAALIRGKHKPQFTPHVDTGDFVIVINAEKIVLTGKKMENKKYYRHSMHPGGLKVTVAQDMVKNKPERMLELAVHGMLPKTRQGEKMK
Cluster 3,4    MRTTYMAKPNEVERNWHIIDAEGKTLGRLASEAAALIRGKHKPQFTPHVDTGDFVVVINAEKIVLTGKKMENKKYYRHSLHPGGLKVTVAQDMVKNKPERMLELAVHGMLPKTRQGDKMK
```

```
OSY_DF          LRLKAYRGTEHPHAAQKPEVYELRG
OSY_EC          LRLKAYRGTEHPHAAQKPEVYELRG
ATCC_842        LRLKAYRGTEHPHAAQKPEVYELRG
NRRL_B_30509    LRLKAYRGTEHPHAAQKPEVYELRG
NCTC7575        LRLKAYRGTEHPHAAQKPEVYELRG
MEZ6            LRLKAYRGTEHPHAAQKPEVYELRG
O_211           LRLKAYRGTEHPHAAQKPEVYELRG
HK4             LRLKAYRGTEHPHAAQKPEVYELRG
A18             LRLKAYRGTEHPHAAQKPEVYELRG
C12             LRLKAYRGTEHPHAAQKPEVYELRG
TH2H2           LRLKAYRGTEHPHAAQKPEVYELRG
EB4_G3          LRLKAYRGTEHPHAAQKPEVYELRG
CICC_10580      LRLKAYRGTEHPHAAQKPEVYELRG
JE201           LRLKAYRGTEHPHAAQKPEVYELRG
DCB2            LRLKAYRGTEHPHAAQKPEVYELRG
ZF129           LRLKAYRGTEHPHAAQKPEVYELRG
KP10            LRLKAYRGTEHPHAAQKPEVYELRG
IIF8SW          LRLKAYRGTEHPHAAQKPEVYELRG
2A_2A           LRLKAYRGTEHPHAAQKPEVYELRG
YS18            LRLKAYRGTEHPHAAQKPEVYELRG
181MFCol51      LRLKAYRGTEHPHAAQKPEVYELRG
EBL06           LRLKAYRGTEHPHAAQKPEVYELRG
HY96            LRLKAYRGTEHPHAAQKPEVYELRG
ZJ_9            LRLKAYRGTEHPHAAQKPEVYELRG
SQR_21          LRLKAYRGTEHPHAAQKPEVYELRG
CF05            LRLKAYRGTEHPHAAQKPEVYELRG
KCCM_40454      LRLKAYRGTEHPHAAQKPEVYELRG
S3              LRLKAYRGTEHPHAAQKPEVYELRG
Sb3             LRLKAYRGTEHPHAAQKPEVYELRG
CJX518          LRLKAYRGTEHPHAAQKPEVYELRG
KF_1            LRLKAYRGTEHPHAAQKPEVYELRG
ND25            LRLKAYRGTEHPHAAQKPEVYELRG
1_43            LRLKAYRGTEHPHAAQKPEVYELRG
SC2             LRLKAYRGTEHPHAAQKPEVYELRG
M1              LRLKAYRGTEHPHAAQKPEVYELRG
MES972          LRLKAYRGTEHPHAAQKPEVYELRG
YC0573          LRLKAYRGTEHPHAAQKPEVYELRG
LY214           LRLKAYRGTEHPHAAQKPEVYELRG
CR1             LRLKAYRGTEHPHAAQKPEVYELRG
K16             LRLKAYRGTEHPHAAQKPEVYELRG
CFSAN034343     LRLKAYRGTEHPHAAQKPEVYELRG
CFSAN034342     LRLKAYRGTEHPHAAQKPEVYELRG
ATCC_15970      LRLKAYRGTEHPHAAQKPEVYELRG
DSM_292         LRLKAYRGTEHPHAAQKPEVYELRG
WLY78           LRLKAYRGTEHPHAAQKPEVYELRG
MES110          LRLKAYRGTEHPHAAQKPEVYELRG
LMG_27872       LRLKAYRGTEHPHAAQKPEVYELRG
CFSAN034341     LRLKAYRGTEHPHAAQKPEVYELRG
Hob6            LRLKAYRGTEHPHAAQKPEVYELRG
MES20           LRLKAYRGTEHPHAAQKPEVYELRG
MES17           LRLKAYRGTEHPHAAQKPEVYELRG
MES108          LRLKAYRGTEHPHAAQKPEVYELRG
CCI_25          LRLKAYRGTEHPHAAQKPEVYELRG
J               LRLKAYRGTEHPHAAQKPEVYELRG
YUPP_8          LRLKAYRGTEHPHAAQKPEVYELRG
E681            LRLKAYRGTEHPHAAQKPEVYELRG
YC0136          LRLKAYRGTEHPHAAQKPEVYELRG
P3              LRLKAYRGTEHPHAAQKPEVYELRG
DSM_365         LRLKAYRGTEHPHVAQKPEVYELRG
ZF197           LRLKAYRGTEHPHAAQKPEVYELRG
ND24            LRLKAYRGTEHPHAAQKPEVYELRG
P.ottowii       LRLKAYRGTEHPHAAQKPEVYELRG
                *************.**********

Cluster 1,2,3,4   LRLKAYRGTEHPHAAQKPEVYELRG
```

Figure S9. Multiple alignment of protein L13. The sequences were obtained from the strains belonging to Cluster 1 (yellow), Cluster 2 (gray), Cluster 3 (green), and Cluster 4 (cyan). The consensus sequences of each Cluster are displayed in the box below the alignment. Cluster-specific amino acid substitutions are highlighted using the same colors of the Clusters. Strains DSM 365, MES972 and ZF197, have additional amino acid changes, which produce variability intra-clusters in position 117 (for DSM 365 and MES972) or 134 (for ZF197). The changed amino acids are in bold and underlined in the alignment.

rplN

```
ZF197        ATGATTCAACCATTTACACGTTTGACTGTAGCCGACAATTCCGGCGCGAAGGAACTGATG TGTATCCGTGTGCTCGGTGGAACGGGACGTCGTACAGCTCAAATTGGTGACCTGATCGTT
ND24         ATGATTCAACCATTTACACGTTTGACTGTAGCCGACAATTCCGGCGCGAAGGAACTGATG TGTATCCGTGTGCTCGGTGGAACGGGACGTCGTACAGCTCAAATTGGTGACCTGATCGTT
P.ottowii    ATGATTCAACCATTTACACGTTTGACTGTAGCCGACAATTCCGGCGCGAAGGAACTGATG TGTATCCGTGTGCTCGGTGGAACGGGACGTCGTACAGCTCAAATTGGTGACCTGATCGTT
E681         ATGATTCAACCATTTACACGTTTGACTGTAGCTGACAATTCCGGCGCGAAGGAACTGATG TGTATCCGTGTGCTCGGTGGTACGGGACGTCGTACAGCTCAAATTGGTGACCTGATCGTT
MES20        ATGATTCAACCATTTACACGTTTGACTGTAGCTGACAATTCCGGCGCGAAGGAACTGATG TGTATCCGTGTGCTCGGTGGTACGGGACGTCGTACAGCTCAAATTGGTGACCTGATCGTT
MES17        ATGATTCAACCATTTACACGTTTGACTGTAGCTGACAATTCCGGCGCGAAGGAACTGATG TGTATCCGTGTGCTCGGTGGTACGGGACGTCGTACAGCTCAAATTGGTGACCTGATCGTT
MES108       ATGATTCAACCATTTACACGTTTGACTGTAGCTGACAATTCCGGCGCGAAGGAACTGATG TGTATCCGTGTGCTCGGTGGTACGGGACGTCGTACAGCTCAAATTGGTGACCTGATCGTT
CCI_25       ATGATTCAACCATTTACACGTTTGACTGTAGCTGACAATTCCGGCGCGAAGGAACTGATG TGTATCCGTGTGCTCGGTGGTACGGGACGTCGTACAGCTCAAATTGGTGACCTGATCGTT
J            ATGATTCAACCATTTACACGTTTGACTGTAGCTGACAATTCCGGCGCGAAGGAACTGATG TGTATCCGTGTGCTCGGTGGTACGGGACGTCGTACAGCTCAAATTGGTGACCTGATCGTT
YUPP_8       ATGATTCAACCATTTACACGTTTGACTGTAGCTGACAATTCCGGCGCGAAGGAACTGATG TGTATCCGTGTGCTCGGTGGTACGGGACGTCGTACAGCTCAAATTGGTGACCTGATCGTT
YC0573       ATGATTCAACCATTTACACGTTTGACTGTAGCTGACAATTCCGGCGCGAAGGAACTGATG TGTATCCGTGTGCTCGGTGGTACGGGACGTCGTACAGCTCAAATTGGTGACCTGATCGTT
LY214        ATGATTCAACCATTTACACGTTTGACTGTAGCTGACAATTCCGGCGCGAAGGAACTGATG TGTATCCGTGTGCTCGGTGGTACGGGACGTCGTACAGCTCAAATTGGTGACCTGATCGTT
CR1          ATGATTCAACCATTTACACGTTTGACTGTAGCTGACAATTCCGGCGCGAAGGAACTGATG TGTATCCGTGTGCTCGGTGGTACGGGACGTCGTACAGCTCAAATTGGTGACCTGATCGTT
K16          ATGATTCAACCATTTACACGTTTGACTGTAGCTGACAATTCCGGCGCGAAGGAACTGATG TGTATCCGTGTGCTCGGTGGTACGGGACGTCGTACAGCTCAAATTGGTGACCTGATCGTT
CFSAN034343  ATGATTCAACCATTTACACGTTTGACTGTAGCTGACAATTCCGGCGCGAAGGAACTGATG TGTATCCGTGTGCTCGGTGGTACGGGACGTCGTACAGCTCAAATTGGTGACCTGATCGTT
CFSAN034342  ATGATTCAACCATTTACACGTTTGACTGTAGCTGACAATTCCGGCGCGAAGGAACTGATG TGTATCCGTGTGCTCGGTGGTACGGGACGTCGTACAGCTCAAATTGGTGACCTGATCGTT
ATCC_15970   ATGATTCAACCATTTACACGTTTGACTGTAGCTGACAATTCCGGCGCGAAGGAACTGATG TGTATCCGTGTGCTCGGTGGTACGGGACGTCGTACAGCTCAAATTGGTGACCTGATCGTT
DSM_292      ATGATTCAACCATTTACACGTTTGACTGTAGCTGACAATTCCGGCGCGAAGGAACTGATG TGTATCCGTGTGCTCGGTGGTACGGGACGTCGTACAGCTCAAATTGGTGACCTGATCGTT
WLY78        ATGATTCAACCATTTACACGTTTGACTGTAGCTGACAATTCCGGCGCGAAGGAACTGATG TGTATCCGTGTGCTCGGTGGTACGGGACGTCGTACAGCTCAAATTGGTGACCTGATCGTT
DSM_365      ATGATTCAACCATTTACACGTTTGACTGTAGCTGACAATTCCGGCGCGAAGGAACTGATG TGTATCCGTGTGCTCGGTGGTACGGGACGTCGTACAGCTCAAATTGGTGACCTGATCGTT
MES110       ATGATTCAACCATTTACACGTTTGACTGTAGCTGACAATTCCGGCGCGAAGGAACTGATG TGTATCCGTGTGCTCGGTGGTACGGGACGTCGTACAGCTCAAATTGGTGACCTGATCGTT
LMG_27872    ATGATTCAACCATTTACACGTTTGACTGTAGCTGACAATTCCGGCGCGAAGGAACTGATG TGTATCCGTGTGCTCGGTGGTACGGGACGTCGTACAGCTCAAATTGGTGACCTGATCGTT
CFSAN034341  ATGATTCAACCATTTACACGTTTGACTGTAGCTGACAATTCCGGCGCGAAGGAACTGATG TGTATCCGTGTGCTCGGTGGTACGGGACGTCGTACAGCTCAAATTGGTGACCTGATCGTT
Hob6         ATGATTCAACCATTTACACGTTTGACTGTAGCTGACAATTCCGGCGCGAAGGAACTGATG TGTATCCGTGTGCTCGGTGGTACGGGACGTCGTACAGCTCAAATTGGTGACCTGATCGTT
MES972       ATGATTCAACCATTTACACGTTTGACTGTAGCTGACAATTCCGGCGCGAAGGAACTGATG TGTATCCGTGTGCTCGGTGGTACGGGACGTCGTACAGCTCAAATTGGTGACCTGATCGTT
YC0136       ATGATTCAACCATTTACACGTTTGACTGTAGCTGACAATTCCGGCGCGAAGGAACTGATG TGTATCCGTGTGCTCGGTGGTACGGGACGTCGTACAGCTCAAATTGGTGACCTGATCGTT
P3           ATGATTCAACCATTTACACGTTTGACTGTAGCTGACAATTCCGGCGCGAAGGAACTGATG TGTATCCGTGTGCTCGGTGGTACGGGACGTCGTACAGCTCAAATTGGTGACCTGATCGTT
Sb3          ATGATTCAACCATTTACACGTTTGACTGTGGCTGACAATTCCGGCGCGAAGGAACTGATG TGTATCCGTGTGCTCGGTGGTACGGGACGTCGTACAGCTCAAATTGGTGACCTGATCGTT
CJX518       ATGATTCAACCATTTACACGTTTGACTGTGGCTGACAATTCCGGCGCGAAGGAACTGATG TGTATCCGTGTGCTCGGTGGTACGGGACGTCGTACAGCTCAAATTGGTGACCTGATCGTT
KF_1         ATGATTCAACCATTTACACGTTTGACTGTGGCTGACAATTCCGGCGCGAAGGAACTGATG TGTATCCGTGTGCTCGGTGGTACGGGACGTCGTACAGCTCAAATTGGTGACCTGATCGTT
ND25         ATGATTCAACCATTTACACGTTTGACTGTGGCTGACAATTCCGGCGCGAAGGAACTGATG TGTATCCGTGTGCTCGGTGGTACGGGACGTCGTACAGCTCAAATTGGTGACCTGATCGTT
SC2          ATGATTCAACCATTTACACGTTTGACTGTGGCTGACAATTCCGGCGCGAAGGAACTGATG TGTATCCGTGTGCTCGGTGGTACGGGACGTCGTACAGCTCAAATTGGTGACCTGATCGTT
M1           ATGATTCAACCATTTACACGTTTGACTGTGGCTGACAATTCCGGCGCGAAGGAACTGATG TGTATCCGTGTGCTCGGTGGTACGGGACGTCGTACAGCTCAAATTGGTGACCTGATCGTT
S3           ATGATTCAACCATTTACACGTTTGACTGTAGCTGACAATTCCGGCGCGAAGGAACTGATG TGTATCCGTGTGCTCGGTGGTACGGGACGTCGTACAGCTCAAATTGGTGACCTGATCGTT
HY96         ATGATTCAACCATTTACACGTTTGACTGTAGCTGACAATTCCGGCGCGAAGGAACTGATG TGTATCCGTGTGCTCGGTGGTACGGGACGTCGTACAGCTCAAATTGGTGACCTGATCGTT
OSY_DF       ATGATTCAACCATTTACACGTTTGACTGTAGCTGACAATTCCGGCGCGAAGGAACTGATG TGTATCCGTGTGCTCGGTGGTACGGGACGTCGTACAGCTCAAATTGGTGACCTGATCGTT
OSY_EC       ATGATTCAACCATTTACACGTTTGACTGTAGCTGACAATTCCGGCGCGAAGGAACTGATG TGTATCCGTGTGCTCGGTGGTACGGGACGTCGTACAGCTCAAATTGGTGACCTGATCGTT
ATCC_842     ATGATTCAACCATTTACACGTTTGACTGTAGCTGACAATTCCGGCGCGAAGGAACTGATG TGTATCCGTGTGCTCGGTGGTACGGGACGTCGTACAGCTCAAATTGGTGACCTGATCGTT
NRRL_B_30509 ATGATTCAACCATTTACACGTTTGACTGTAGCTGACAATTCCGGCGCGAAGGAACTGATG TGTATCCGTGTGCTCGGTGGTACGGGACGTCGTACAGCTCAAATTGGTGACCTGATCGTT
NCTC7575     ATGATTCAACCATTTACACGTTTGACTGTAGCTGACAATTCCGGCGCGAAGGAACTGATG TGTATCCGTGTGCTCGGTGGTACGGGACGTCGTACAGCTCAAATTGGTGACCTGATCGTT
MEZ6         ATGATTCAACCATTTACACGTTTGACTGTAGCTGACAATTCCGGCGCGAAGGAACTGATG TGTATCCGTGTGCTCGGTGGTACGGGACGTCGTACAGCTCAAATTGGTGACCTGATCGTT
O_211        ATGATTCAACCATTTACACGTTTGACTGTAGCTGACAATTCCGGCGCGAAGGAACTGATG TGTATCCGTGTGCTCGGTGGTACGGGACGTCGTACAGCTCAAATTGGTGACCTGATCGTT
HK4          ATGATTCAACCATTTACACGTTTGACTGTAGCTGACAATTCCGGCGCGAAGGAACTGATG TGTATCCGTGTGCTCGGTGGTACGGGACGTCGTACAGCTCAAATTGGTGACCTGATCGTT
A18          ATGATTCAACCATTTACACGTTTGACTGTAGCTGACAATTCCGGCGCGAAGGAACTGATG TGTATCCGTGTGCTCGGTGGTACGGGACGTCGTACAGCTCAAATTGGTGACCTGATCGTT
C12          ATGATTCAACCATTTACACGTTTGACTGTAGCTGACAATTCCGGCGCGAAGGAACTGATG TGTATCCGTGTGCTCGGTGGTACGGGACGTCGTACAGCTCAAATTGGTGACCTGATCGTT
TH2H2        ATGATTCAACCATTTACACGTTTGACTGTAGCTGACAATTCCGGCGCGAAGGAACTGATG TGTATCCGTGTGCTCGGTGGTACGGGACGTCGTACAGCTCAAATTGGTGACCTGATCGTT
EB4_G3       ATGATTCAACCATTTACACGTTTGACTGTAGCTGACAATTCCGGCGCGAAGGAACTGATG TGTATCCGTGTGCTCGGTGGTACGGGACGTCGTACAGCTCAAATTGGTGACCTGATCGTT
CICC_10580   ATGATTCAACCATTTACACGTTTGACTGTAGCTGACAATTCCGGCGCGAAGGAACTGATG TGTATCCGTGTGCTCGGTGGTACGGGACGTCGTACAGCTCAAATTGGTGACCTGATCGTT
JE201        ATGATTCAACCATTTACACGTTTGACTGTAGCTGACAATTCCGGCGCGAAGGAACTGATG TGTATCCGTGTGCTCGGTGGTACGGGACGTCGTACAGCTCAAATTGGTGACCTGATCGTT
DCB2         ATGATTCAACCATTTACACGTTTGACTGTAGCTGACAATTCCGGCGCGAAGGAACTGATG TGTATCCGTGTGCTCGGTGGTACGGGACGTCGTACAGCTCAAATTGGTGACCTGATCGTT
ZF129        ATGATTCAACCATTTACACGTTTGACTGTAGCTGACAATTCCGGCGCGAAGGAACTGATG TGTATCCGTGTGCTCGGTGGTACGGGACGTCGTACAGCTCAAATTGGTGACCTGATCGTT
KP10         ATGATTCAACCATTTACACGTTTGACTGTAGCTGACAATTCCGGCGCGAAGGAACTGATG TGTATCCGTGTGCTCGGTGGTACGGGACGTCGTACAGCTCAAATTGGTGACCTGATCGTT
IIF8SW       ATGATTCAACCATTTACACGTTTGACTGTAGCTGACAATTCCGGCGCGAAGGAACTGATG TGTATCCGTGTGCTCGGTGGTACGGGACGTCGTACAGCTCAAATTGGTGACCTGATCGTT
2A_2A        ATGATTCAACCATTTACACGTTTGACTGTAGCTGACAATTCCGGCGCGAAGGAACTGATG TGTATCCGTGTGCTCGGTGGTACGGGACGTCGTACAGCTCAAATTGGTGACCTGATCGTT
181MFCol51   ATGATTCAACCATTTACACGTTTGACTGTAGCTGACAATTCCGGCGCGAAGGAACTGATG TGTATCCGTGTGCTCGGTGGTACGGGACGTCGTACAGCTCAAATTGGTGACCTGATCGTT
EBL06        ATGATTCAACCATTTACACGTTTGACTGTAGCTGACAATTCCGGCGCGAAGGAACTGATG TGTATCCGTGTGCTCGGTGGTACGGGACGTCGTACAGCTCAAATTGGTGACCTGATCGTT
ZJ_9         ATGATTCAACCATTTACACGTTTGACTGTAGCTGACAATTCCGGCGCGAAGGAACTGATG TGTATCCGTGTGCTCGGTGGTACGGGACGTCGTACAGCTCAAATTGGTGACCTGATCGTT
SQR_21       ATGATTCAACCATTTACACGTTTGACTGTAGCTGACAATTCCGGCGCGAAGGAACTGATG TGTATCCGTGTGCTCGGTGGTACGGGACGTCGTACAGCTCAAATTGGTGACCTGATCGTT
CF05         ATGATTCAACCATTTACACGTTTGACTGTAGCTGACAATTCCGGCGCGAAGGAACTGATG TGTATCCGTGTGCTCGGTGGTACGGGACGTCGTACAGCTCAAATTGGTGACCTGATCGTT
KCCM_40454   ATGATTCAACCATTTACACGTTTGACTGTAGCTGACAATTCCGGCGCGAAGGAACTGATG TGTATCCGTGTGCTCGGTGGTACGGGACGTCGTACAGCTCAAATTGGTGACCTGATCGTT
1_43         ATGATTCAACCATTTACACGTTTGACTGTAGCTGACAATTCCGGCGCGAAGGAACTGATG TGTATCCGTGTGCTCGGTGGTACGGGACGTCGTACAGCTCAAATTGGTGACCTGATCGTT
YS18         ATGATTCAACCATTTACACGTTTGACTGTAGCTGACAATTCCGGCGCGAAGGAACTGATG TGTATCCGTGTGCTCGGTGGTACGGGACGTCGTACAGCTCAAATTGGTGACCTGATCGTT
             ***************************** ** *************************** ******************** ***************************************
```

```
Cluster 1,3,4   AGCTGACAATTCCGGCGCGAAGGAACTGATGTGTATCCGTGTGCTCGGTGGTACGGGACGTCGTACAGCTCAAATTGGTGACCTGATCGTT
Cluster 2       AGCCGACAATTCCGGCGCGAAGGAACTGATGTGTATCCGTGTGCTCGGTGGAACGGGACGTCGTACAGCTCAAATTGGTGACCTGATCGTT
```

```
ZF197         TGTTCCGTAAAACAAGCAACACCAGGCGGCGTTGTCAAAAAGGGTGATGTAGTAAGAGCG GTTGTTGTTCGTACTAAACGTTCTATCCGCCGTAAAGACGGTTCCTACATCGGATTTGAT
ND24          TGTTCCGTAAAACAAGCAACACCAGGCGGCGTTGTCAAAAAGGGTGATGTAGTAAGAGCG GTTGTTGTTCGTACTAAACGTTCTATCCGCCGTAAAGACGGTTCCTACATCGGATTTGAT
P.ottowii     TGTTCCGTAAAACAAGCAACACCAGGCGGCGTTGTCAAAAAGGGTGATGTAGTAAGAGCG GTTGTTGTTCGTACTAAACGTTCTATCCGCCGTAAAGACGGTTCCTACATCGGATTTGAT
E681          TGTTCCGTAAAACAAGCAACACCAGGCGGCGTTGTCAAAAAGGGTGATGTAGTAAGAGCG GTTGTTGTTCGTACTAAACGTTCTATCCGCCGTAAAGACGGTTCTTACATCGGATTTGAC
MES20         TGTTCCGTAAAACAAGCAACACCAGGCGGCGTTGTCAAAAAGGGTGATGTAGTAAGAGCG GTTGTTGTTCGTACTAAACGTTCTATCCGCCGTAAAGACGGTTCCTACATCGGATTTGAC
MES17         TGTTCCGTAAAACAAGCAACACCAGGCGGCGTTGTCAAAAAGGGTGATGTAGTAAGAGCG GTTGTTGTTCGTACTAAACGTTCTATCCGCCGTAAAGACGGTTCCTACATCGGATTTGAC
MES108        TGTTCCGTAAAACAAGCAACACCAGGCGGCGTTGTCAAAAAGGGTGATGTAGTAAGAGCG GTTGTTGTTCGTACTAAACGTTCTATCCGCCGTAAAGACGGTTCCTACATCGGATTTGAC
CCI_25        TGTTCCGTAAAACAAGCAACACCAGGCGGCGTTGTCAAAAAGGGTGATGTAGTAAGAGCG GTTGTTGTTCGTACTAAACGTTCTATCCGCCGTAAAGACGGTTCCTACATCGGATTTGAC
J             TGTTCCGTAAAACAAGCAACACCAGGCGGCGTTGTCAAAAAGGGTGATGTAGTAAGAGCG GTTGTTGTTCGTACTAAACGTTCTATCCGCCGTAAAGACGGTTCCTACATCGGATTTGAC
YUPP_8        TGTTCCGTAAAACAAGCAACACCAGGCGGCGTTGTCAAAAAGGGTGATGTAGTAAGAGCG GTTGTTGTTCGTACTAAACGTTCTATCCGCCGTAAAGACGGTTCCTACATCGGATTTGAC
YC0573        TGTTCCGTAAAACAAGCAACACCAGGCGGCGTTGTCAAAAAGGGTGATGTAGTAAGAGCG GTTGTTGTTCGTACTAAACGTTCTATCCGCCGTAAAGACGGTTCCTACATCGGATTTGAC
LY214         TGTTCCGTAAAACAAGCAACACCAGGCGGCGTTGTCAAAAAGGGTGATGTAGTAAGAGCG GTTGTTGTTCGTACTAAACGTTCTATCCGCCGTAAAGACGGTTCCTACATCGGATTTGAC
CR1           TGTTCCGTAAAACAAGCAACACCAGGCGGCGTTGTCAAAAAGGGTGATGTAGTAAGAGCG GTTGTTGTTCGTACTAAACGTTCTATCCGCCGTAAAGACGGTTCCTACATCGGATTTGAC
K16           TGTTCCGTAAAACAAGCAACACCAGGCGGCGTTGTCAAAAAGGGTGATGTAGTAAGAGCG GTTGTTGTTCGTACTAAACGTTCTATCCGCCGTAAAGACGGTTCCTACATCGGATTTGAC
CFSAN034343   TGTTCCGTAAAACAAGCAACACCAGGCGGCGTTGTCAAAAAGGGTGATGTAGTAAGAGCG GTTGTTGTTCGTACTAAACGTTCTATCCGCCGTAAAGACGGTTCCTACATCGGATTTGAC
CFSAN034342   TGTTCCGTAAAACAAGCAACACCAGGCGGCGTTGTCAAAAAGGGTGATGTAGTAAGAGCG GTTGTTGTTCGTACTAAACGTTCTATCCGCCGTAAAGACGGTTCCTACATCGGATTTGAC
ATCC_15970    TGTTCCGTAAAACAAGCAACACCAGGCGGCGTTGTCAAAAAGGGTGATGTAGTAAGAGCG GTTGTTGTTCGTACTAAACGTTCTATCCGCCGTAAAGACGGTTCCTACATCGGATTTGAC
DSM_292       TGTTCCGTAAAACAAGCAACACCAGGCGGCGTTGTCAAAAAGGGTGATGTAGTAAGAGCG GTTGTTGTTCGTACTAAACGTTCTATCCGCCGTAAAGACGGTTCCTACATCGGATTTGAC
WLY78         TGTTCCGTAAAACAAGCAACACCAGGCGGCGTTGTCAAAAAGGGTGATGTAGTAAGAGCG GTTGTTGTTCGTACTAAACGTTCTATCCGCCGTAAAGACGGTTCCTACATCGGATTTGAC
DSM_365       TGTTCCGTAAAACAAGCAACACCAGGCGGCGTTGTCAAAAAGGGTGATGTAGTAAGAGCG GTTGTTGTTCGTACTAAACGTTCTATCCGCCGTAAAGACGGTTCCTACATCGGATTTGAC
MES110        TGTTCCGTAAAACAAGCAACACCAGGCGGCGTTGTCAAAAAGGGTGATGTAGTAAGAGCG GTTGTTGTTCGTACTAAACGTTCTATCCGCCGTAAAGACGGTTCCTACATCGGATTTGAC
LMG_27872     TGTTCCGTAAAACAAGCAACACCAGGCGGCGTTGTCAAAAAGGGTGATGTAGTAAGAGCG GTTGTTGTTCGTACTAAACGTTCTATCCGCCGTAAAGACGGTTCCTACATCGGATTTGAC
CFSAN034341   TGTTCCGTAAAACAAGCAACACCAGGCGGCGTTGTCAAAAAGGGTGATGTAGTAAGAGCG GTTGTTGTTCGTACTAAACGTTCTATCCGCCGTAAAGACGGTTCCTACATCGGATTTGAC
Hob6          TGTTCCGTAAAACAAGCAACACCAGGCGGCGTTGTCAAAAAGGGTGATGTAGTAAGAGCG GTTGTTGTTCGTACTAAACGTTCTATCCGCCGTAAAGACGGTTCCTACATCGGATTTGAC
MES972        TGTTCCGTAAAACAAGCAACACCAGGCGGCGTTGTCAAAAAGGGTGATGTAGTAAGAGCG GTTGTTGTTCGTACTAAACGTTCTATCCGCCGTAAAGACGGTTCCTACATCGGATTTGAC
YC0136        TGTTCCGTAAAACAAGCAACACCAGGCGGCGTTGTCAAAAAGGGTGATGTAGTAAGAGCG GTTGTTGTTCGTACTAAACGTTCTATCCGCCGTAAAGACGGTTCCTACATCGGATTTGAC
P3            TGTTCCGTAAAACAAGCAACACCAGGCGGCGTTGTCAAAAAGGGTGATGTAGTAAGAGCG GTTGTTGTTCGTACTAAACGTTCTATCCGCCGTAAAGACGGTTCCTACATCGGATTTGAC
Sb3           TGTTCCGTAAAACAAGCAACACCAGGCGGCGTTGTCAAAAAGGGTGATGTAGTAAGAGCG GTTGTCGTTCGTACTAAACGTTCTATCCGCCGTAAAGACGGTTCCTACATCGGATTTGAT
CJX518        TGTTCCGTAAAACAAGCAACACCAGGCGGCGTTGTCAAAAAGGGTGATGTAGTAAGAGCG GTTGTCGTTCGTACTAAACGTTCTATCCGCCGTAAAGACGGTTCCTACATCGGATTTGAT
KF_1          TGTTCCGTAAAACAAGCAACACCAGGCGGCGTTGTCAAAAAGGGTGATGTAGTAAGAGCG GTTGTCGTTCGTACTAAACGTTCTATCCGCCGTAAAGACGGTTCCTACATCGGATTTGAT
ND25          TGTTCCGTAAAACAAGCAACACCAGGCGGCGTTGTCAAAAAGGGTGATGTAGTAAGAGCG GTTGTCGTTCGTACTAAACGTTCTATCCGCCGTAAAGACGGTTCCTACATCGGATTTGAT
SC2           TGTTCCGTAAAACAAGCAACACCAGGCGGCGTTGTCAAAAAGGGTGATGTAGTAAGAGCG GTTGTCGTTCGTACTAAACGTTCTATCCGCCGTAAAGACGGTTCCTACATCGGATTTGAT
M1            TGTTCCGTAAAACAAGCAACACCAGGCGGCGTTGTCAAAAAGGGTGATGTAGTAAGAGCG GTTGTCGTTCGTACTAAACGTTCTATCCGCCGTAAAGACGGTTCCTACATCGGATTTGAT
S3            TGTTCCGTAAAACAAGCAACACCAGGCGGCGTTGTCAAAAAGGGTGATGTAGTAAGAGCG GTTGTCGTTCGCACTAAACGTTCTATCCGCCGTAAAGACGGTTCCTACATCGGATTTGAT
HY96          TGTTCCGTAAAACAAGCAACACCAGGCGGCGTTGTCAAAAAGGGTGATGTAGTAAGAGCG GTTGTCGTTCGCACTAAACGTTCTATCCGCCGTAAAGACGGTTCCTACATCGGATTTGAT
OSY_DF        TGTTCCGTAAAACAAGCAACACCAGGCGGCGTTGTCAAAAAGGGTGATGTAGTAAGAGCG GTTGTCGTTCGCACTAAACGTTCTATCCGCCGTAAAGACGGTTCCTACATCGGATTTGAT
OSY_EC        TGTTCCGTAAAACAAGCAACACCAGGCGGCGTTGTCAAAAAGGGTGATGTAGTAAGAGCG GTTGTCGTTCGCACTAAACGTTCTATCCGCCGTAAAGACGGTTCCTACATCGGATTTGAT
ATCC_842      TGTTCCGTAAAACAAGCAACACCAGGCGGCGTTGTCAAAAAGGGTGATGTAGTAAGAGCG GTTGTCGTTCGCACTAAACGTTCTATCCGCCGTAAAGACGGTTCCTACATCGGATTTGAT
NRRL_B_30509  TGTTCCGTAAAACAAGCAACACCAGGCGGCGTTGTCAAAAAGGGTGATGTAGTAAGAGCG GTTGTCGTTCGCACTAAACGTTCTATCCGCCGTAAAGACGGTTCCTACATCGGATTTGAT
NCTC7575      TGTTCCGTAAAACAAGCAACACCAGGCGGCGTTGTCAAAAAGGGTGATGTAGTAAGAGCG GTTGTCGTTCGCACTAAACGTTCTATCCGCCGTAAAGACGGTTCCTACATCGGATTTGAT
MEZ6          TGTTCCGTAAAACAAGCAACACCAGGCGGCGTTGTCAAAAAGGGTGATGTAGTAAGAGCG GTTGTCGTTCGCACTAAACGTTCTATCCGCCGTAAAGACGGTTCCTACATCGGATTTGAT
O_211         TGTTCCGTAAAACAAGCAACACCAGGCGGCGTTGTCAAAAAGGGTGATGTAGTAAGAGCG GTTGTCGTTCGCACTAAACGTTCTATCCGCCGTAAAGACGGTTCCTACATCGGATTTGAT
HK4           TGTTCCGTAAAACAAGCAACACCAGGCGGCGTTGTCAAAAAGGGTGATGTAGTAAGAGCG GTTGTCGTTCGCACTAAACGTTCTATCCGCCGTAAAGACGGTTCCTACATCGGATTTGAT
A18           TGTTCCGTAAAACAAGCAACACCAGGCGGCGTTGTCAAAAAGGGTGATGTAGTAAGAGCG GTTGTCGTTCGCACTAAACGTTCTATCCGCCGTAAAGACGGTTCCTACATCGGATTTGAT
C12           TGTTCCGTAAAACAAGCAACACCAGGCGGCGTTGTCAAAAAGGGTGATGTAGTAAGAGCG GTTGTCGTTCGCACTAAACGTTCTATCCGCCGTAAAGACGGTTCCTACATCGGATTTGAT
TH2H2         TGTTCCGTAAAACAAGCAACACCAGGCGGCGTTGTCAAAAAGGGTGATGTAGTAAGAGCG GTTGTCGTTCGCACTAAACGTTCTATCCGCCGTAAAGACGGTTCCTACATCGGATTTGAT
EB4_G3        TGTTCCGTAAAACAAGCAACACCAGGCGGCGTTGTCAAAAAGGGTGATGTAGTAAGAGCG GTTGTCGTTCGCACTAAACGTTCTATCCGCCGTAAAGACGGTTCCTACATCGGATTTGAT
CICC_10580    TGTTCCGTAAAACAAGCAACACCAGGCGGCGTTGTCAAAAAGGGTGATGTAGTAAGAGCG GTTGTCGTTCGCACTAAACGTTCTATCCGCCGTAAAGACGGTTCCTACATCGGATTTGAT
JE201         TGTTCCGTAAAACAAGCAACACCAGGCGGCGTTGTCAAAAAGGGTGATGTAGTAAGAGCG GTTGTCGTTCGCACTAAACGTTCTATCCGCCGTAAAGACGGTTCCTACATCGGATTTGAT
DCB2          TGTTCCGTAAAACAAGCAACACCAGGCGGCGTTGTCAAAAAGGGTGATGTAGTAAGAGCG GTTGTCGTTCGCACTAAACGTTCTATCCGCCGTAAAGACGGTTCCTACATCGGATTTGAT
ZF129         TGTTCCGTAAAACAAGCAACACCAGGCGGCGTTGTCAAAAAGGGTGATGTAGTAAGAGCG GTTGTCGTTCGCACTAAACGTTCTATCCGCCGTAAAGACGGTTCCTACATCGGATTTGAT
KP10          TGTTCCGTAAAACAAGCAACACCAGGCGGCGTTGTCAAAAAGGGTGATGTAGTAAGAGCG GTTGTCGTTCGCACTAAACGTTCTATCCGCCGTAAAGACGGTTCCTACATCGGATTTGAT
IIF8SW        TGTTCCGTAAAACAAGCAACACCAGGCGGCGTTGTCAAAAAGGGTGATGTAGTAAGAGCG GTTGTCGTTCGCACTAAACGTTCTATCCGCCGTAAAGACGGTTCCTACATCGGATTTGAT
2A_2A         TGTTCCGTAAAACAAGCAACACCAGGCGGCGTTGTCAAAAAGGGTGATGTAGTAAGAGCG GTTGTCGTTCGCACTAAACGTTCTATCCGCCGTAAAGACGGTTCCTACATCGGATTTGAT
181MFCol51    TGTTCCGTAAAACAAGCAACACCAGGCGGCGTTGTCAAAAAGGGTGATGTAGTAAGAGCG GTTGTCGTTCGCACTAAACGTTCTATCCGCCGTAAAGACGGTTCCTACATCGGATTTGAT
EBL06         TGTTCCGTAAAACAAGCAACACCAGGCGGCGTTGTCAAAAAGGGTGATGTAGTAAGAGCG GTTGTCGTTCGCACTAAACGTTCTATCCGCCGTAAAGACGGTTCCTACATCGGATTTGAT
ZJ_9          TGTTCCGTAAAACAAGCAACACCAGGCGGCGTTGTCAAAAAGGGTGATGTAGTAAGAGCG GTTGTCGTTCGCACTAAACGTTCTATCCGCCGTAAAGACGGTTCCTACATCGGATTTGAT
SQR_21        TGTTCCGTAAAACAAGCAACACCAGGCGGCGTTGTCAAAAAGGGTGATGTAGTAAGAGCG GTTGTCGTTCGCACTAAACGTTCTATCCGCCGTAAAGACGGTTCCTACATCGGATTTGAT
CF05          TGTTCCGTAAAACAAGCAACACCAGGCGGCGTTGTCAAAAAGGGTGATGTAGTAAGAGCG GTTGTCGTTCGCACTAAACGTTCTATCCGCCGTAAAGACGGTTCCTACATCGGATTTGAT
KCCM_40454    TGTTCCGTAAAACAAGCAACACCAGGCGGCGTTGTCAAAAAGGGTGATGTAGTAAGAGCG GTTGTCGTTCGCACTAAACGTTCTATCCGCCGTAAAGACGGTTCCTACATCGGATTTGAT
1_43          TGTTCCGTAAAACAAGCAACACCAGGCGGCGTTGTCAAAAAGGGTGATGTAGTAAGAGCG GTTGTCGTTCGCACTAAACGTTCTATCCGCCGTAAAGACGGTTCCTACATCGGATTTGAT
YS18          TGTTCCGTAAAACAAGCAACACCAGGCGGCGTTGTCAAAAAGGGTGATGTAGTAAGAGCG GTTGTCGTTCGCACTAAACGTTCTATCCGCCGTAAAGACGGTTCCTACATCGGATTTGAT
              ************************************************************ ***** ***** ******************************** **************
```

```
Cluster 1 TGTTCCGTAAAACAAGCAACACCAGGCGGCGTTGTCAAAAAGGGTGATGTAGTAAGAGCGGTTGTTGTTCGT ACTAAACGTTCTATCCGCCGTAAAGACGGTTCT/CTACATCGGATTTGAC
Cluster 2 TGTTCCGTAAAACAAGCAACACCAGGCGGCGTTGTCAAAAAGGGTGATGTAGTAAGAGCGGTTGTTGTTCGT ACTAAACGTTCTATCCGCCGTAAAGACGGTTCCfTACATCGGATTTGAT
Cluster 3 TGTTCCGTAAAACAAGCAACACCAGGCGGCGTTGTCAAAAAGGGTGATGTAGTAAGAGCGGTTGTCGTTCGT/CACTAAACGTTCTATCCGCCGTAAAGACGGTTCC TACATCGGATTTGAT
Cluster 4 TGTTCCGTAAAACAAGCAACACCAGGCGGCGTTGTCAAAAAGGGTGATGTAGTAAGAGCGGTTGTCGTTCGC ACTAAACGTTCTATCCGCCGTAAAGACGGTTCC TACATCGGATTTGAT
```

```
ZF197          GAGAACGCAGCAGTGGTCGTTAAAGAAGACAAGAGCCCACGTGGTACTCGTATTTTCGGA CCAGTTGCTCGCGAACTTCGTGACAAAGACTTCATGAAAATCGTTTCCTTGGCTCCGGAA GTGATCTAA
ND24           GAGAACGCAGCAGTGGTCGTTAAAGAAGACAAGAGCCCACGTGGTACTCGTATTTTCGGA CCAGTTGCTCGCGAACTTCGTGACAAAGACTTCATGAAAATCGTTTCCTTGGCTCCGGAA GTGATCTAA
P.ottowii      GAGAACGCAGCAGTGGTCGTTAAAGAAGACAAGAGCCCACGTGGTACTCGTATTTTCGGA CCAGTTGCTCGCGAACTTCGTGACAAAGACTTCATGAAAATCGTTTCCTTGGCTCCGGAA GTGATCTAA
E681           GAGAACGCAGCAGTGGTCGTTAAAGAAGACAAGAGCCCACGTGGTACTCGTATTTTCGGA CCAGTTGCTCGCGAACTTCGTGACAAGGACTTCATGAAAATCGTTTCCTTGGCTCCGGAA GTGATCTAA
MES20          GAAAACGCAGCAGTGGTCGTTAAAGAAGACAAGAGCCCACGTGGTACTCGTATTTTCGGA CCAGTTGCTCGCGAACTTCGTGACAAGGACTTCATGAAAATCGTTTCCTTGGCTCCGGAA GTGATCTAA
MES17          GAAAACGCAGCAGTGGTCGTTAAAGAAGACAAGAGCCCACGTGGTACTCGTATTTTCGGA CCAGTTGCTCGCGAACTTCGTGACAAGGACTTCATGAAAATCGTTTCCTTGGCTCCGGAA GTGATCTAA
MES108         GAAAACGCAGCAGTGGTCGTTAAAGAAGACAAGAGCCCACGTGGTACTCGTATTTTCGGA CCAGTTGCTCGCGAACTTCGTGACAAGGACTTCATGAAAATCGTTTCCTTGGCTCCGGAA GTGATCTAA
CCI_25         GAAAACGCAGCAGTGGTCGTTAAAGAAGACAAGAGCCCACGTGGTACTCGTATTTTCGGA CCAGTTGCTCGCGAACTTCGTGACAAGGACTTCATGAAAATCGTTTCCTTGGCTCCGGAA GTGATCTAA
J              GAAAACGCAGCAGTGGTCGTTAAAGAAGACAAGAGCCCACGTGGTACTCGTATTTTCGGA CCAGTTGCTCGCGAACTTCGTGACAAGGACTTCATGAAAATCGTTTCCTTGGCTCCGGAA GTGATCTAA
YUPP_8         GAAAACGCAGCAGTGGTCGTTAAAGAAGACAAGAGCCCACGTGGTACTCGTATTTTCGGA CCAGTTGCTCGCGAACTTCGTGACAAGGACTTCATGAAAATCGTTTCCTTGGCTCCGGAA GTGATCTAA
YC0573         GAGAACGCAGCAGTGGTCGTTAAAGAAGACAAGAGCCCACGTGGTACTCGTATTTTCGGA CCAGTTGCTCGCGAACTTCGTGACAAGGACTTCATGAAAATTGTTTCCTTGGCTCCGGAA GTGATCTAA
LY214          GAGAACGCAGCAGTGGTCGTTAAAGAAGACAAGAGCCCACGTGGTACTCGTATTTTCGGA CCAGTTGCTCGCGAACTTCGTGACAAGGACTTCATGAAAATTGTTTCCTTGGCTCCGGAA GTGATCTAA
CR1            GAGAACGCAGCAGTGGTCGTTAAAGAAGACAAGAGCCCACGTGGTACTCGTATTTTCGGA CCAGTTGCTCGCGAACTTCGTGACAAGGACTTCATGAAAATCGTTTCCTTGGCTCCGGAA GTGATCTAA
K16            GAGAACGCAGCAGTGGTCGTTAAAGAAGACAAGAGCCCACGTGGTACTCGTATTTTCGGA CCAGTTGCTCGCGAACTTCGTGACAAGGACTTCATGAAAATCGTTTCCTTGGCTCCGGAA GTGATCTAA
CFSAN034343    GAGAACGCAGCAGTGGTCGTTAAAGAAGACAAGAGCCCACGTGGTACTCGTATTTTCGGA CCAGTTGCTCGCGAACTTCGTGACAAGGACTTCATGAAAATCGTTTCCTTGGCTCCGGAA GTGATCTAA
CFSAN034342    GAGAACGCAGCAGTGGTCGTTAAAGAAGACAAGAGCCCACGTGGTACTCGTATTTTCGGA CCAGTTGCTCGCGAACTTCGTGACAAGGACTTCATGAAAATCGTTTCCTTGGCTCCGGAA GTGATCTAA
ATCC_15970     GAGAACGCAGCAGTGGTCGTTAAAGAAGACAAGAGCCCACGTGGTACTCGTATTTTCGGA CCAGTTGCTCGCGAACTTCGTGACAAGGACTTCATGAAAATCGTTTCCTTGGCTCCGGAA GTGATCTAA
DSM_292        GAGAACGCAGCAGTGGTCGTTAAAGAAGACAAGAGCCCACGTGGTACTCGTATTTTCGGA CCAGTTGCTCGCGAACTTCGTGACAAGGACTTCATGAAAATCGTTTCCTTGGCTCCGGAA GTGATCTAA
WLY78          GAGAACGCAGCAGTGGTCGTTAAAGAAGACAAGAGCCCACGTGGTACTCGTATTTTCGGA CCAGTTGCTCGCGAACTTCGTGACAAGGACTTCATGAAAATCGTTTCCTTGGCTCCGGAA GTGATCTAA
DSM_365        GAGAACGCAGCAGTGGTCGTTAAAGAAGACAAGAGCCCACGTGGTACTCGTATTTTCGGA CCAGTTGCTCGCGAACTTCGTGACAAGGACTTCATGAAAATCGTTTCCTTGGCTCCGGAA GTGATCTAA
MES110         GAGAACGCAGCAGTGGTCGTTAAAGAAGACAAGAGCCCACGTGGTACTCGTATTTTCGGA CCAGTTGCTCGCGAACTTCGTGACAAGGACTTCATGAAAATCGTTTCCTTGGCTCCGGAA GTGATCTAA
LMG_27872      GAGAACGCAGCAGTGGTCGTTAAAGAAGACAAGAGCCCACGTGGTACTCGTATTTTCGGA CCAGTTGCTCGCGAACTTCGTGACAAGGACTTCATGAAAATCGTTTCCTTGGCTCCGGAA GTGATCTAA
CFSAN034341    GAGAACGCAGCAGTGGTCGTTAAAGAAGACAAGAGCCCACGTGGTACTCGTATTTTCGGA CCAGTTGCTCGCGAACTTCGTGACAAGGACTTCATGAAAATCGTTTCCTTGGCTCCGGAA GTGATCTAA
Hob6           GAGAACGCAGCAGTGGTCGTTAAAGAAGACAAGAGCCCACGTGGTACTCGTATTTTCGGA CCAGTTGCTCGCGAACTTCGTGACAAGGACTTCATGAAAATCGTTTCCTTGGCTCCGGAA GTGATCTAA
MES972         GAGAACGCAGCAGTGGTCGTTAAAGAAGACAAGAGCCCACGTGGTACTCGTATTTTCGGA CCAGTTGCTCGCGAACTTCGTGACAAGGACTTCATGAAAATCGTTTCCTTGGCTCCGGAA GTGATCTAA
YC0136         GAGAACGCAGCAGTGGTCGTTAAAGAAGACAAGAGCCCACGTGGTACTCGTATTTTCGGA CCAGTTGCTCGCGAACTTCGTGACAAGGACTTCATGAAAATCGTTTCCTTGGCTCCGGAA GTGATCTAA
P3             GAGAACGCAGCAGTGGTCGTTAAAGAAGACAAGAGCCCACGTGGTACTCGTATTTTCGGA CCAGTTGCTCGCGAACTTCGTGACAAGGACTTCATGAAAATCGTTTCCTTGGCTCCGGAA GTGATCTAA
Sb3            GAGAACGCAGCAGTGGTCGTTAAAGAAGACAAGAGCCCACGCGGTACTCGTATTTTCGGA CCAGTTGCTCGCGAACTTCGTGATAAAGACTTCATGAAAATCGTTTCCTTGGCTCCGGAA GTGATCTAA
CJX518         GAGAACGCAGCAGTGGTCGTTAAAGAAGACAAGAGCCCACGCGGTACTCGTATTTTCGGA CCAGTTGCTCGCGAACTTCGTGATAAAGACTTCATGAAAATCGTTTCCTTGGCTCCGGAA GTGATCTAA
KF_1           GAGAACGCAGCAGTGGTCGTTAAAGAAGACAAGAGCCCACGCGGTACTCGTATTTTCGGA CCAGTTGCTCGCGAACTTCGTGATAAAGACTTCATGAAAATCGTTTCCTTGGCTCCGGAA GTGATCTAA
ND25           GAGAACGCAGCAGTGGTCGTTAAAGAAGACAAGAGCCCACGCGGTACTCGTATTTTCGGA CCAGTTGCTCGCGAACTTCGTGATAAAGACTTCATGAAAATCGTTTCCTTGGCTCCGGAA GTGATCTAA
SC2            GAGAACGCAGCAGTGGTCGTTAAAGAAGACAAGAGCCCACGCGGTACTCGTATTTTCGGA CCAGTTGCTCGCGAACTTCGTGATAAAGACTTCATGAAAATCGTTTCCTTGGCTCCGGAA GTGATCTAA
M1             GAGAACGCAGCAGTGGTCGTTAAAGAAGACAAGAGCCCACGCGGTACTCGTATTTTCGGA CCAGTTGCTCGCGAACTTCGTGACAAGGACTTCATGAAAATCGTTTCCTTGGCTCCGGAA GTGATCTAA
S3             GAGAACGCAGCAGTTGTCGTTAAAGAAGACAAGAGCCCACGCGGTACTCGTATTTTCGGA CCAGTTGCTCGCGAACTTCGTGACAAGGACTTCATGAAAATCGTTTCCTTGGCTCCGGAA GTGATCTAA
HY96           GAGAACGCAGCAGTGGTCGTTAAAGAAGACAAGAGCCCACGCGGTACTCGTATTTTCGGA CCAGTTGCTCGCGAACTTCGTGACAAGGACTTTATGAAAATCGTTTCCTTGGCTCCGGAA GTGATCTAA
OSY_DF         GAGAACGCAGCAGTGGTCGTTAAAGAAGACAAGAGCCCACGCGGTACTCGTATTTTCGGA CCAGTTGCTCGCGAACTTCGTGACAAGGACTTCATGAAAATCGTTTCCTTGGCTCCGGAA GTGATCTAA
OSY_EC         GAGAACGCAGCAGTGGTCGTTAAAGAAGACAAGAGCCCACGCGGTACTCGTATTTTCGGA CCAGTTGCTCGCGAACTTCGTGACAAGGACTTCATGAAAATCGTTTCCTTGGCTCCGGAA GTGATCTAA
ATCC_842       GAGAACGCAGCAGTGGTCGTTAAAGAAGACAAGAGCCCACGCGGTACTCGTATTTTCGGA CCAGTTGCTCGCGAACTTCGTGACAAGGACTTCATGAAAATCGTTTCCTTGGCTCCGGAA GTGATCTAA
NRRL_B_30509   GAGAACGCAGCAGTGGTCGTTAAAGAAGACAAGAGCCCACGCGGTACTCGTATTTTCGGA CCAGTTGCTCGCGAACTTCGTGACAAGGACTTCATGAAAATCGTTTCCTTGGCTCCGGAA GTGATCTAA
NCTC7575       GAGAACGCAGCAGTGGTCGTTAAAGAAGACAAGAGCCCACGCGGTACTCGTATTTTCGGA CCAGTTGCTCGCGAACTTCGTGACAAGGACTTCATGAAAATCGTTTCCTTGGCTCCGGAA GTGATCTAA
MEZ6           GAGAACGCAGCAGTGGTCGTTAAAGAAGACAAGAGCCCACGCGGTACTCGTATTTTCGGA CCAGTTGCTCGCGAACTTCGTGACAAGGACTTCATGAAAATCGTTTCCTTGGCTCCGGAA GTGATCTAA
O_211          GAGAACGCAGCAGTGGTCGTTAAAGAAGACAAGAGCCCACGCGGTACTCGTATTTTCGGA CCAGTTGCTCGCGAACTTCGTGACAAGGACTTCATGAAAATCGTTTCCTTGGCTCCGGAA GTGATCTAA
HK4            GAGAACGCAGCAGTGGTCGTTAAAGAAGACAAGAGCCCACGCGGTACTCGTATTTTCGGA CCAGTTGCTCGCGAACTTCGTGACAAGGACTTCATGAAAATCGTTTCCTTGGCTCCGGAA GTGATCTAA
A18            GAGAACGCAGCAGTGGTCGTTAAAGAAGACAAGAGCCCACGCGGTACTCGTATTTTCGGA CCAGTTGCTCGCGAACTTCGTGACAAGGACTTCATGAAAATCGTTTCCTTGGCTCCGGAA GTGATCTAA
C12            GAGAACGCAGCAGTGGTCGTTAAAGAAGACAAGAGCCCACGCGGTACTCGTATTTTCGGA CCAGTTGCTCGCGAACTTCGTGACAAGGACTTCATGAAAATCGTTTCCTTGGCTCCGGAA GTGATCTAA
TH2H2          GAGAACGCAGCAGTGGTCGTTAAAGAAGACAAGAGCCCACGCGGTACTCGTATTTTCGGA CCAGTTGCTCGCGAACTTCGTGACAAGGACTTCATGAAAATCGTTTCCTTGGCTCCGGAA GTGATCTAA
EB4_G3         GAGAACGCAGCAGTGGTCGTTAAAGAAGACAAGAGCCCACGCGGTACTCGTATTTTCGGA CCAGTTGCTCGCGAACTTCGTGACAAGGACTTCATGAAAATCGTTTCCTTGGCTCCGGAA GTGATCTAA
CICC_10580     GAGAACGCAGCAGTGGTCGTTAAAGAAGACAAGAGCCCACGCGGTACTCGTATTTTCGGA CCAGTTGCTCGCGAACTTCGTGACAAGGACTTCATGAAAATCGTTTCCTTGGCTCCGGAA GTGATCTAA
JE201          GAGAACGCAGCAGTGGTCGTTAAAGAAGACAAGAGCCCACGCGGTACTCGTATTTTCGGA CCAGTTGCTCGCGAACTTCGTGACAAGGACTTCATGAAAATCGTTTCCTTGGCTCCGGAA GTGATCTAA
DCB2           GAGAACGCAGCAGTGGTCGTTAAAGAAGACAAGAGCCCACGCGGTACTCGTATTTTCGGA CCAGTTGCTCGCGAACTTCGTGACAAGGACTTCATGAAAATCGTTTCCTTGGCTCCGGAA GTGATCTAA
ZF129          GAGAACGCAGCAGTGGTCGTTAAAGAAGACAAGAGCCCACGCGGTACTCGTATTTTCGGA CCAGTTGCTCGCGAACTTCGTGACAAGGACTTCATGAAAATCGTTTCCTTGGCTCCGGAA GTGATCTAA
KP10           GAGAACGCAGCAGTGGTCGTTAAAGAAGACAAGAGCCCACGCGGTACTCGTATTTTCGGA CCAGTTGCTCGCGAACTTCGTGACAAGGACTTCATGAAAATCGTTTCCTTGGCTCCGGAA GTGATCTAA
IIF8SW         GAGAACGCAGCAGTGGTCGTTAAAGAAGACAAGAGCCCACGCGGTACTCGTATTTTCGGA CCAGTTGCTCGCGAACTTCGTGACAAGGACTTCATGAAAATCGTTTCCTTGGCTCCGGAA GTGATCTAA
2A_2A          GAGAACGCAGCAGTGGTCGTTAAAGAAGACAAGAGCCCACGCGGTACTCGTATTTTCGGA CCAGTTGCTCGCGAACTTCGTGACAAGGACTTCATGAAAATCGTTTCCTTGGCTCCGGAA GTGATCTAA
181MFCol51     GAGAACGCAGCAGTGGTCGTTAAAGAAGACAAGAGCCCACGCGGTACTCGTATTTTCGGA CCAGTTGCTCGCGAACTTCGTGACAAGGACTTCATGAAAATCGTTTCCTTGGCTCCGGAA GTGATCTAA
EBL06          GAGAACGCAGCAGTGGTCGTTAAAGAAGACAAGAGCCCACGCGGTACTCGTATTTTCGGA CCAGTTGCTCGCGAACTTCGTGACAAGGACTTCATGAAAATCGTTTCCTTGGCTCCGGAA GTGATCTAA
ZJ_9           GAGAACGCAGCAGTGGTCGTTAAAGAAGACAAGAGCCCACGCGGTACTCGTATTTTCGGA CCAGTTGCTCGCGAACTTCGTGACAAGGACTTCATGAAAATCGTTTCCTTGGCTCCGGAA GTGATCTAA
SQR_21         GAGAACGCAGCAGTGGTCGTTAAAGAAGACAAGAGCCCACGCGGTACTCGTATTTTCGGA CCAGTTGCTCGCGAACTTCGTGACAAGGACTTCATGAAAATCGTTTCCTTGGCTCCGGAA GTGATCTAA
CF05           GAGAACGCAGCAGTGGTCGTTAAAGAAGACAAGAGCCCACGCGGTACTCGTATTTTCGGA CCAGTTGCTCGCGAACTTCGTGACAAGGACTTCATGAAAATCGTTTCCTTGGCTCCGGAA GTGATCTAA
KCCM_40454     GAGAACGCAGCAGTGGTCGTTAAAGAAGACAAGAGCCCACGCGGTACTCGTATTTTCGGA CCAGTTGCTCGCGAACTTCGTGACAAGGACTTCATGAAAATCGTTTCCTTGGCTCCGGAA GTGATCTAA
1_43           GAGAACGCAGCAGTGGTCGTTAAAGAAGACAAGAGCCCACGCGGTACTCGTATTTTCGGA CCAGTTGCTCGCGAACTTCGTGACAAGGACTTCATGAAAATCGTTTCCTTGGCTCCGGAA GTGATCTAA
YS18           GAGAACGCAGCAGTGGTCGTTAAAGAAGACAAGAGCCCACGCGGTACTCGTATTTTCGGA CCAGTTGCTCGCGAACTTCGTGACAAGGACTTCATGAAAATCGTTTCCTTGGCCCCGGAA GTGATCTAA
               ** *********** ************************* ****************** ************************ ** ***** ******** *********** ****** *********
```

Cluster 1 GA$^{G/A}$AACGCAGCAGTG GTCGTTAAAGAAGACAAGAGCCCACG**T**GGTACTCGTATTTTCGGACCAGTTGCTCGCGAACTTCGTGA CAA GGACTT CATGAAAAT$^{C/T}$

Cluster 2 GAG AACGCAGCAGTG GTCGTTAAAGAAGACAAGAGCCCACG**T**GGTACTCGTATTTTCGGACCAGTTGCTCGCGAACTTCGTGA CAA AGACTT CATGAAAATC

Cluster 3 GAG AACGCAGCAGTG GTCGTTAAAGAAGACAAGAGCCCACG**C**GGTACTCGTATTTTCGGACCAGTTGCTCGCGAACTTCGTGA$^{C/T}$AA$^{G/A}$GACTT CATGAAAATC

Cluster 4 GAG AACGCAGCAGT$^{G/T}$GTCGTTAAAGAAGACAAGAGCCCACG**C**GGTACTCGTATTTTCGGACCAGTTGCTCGCGAACTTCGTGA CAA GGACTT$^{C/T}$ATGAAAATC

Figure S10. Multiple alignment of the *rplN* gene. The nucleotide sequences were obtained from the strains belonging to Cluster 1 (yellow), Cluster 2 (gray), Cluster 3 (green), and Cluster 4 (cyan). The red asterisks indicate conserved regions that can be used to design primers for gene amplification. The consensus sequences of each Cluster are displayed in the box below the alignment. Cluster-specific SNVs are highlighted using the same colors of the Clusters. SNVs common to Clusters 1 and 2 are indicated in red, while those common to Clusters 3 and 4 are in blue. No amino acid substitution is observed with protein L14 sequences.

rpmL

```
KF            ATGCCAAAAATGAAAACACATAGCAGCCTTAAAGGCCGCTTCAAAATTACCGGTACTGGTAAAGTAACGCGTTACAAAGCTTACAGAAACCACTTGCTTTCCCACAAATCCAAACGTGCA
Sb3           ATGCCAAAAATGAAAACACATAGCAGCCTTAAAGGCCGCTTCAAAATTACCGGTACTGGTAAAGTAACGCGTTACAAAGCTTACAGAAATCACTTGCTTTCCCACAAATCCAAACGTGCA
CJX518        ATGCCAAAAATGAAAACACATAGCAGCCTTAAAGGCCGCTTCAAAATTACCGGTACTGGTAAAGTAACGCGTTACAAAGCTTACAGAAATCACTTGCTTTCCCACAAATCCAAACGTGCA
1_43          ATGCCAAAAATGAAAACACATAGCAGCCTTAAAGGCCGCTTCAAAATTACCGGTACTGGTAAAGTAACGCGTTACAAAGCTTACAGAAATCACTTGCTTTCCCACAAATCCAAACGTGCA
ZF197         ATGCCAAAAATGAAAACACATAGCAGCCTTAAAGGCCGCTTCAAAATTACCGGTACTGGTAAAGTGACTCGTTACAAAGCTTACAGAAACCACTTGCTTTCCCACAAATCCAAACGTGCA
ND24          ATGCCAAAAATGAAAACACATAGCAGCCTTAAAGGCCGCTTCAAAATTACCGGTACTGGTAAAGTGACTCGTTACAAAGCTTACAGAAACCACTTGCTTTCCCACAAATCCAAACGTGCA
P.ottowii     ATGCCAAAAATGAAAACACATAGCAGCCTTAAAGGCCGCTTCAAAATTACCGGTACTGGTAAAGTGACTCGTTACAAAGCTTACAGAAACCACTTGCTTTCCCACAAATCCAAACGTGCA
181MFCol51    ATGCCAAAAATGAAAACACATAGCAGCCTTAAAGGCCGCTTCAAAATTACCGGTACTGGTAAAGTGACGCGTTACAAAGCTTACAGAAACCACTTGCTTTCCCACAAATCCAAACGTGCA
TH2H2         ATGCCAAAAATGAAAACACATAGCAGCCTTAAAGGCCGCTTCAAAATTACCGGTACTGGTAAAGTAACGCGTTACAAAGCTTACAGAAACCACTTGCTTTCCCACAAATCCAAACGTGCA
EB4_G3        ATGCCAAAAATGAAAACACATAGCAGCCTTAAAGGCCGCTTCAAAATTACCGGTACTGGTAAAGTAACGCGTTACAAAGCTTACAGAAACCACTTGCTTTCCCACAAATCCAAACGTGCA
DCB2          ATGCCAAAAATGAAAACACATAGCAGCCTTAAAGGCCGCTTCAAAATTACCGGTACTGGTAAAGTAACGCGTTACAAAGCTTACAGAAACCACTTGCTTTCCCACAAATCCAAACGTGCA
ZF129         ATGCCAAAAATGAAAACACATAGCAGCCTTAAAGGCCGCTTCAAAATTACCGGTACTGGTAAAGTAACGCGTTACAAAGCTTACAGAAACCACTTGCTTTCCCACAAATCCAAACGTGCA
KP10          ATGCCAAAAATGAAAACACATAGCAGCCTTAAAGGCCGCTTCAAAATTACCGGTACTGGTAAAGTAACGCGTTACAAAGCTTACAGAAACCACTTGCTTTCCCACAAATCCAAACGTGCA
S3            ATGCCAAAAATGAAAACACATAGCAGCCTTAAAGGCCGCTTCAAAATTACCGGTACTGGTAAAGTAACGCGTTACAAAGCTTACAGAAACCACTTGCTTTCCCACAAATCCAAACGTGCA
ND25          ATGCCAAAAATGAAAACACATAGCAGCCTTAAAGGCCGCTTCAAAATTACCGGTACTGGTAAAGTAACGCGTTACAAAGCTTACAGAAACCACTTGCTTTCCCACAAATCCAAACGTGCA
SC2           ATGCCAAAAATGAAAACACATAGCAGCCTTAAAGGCCGCTTCAAAATTACCGGTACTGGTAAAGTAACGCGTTACAAAGCTTACAGAAACCACTTGCTTTCCCACAAATCCAAACGTGCA
M1            ATGCCAAAAATGAAAACACATAGCAGCCTTAAAGGCCGCTTCAAAATTACCGGTACTGGTAAAGTAACGCGTTACAAAGCTTACAGAAACCACTTGCTTTCCCACAAATCCAAACGTGCA
HK4           ATGCCAAAAATGAAAACACATAGCAGCCTTAAAGGCCGCTTCAAAATTACCGGTACTGGTAAAGTAACGCGTTACAAAGCTTACAGAAACCATTTGCTTTCCCACAAATCCAAACGTGCA
CICC_10580    ATGCCAAAAATGAAAACACATAGCAGCCTTAAAGGCCGCTTCAAAATTACCGGTACTGGTAAAGTAACGCGTTACAAAGCTTACAGAAACCATTTGCTTTCCCACAAATCCAAACGTGCA
JE201         ATGCCAAAAATGAAAACACATAGCAGCCTTAAAGGCCGCTTCAAAATTACCGGTACTGGTAAAGTAACGCGTTACAAAGCTTACAGAAACCATTTGCTTTCCCACAAATCCAAACGTGCA
NRRL_B_30509  ATGCCAAAAATGAAAACACATAGCAGCCTTAAAGGCCGCTTCAAAATTACCGGTACTGGTAAAGTGACGCGTTACAAAGCTTACAGAAACCACTTGCTTTCCCACAAATCCAAACGTGCA
OSY_DF        ATGCCAAAAATGAAAACACATAGCAGCCTTAAAGGCCGCTTCAAAATTACCGGTACTGGTAAAGTAACGCGTTACAAAGCTTACAGAAACCACTTGCTTTCCCACAAATCCAAACGTGCA
OSY_EC        ATGCCAAAAATGAAAACACATAGCAGCCTTAAAGGCCGCTTCAAAATTACCGGTACTGGTAAAGTAACGCGTTACAAAGCTTACAGAAACCACTTGCTTTCCCACAAATCCAAACGTGCA
2A_2A         ATGCCAAAAATGAAAACACATAGCAGCCTTAAAGGCCGCTTCAAAATTACCGGTACTGGTAAAGTAACGCGTTACAAAGCTTACAGAAACCACTTGCTTTCCCACAAATCCAAACGTGCA
YS18          ATGCCAAAAATGAAAACACATAGCAGCCTTAAAGGCCGCTTCAAAATTACCGGTACTGGTAAAGTAACGCGTTACAAAGCTTACAGAAACCACTTGCTTTCCCACAAATCCAAACGTGCA
EBL06         ATGCCAAAAATGAAAACACATAGCAGCCTTAAAGGCCGCTTCAAAATTACCGGTACTGGTAAAGTAACGCGTTACAAAGCTTACAGAAACCACTTGCTTTCCCACAAATCCAAACGTGCA
HY96          ATGCCAAAAATGAAAACACATAGCAGCCTTAAAGGCCGCTTCAAAATTACCGGTACTGGTAAAGTAACGCGTTACAAAGCTTACAGAAACCACTTGCTTTCCCACAAATCCAAACGTGCA
ZJ_9          ATGCCAAAAATGAAAACACATAGCAGCCTTAAAGGCCGCTTCAAAATTACCGGTACTGGTAAAGTAACGCGTTACAAAGCTTACAGAAACCACTTGCTTTCCCACAAATCCAAACGTGCA
KCCM_40454    ATGCCAAAAATGAAAACACATAGCAGCCTTAAAGGCCGCTTCAAAATTACCGGTACTGGTAAAGTAACGCGTTACAAAGCTTACAGAAACCACTTGCTTTCCCACAAATCCAAACGTGCA
ATCC_842      ATGCCAAAAATGAAAACACATAGCAGCCTTAAAGGCCGCTTCAAAATTACCGGTACTGGTAAAGTGACGCGTTACAAAGCTTACAGAAACCACTTGCTTTCCCACAAATCCAAACGTGCA
NCTC7575      ATGCCAAAAATGAAAACACATAGCAGCCTTAAAGGCCGCTTCAAAATTACCGGTACTGGTAAAGTGACGCGTTACAAAGCTTACAGAAACCACTTGCTTTCCCACAAATCCAAACGTGCA
MEZ6          ATGCCAAAAATGAAAACACATAGCAGCCTTAAAGGCCGCTTCAAAATTACCGGTACTGGTAAAGTGACGCGTTACAAAGCTTACAGAAACCACTTGCTTTCCCACAAATCCAAACGTGCA
O_211         ATGCCAAAAATGAAAACACATAGCAGCCTTAAAGGCCGCTTCAAAATTACCGGTACTGGTAAAGTGACGCGTTACAAAGCTTACAGAAACCACTTGCTTTCCCACAAATCCAAACGTGCA
A18           ATGCCAAAAATGAAAACACATAGCAGCCTTAAAGGCCGCTTCAAAATTACCGGTACTGGTAAAGTGACGCGTTACAAAGCTTACAGAAACCACTTGCTTTCCCACAAATCCAAACGTGCA
C12           ATGCCAAAAATGAAAACACATAGCAGCCTTAAAGGCCGCTTCAAAATTACCGGTACTGGTAAAGTGACGCGTTACAAAGCTTACAGAAACCACTTGCTTTCCCACAAATCCAAACGTGCA
IIF8SW        ATGCCAAAAATGAAAACACATAGCAGCCTTAAAGGCCGCTTCAAAATTACCGGTACTGGTAAAGTGACGCGTTACAAAGCTTACAGAAACCACTTGCTTTCCCACAAATCCAAACGTGCA
SQR_21        ATGCCAAAAATGAAAACACATAGCAGCCTTAAAGGCCGCTTCAAAATTACCGGTACTGGTAAAGTGACGCGTTACAAAGCTTACAGAAACCACTTGCTTTCCCACAAATCCAAACGTGCA
CF05          ATGCCAAAAATGAAAACACATAGCAGCCTTAAAGGCCGCTTCAAAATTACCGGTACTGGTAAAGTGACGCGTTACAAAGCTTACAGAAACCACTTGCTTTCCCACAAATCCAAACGTGCA
MES972        ATGCCTAAAATGAAAACACATAGCAGCCTTAAAGGCCGCTTCAAGATTACCGGTACTGGTAAAGTAATGCGTTACAAAGCCTACAAAAACCACTTGCTTTCCCACAAATCCAAACGTGCA
WLY78         ATGCCTAAAATGAAAACACATAGCAGCCTTAAAGGCCGCTTCAAAATTACCGGTACTGGTAAAGTAATGCGTTACAAAGCTTACAAAAACCACTTGCTTTCCCACAAATCCAAACGTGCA
DSM_365       ATGCCTAAAATGAAAACACATAGCAGCCTTAAAGGCCGCTTCAAAATTACCGGTACTGGTAAAGTAATGCGTTACAAAGCTTACAAAAACCACTTGCTTTCCCACAAATCCAAACGTGCA
LMG_27872     ATGCCTAAAATGAAAACACATAGCAGCCTTAAAGGCCGCTTCAAAATTACCGGTACTGGTAAAGTAATGCGTTACAAAGCTTACAAAAACCACTTGCTTTCCCACAAATCCAAACGTGCA
CFSAN03434    ATGCCTAAAATGAAAACACATAGCAGCCTTAAAGGCCGCTTCAAAATTACCGGTACTGGTAAAGTAATGCGTTACAAAGCTTACAAAAACCACTTGCTTTCCCACAAATCCAAACGTGCA
Hob6          ATGCCTAAAATGAAAACACATAGCAGCCTTAAAGGCCGCTTCAAAATTACCGGTACTGGTAAAGTAATGCGTTACAAAGCTTACAAAAACCACTTGCTTTCCCACAAATCCAAACGTGCA
MES110        ATGCCTAAAATGAAAACACATAGCAGCCTTAAAGGCCGCTTCAAAATTACCGGTACTGGTAAAGTAATGCGTTACAAAGCTTACAAAAATCACTTGCTTTCCCACAAATCCAAACGTGCA
YC0136        ATGCCTAAAATGAAAACACATAGCAGCCTTAAAGGCCGCTTCAAAATTACCGGTACTGGTAAAGTAATGCGTTACAAAGCTTACAAAAACCATTTGCTTTCCCACAAATCCAAACGTGCG
YC0573        ATGCCTAAAATGAAAACACATAGCAGCCTTAAAGGCCGCTTCAAAATTACCGGTACTGGTAAAGTAATGCGTTACAAAGCTTACAAAAACCATTTGCTTTCCCACAAATCCAAACGTGCA
LY214         ATGCCTAAAATGAAAACACATAGCAGCCTTAAAGGCCGCTTCAAAATTACCGGTACTGGTAAAGTAATGCGTTACAAAGCTTACAAAAACCATTTGCTTTCCCACAAATCCAAACGTGCA
CR1           ATGCCTAAAATGAAAACACATAGCAGCCTTAAAGGCCGCTTCAAAATTACCGGTACTGGTAAAGTAATGCGTTACAAAGCTTACAAAAACCATTTGCTTTCCCACAAATCCAAACGTGCA
K16           ATGCCTAAAATGAAAACACATAGCAGCCTTAAAGGCCGCTTCAAAATTACCGGTACTGGTAAAGTAATGCGTTACAAAGCTTACAAAAACCATTTGCTTTCCCACAAATCCAAACGTGCA
CFSAN034343   ATGCCTAAAATGAAAACACATAGCAGCCTTAAAGGCCGCTTCAAAATTACCGGTACTGGTAAAGTAATGCGTTACAAAGCTTACAAAAACCATTTGCTTTCCCACAAATCCAAACGTGCA
CFSAN034342   ATGCCTAAAATGAAAACACATAGCAGCCTTAAAGGCCGCTTCAAAATTACCGGTACTGGTAAAGTAATGCGTTACAAAGCTTACAAAAACCATTTGCTTTCCCACAAATCCAAACGTGCA
ATCC_15970    ATGCCTAAAATGAAAACACATAGCAGCCTTAAAGGCCGCTTCAAAATTACCGGTACTGGTAAAGTAATGCGTTACAAAGCTTACAAAAACCATTTGCTTTCCCACAAATCCAAACGTGCA
DSM_292       ATGCCTAAAATGAAAACACATAGCAGCCTTAAAGGCCGCTTCAAAATTACCGGTACTGGTAAAGTAATGCGTTACAAAGCTTACAAAAACCATTTGCTTTCCCACAAATCCAAACGTGCA
E681          ATGCCTAAAATGAAAACACATAGCAGCCTTAAAGGCCGCTTCAAAATTACCGGTACTGGTAAAGTAATGCGTTACAAAGCTTACAAAAACCATTTGCTTTCCCACAAATCCAAACGTGCA
P3            ATGCCTAAAATGAAAACACATAGCAGCCTTAAAGGCCGCTTCAAAATTACCGGTACTGGTAAAGTAATGCGTTACAAAGCTTACAAAAACCATTTGCTTTCCCACAAATCCAAACGTGCA
MES20         ATGCCTAAAATGAAAACACATAGCAGCCTTAAAGGCCGCTTCAAAATTACCGGTACTGGTAAAGTAATGCGTTACAAAGCTTACAAAAACCATTTGCTTTCCCACAAATCCAAACGTGCA
MES17         ATGCCTAAAATGAAAACACATAGCAGCCTTAAAGGCCGCTTCAAAATTACCGGTACTGGTAAAGTAATGCGTTACAAAGCTTACAAAAACCATTTGCTTTCCCACAAATCCAAACGTGCA
MES108        ATGCCTAAAATGAAAACACATAGCAGCCTTAAAGGCCGCTTCAAAATTACCGGTACTGGTAAAGTAATGCGTTACAAAGCTTACAAAAACCATTTGCTTTCCCACAAATCCAAACGTGCA
CCI_25        ATGCCTAAAATGAAAACACATAGCAGCCTTAAAGGCCGCTTCAAAATTACCGGTACTGGTAAAGTAATGCGTTACAAAGCTTACAAAAACCATTTGCTTTCCCACAAATCCAAACGTGCA
J             ATGCCTAAAATGAAAACACATAGCAGCCTTAAAGGCCGCTTCAAAATTACCGGTACTGGTAAAGTAATGCGTTACAAAGCTTACAAAAACCATTTGCTTTCCCACAAATCCAAACGTGCA
YUPP_8        ATGCCTAAAATGAAAACACATAGCAGCCTTAAAGGCCGCTTCAAAATTACCGGTACTGGTAAAGTAATGCGTTACAAAGCTTACAAAAACCATTTGCTTTCCCACAAATCCAAACGTGCA
              ***** ************************************** ******************** *  *********** **** *** ** **************************
```

```
Cluster 1    CAA^A/G ATTACCGGTACTGGTAAAGTA ATGCGTTACAAAGC^T/C TACAAAAA^C/T CA^T/C TTGCTTTCCCACAAATCCAAACGTGC^A/G
Cluster 2    CAAA ATTACCGGTACTGGTAAAGTG ACTCGTTACAAAGCT TACAGAAA C CAC TTGCTTTCCCACAAATCCAAACGTGCA
Cluster 3    CAAA ATTACCGGTACTGGTAAAGTA ACGCGTTACAAAGCT TACAGAAA^C/T CAC TTGCTTTCCCACAAATCCAAACGTGCA
Cluster 4    CAAA ATTACCGGTACTGGTAAAGT^A/G ACGCGTTACAAAGCT TACAGAAA C CA^T/C TTGCTTTCCCACAAATCCAAACGTGCA
```

```
KF             AAACGCGTTCTGGGTACTAACCCTGAGATGGCACCTGGAGACGTTAGACGTCTGAAACAA GGTCTTGCTAACCTGAAATAG
Sb3            AAACGCGTTCTGGGTACTAACCCTGAGATGGCACCTGGGGACGTTAGACGTCTGAAACAA GGTCTTGCTAACCTGAAATAG
CJX518         AAACGCGTTCTGGGTACTAACCCTGAGATGGCACCTGGGGACGTTAGACGTCTGAAACAA GGTCTTGCTAACCTGAAATAG
1_43           AAACGCGTTCTGGGTACTAACCCTGAGATGGCACCTGGGGACGTTAGACGTCTGAAACAA GGTCTTGCTAACCTGAAATAG
ZF197          AAACGCGTTCTGGGTACTAACCCTGAGATGGCACCTGGGGACGTTAGACGTTTGAAACAA GGTCTTGCTAACCTGAAATAG
ND24           AAACGCGTTCTGGGTACTAACCCTGAGATGGCACCTGGGGACGTTAGACGTTTGAAACAA GGTCTTGCTAACCTGAAATAG
P.ottowii      AAACGCGTTCTGGGTACTAACCCTGAGATGGCACCTGGGGACGTTAGACGTTTGAAACAA GGTCTTGCTAACCTGAAATAG
181MFCol51     AAACGCGTTTTGGGTACTAACCCTGAGATGGCACCTGGGGACGTTAGACGTTTGAAACAA GGTCTTGCTAACCTGAAATAG
TH2H2          AAACGCGTTCTGGGTACTAACCCTGAGATGGCACCTGGGGACGTTAGACGTCTGAAACAA GGTCTTGCTAACCTGAAATAG
EB4_G3         AAACGCGTTCTGGGTACTAACCCTGAGATGGCACCTGGGGACGTTAGACGTCTGAAACAA GGTCTTGCTAACCTGAAATAG
DCB2           AAACGCGTTCTGGGTACTAACCCTGAGATGGCACCTGGGGACGTTAGACGTCTGAAACAA GGTCTTGCTAACCTGAAATAG
ZF129          AAACGCGTTCTGGGTACTAACCCTGAGATGGCACCTGGGGACGTTAGACGTCTGAAACAA GGTCTTGCTAACCTGAAATAG
KP10           AAACGCGTTCTGGGTACTAACCCTGAGATGGCACCTGGGGACGTTAGACGTCTGAAACAA GGTCTTGCTAACCTGAAATAG
S3             AAACGCGTTCTGGGTACTAACCCTGAGATGGCACCTGGGGACGTTAGACGTCTGAAACAA GGTCTTGCTAACCTGAAATAG
ND25           AAACGCGTTCTGGGTACTAACCCTGAGATGGCACCTGGGGACGTTAGACGTCTGAAACAA GGTCTTGCTAACCTGAAATAG
SC2            AAACGCGTTCTGGGTACTAACCCTGAGATGGCACCTGGGGACGTTAGACGTCTGAAACAA GGTCTTGCTAACCTGAAATAG
M1             AAACGCGTTCTGGGTACTAACCCTGAGATGGCACCTGGGGACGTTAGACGTCTGAAACAA GGTCTTGCTAACCTGAAATAG
HK4            AAACGCGTTCTGGGTACTAACCCTGAGATGGCACCTGGGGACGTTAGACGTTTGAAACAA GGTCTTGCTAACCTGAAATAG
CICC_10580     AAACGCGTTCTGGGTACTAACCCTGAGATGGCACCTGGGGACGTTAGACGTTTGAAACAA GGTCTTGCTAACCTGAAATAG
JE201          AAACGCGTTCTGGGTACTAACCCTGAGATGGCACCTGGGGACGTTAGACGTTTGAAACAA GGTCTTGCTAACCTGAAATAG
NRRL_B_30509   AAACGCGTTCTGGGTACTAACCCTGAGATGGCACCTGGGGACGTTAGACGTCTGAAACAA GGTCTTGCTAACCTGAAATAG
OSY_DF         AAACGCGTTCTGGGTACTAACCCTGAGATGGCACCTGGGGACGTTAGACGTTTGAAACAA GGTCTTGCTAACCTGAAATAG
OSY_EC         AAACGCGTTCTGGGTACTAACCCTGAGATGGCACCTGGGGACGTTAGACGTTTGAAACAA GGTCTTGCTAACCTGAAATAG
2A_2A          AAACGCGTTCTGGGTACTAACCCTGAGATGGCACCTGGGGACGTTAGACGTTTGAAACAA GGTCTTGCTAACCTGAAATAG
YS18           AAACGCGTTCTGGGTACTAACCCTGAGATGGCACCTGGGGACGTTAGACGTTTGAAACAA GGTCTTGCTAACCTGAAATAG
EBL06          AAACGCGTTCTGGGTACTAACCCTGAGATGGCACCTGGGGACGTTAGACGTTTGAAACAA GGTCTTGCTAACCTGAAATAG
HY96           AAACGCGTTCTGGGTACTAACCCTGAGATGGCACCTGGGGACGTTAGACGTTTGAAACAA GGTCTTGCTAACCTGAAATAG
ZJ_9           AAACGCGTTCTGGGTACTAACCCTGAGATGGCACCTGGGGACGTTAGACGTTTGAAACAA GGTCTTGCTAACCTGAAATAG
KCCM_40454     AAACGCGTTCTGGGTACTAACCCTGAGATGGCACCTGGGGACGTTAGACGTTTGAAACAA GGTCTTGCTAACCTGAAATAG
ATCC_842       AAACGCGTTCTGGGTACTAACCCTGAGATGGCACCTGGGGACGTTAGACGTTTGAAACAA GGTCTTGCTAACCTGAAATAG
NCTC7575       AAACGCGTTCTGGGTACTAACCCTGAGATGGCACCTGGGGACGTTAGACGTTTGAAACAA GGTCTTGCTAACCTGAAATAG
MEZ6           AAACGCGTTCTGGGTACTAACCCTGAGATGGCACCTGGGGACGTTAGACGTTTGAAACAA GGTCTTGCTAACCTGAAATAG
O_211          AAACGCGTTCTGGGTACTAACCCTGAGATGGCACCTGGGGACGTTAGACGTTTGAAACAA GGTCTTGCTAACCTGAAATAG
A18            AAACGCGTTCTGGGTACTAACCCTGAGATGGCACCTGGGGACGTTAGACGTTTGAAACAA GGTCTTGCTAACCTGAAATAG
C12            AAACGCGTTCTGGGTACTAACCCTGAGATGGCACCTGGGGACGTTAGACGTTTGAAACAA GGTCTTGCTAACCTGAAATAG
IIF8SW         AAACGCGTTCTGGGTACTAACCCTGAGATGGCACCTGGGGACGTTAGACGTTTGAAACAA GGTCTTGCTAACCTGAAATAG
SQR_21         AAACGCGTTCTGGGTACTAACCCTGAGATGGCACCTGGGGACGTTAGACGTTTGAAACAA GGTCTTGCTAACCTGAAATAG
CF05           AAACGCGTTCTGGGTACTAACCCTGAGATGGCACCTGGGGACGTTAGACGTTTGAAACAA GGTCTTGCTAACCTGAAATAG
MES972         AAACGCGTTCTGGGTACTAACCCTGAGATGGCACCTGGGGACGTTAGACGTCTGAAACAA GGTCTTGCTAACCTCAAATAG
WLY78          AAACGCGTTCTGGGTACTAACCCTGAGATGGCACCTGGGGACGTTAAGCGTCTGAAACAA GGTCTTGCTAACCTGAAATAG
DSM_365        AAACGCGTTCTGGGTACTAACCCTGAGATGGCACCTGGGGACGTTAAGCGTCTGAAACAA GGTCTTGCTAACCTGAAATAG
LMG_27872      AAACGCGTTCTGGGTACTAACCCTGAGATGGCACCTGGGGACGTTAAGCGTCTGAAACAA GGTCTTGCTAACCTGAAATAG
CFSAN03434     AAACGCGTTCTGGGTACTAACCCTGAGATGGCACCTGGGGACGTTAAGCGTCTGAAACAA GGTCTTGCTAACCTGAAATAG
Hob6           AAACGCGTTCTGGGTACTAACCCTGAGATGGCACCTGGGGACGTTAAGCGTCTGAAACAA GGTCTTGCTAACCTGAAATAG
MES110         AAACGCGTTCTGGGTACTAACCCTGAGATGGCACCTGGGGACGTTAAGCGTCTGAAACAA GGTCTTGCTAACCTGAAATAG
YC0136         AAACGCGTTCTGGGTACTAACCCTGAGATGGCACCTGGGGACGTTAAACGTCTGAAACAA GGTCTTGCTAACCTGAAATAG
YC0573         AAACGCGTTCTGGGTACTAACCCTGAGATGGCACCTGGGGACGTTAAACGTCTGAAACAA GGTCTTGCTAACCTGAAATAG
LY214          AAACGCGTTCTGGGTACTAACCCTGAGATGGCACCTGGGGACGTTAAACGTCTGAAACAA GGTCTTGCTAACCTGAAATAG
CR1            AAACGCGTTCTGGGTACTAACCCTGAGATGGCACCTGGGGACGTTAAACGTCTGAAACAA GGTCTTGCTAACCTGAAATAG
K16            AAACGCGTTCTGGGTACTAACCCTGAGATGGCACCTGGGGACGTTAAACGTCTGAAACAA GGTCTTGCTAACCTGAAATAG
CFSAN034343    AAACGCGTTCTGGGTACTAACCCTGAGATGGCACCTGGGGACGTTAAACGTCTGAAACAA GGTCTTGCTAACCTGAAATAG
CFSAN034342    AAACGCGTTCTGGGTACTAACCCTGAGATGGCACCTGGGGACGTTAAACGTCTGAAACAA GGTCTTGCTAACCTGAAATAG
ATCC_15970     AAACGCGTTCTGGGTACTAACCCTGAGATGGCACCTGGGGACGTTAAACGTCTGAAACAA GGTCTTGCTAACCTGAAATAG
DSM_292        AAACGCGTTCTGGGTACTAACCCTGAGATGGCACCTGGGGACGTTAAACGTCTGAAACAA GGTCTTGCTAACCTGAAATAG
E681           AAACGCGTTCTGGGTACTAACCCTGAGATGGCACCTGGGGACGTTAAACGTCTGAAACAA GGTCTTGCTAACCTGAAATAG
P3             AAACGCGTTCTGGGTACTAACCCTGAGATGGCACCTGGGGACGTTAAACGTCTGAAACAA GGTCTTGCTAACCTGAAATAG
MES20          AAACGCGTTCTGGGTACTAACCCTGAGATGGCACCTGGGGACGTTAAGCGTCTGAAACAA GGTCTTGCTAACCTGAAATAG
MES17          AAACGCGTTCTGGGTACTAACCCTGAGATGGCACCTGGGGACGTTAAGCGTCTGAAACAA GGTCTTGCTAACCTGAAATAG
MES108         AAACGCGTTCTGGGTACTAACCCTGAGATGGCACCTGGGGACGTTAAGCGTCTGAAACAA GGTCTTGCTAACCTGAAATAG
CCI_25         AAACGCGTTCTGGGTACTAACCCTGAGATGGCACCTGGGGACGTTAAGCGTCTGAAACAA GGTCTTGCTAACCTGAAATAG
J              AAACGCGTTCTGGGTACTAACCCTGAGATGGCACCTGGGGACGTTAAGCGTCTGAAACAA GGTCTTGCTAACCTGAAATAG
YUPP_8         AAACGCGTTCTGGGTACTAACCCTGAGATGGCACCTGGGGACGTTAAGCGTCTGAAACAA GGTCTTGCTAACCTGAAATAG
               *********  ***************************  *******   ***  ********  **************  ******
```

```
Cluster 1      AAACGCGTTC TGGGTACTAACCCTGAGATGGCACCTGGG GACGTTAA/GA/GCGTC TGAAACAA
Cluster 2      AAACGCGTTC TGGGTACTAACCCTGAGATGGCACCTGGG GACGTTAG A CGTT TGAAACAA
Cluster 3      AAACGCGTTC TGGGTACTAACCCTGAGATGGCACCTGGG/AGACGTTAG A CGTC TGAAACAA
Cluster 4      AAACGCGTTC/TTGGGTACTAACCCTGAGATGGCACCTGGG GACGTTAG A CGTC/TTGAAACAA
```

Figure S11. **Multiple alignment of the *rpmL* gene.** The nucleotide sequences were obtained from the strains belonging to Cluster 1 (yellow), Cluster 2 (gray), Cluster 3 (green), and Cluster 4 (cyan). The red asterisks indicate conserved regions that can be used to design primers for gene amplification. The consensus sequences of each Cluster are displayed in the box below the alignment. Cluster-specific SNVs are highlighted using the same colors of the Clusters. SNVs common to Clusters 3 and 4 are indicated in blue.

Ribosomal protein L35

```
OSY_DF          MPKMKTHSSLKGRFKITGTGKVTRYKAYRNHLLSHKSKRAKRVLGTNPEMAPGDVRRLKQ GLANLK
OSY_EC          MPKMKTHSSLKGRFKITGTGKVTRYKAYRNHLLSHKSKRAKRVLGTNPEMAPGDVRRLKQ GLANLK
ATCC_842        MPKMKTHSSLKGRFKITGTGKVTRYKAYRNHLLSHKSKRAKRVLGTNPEMAPGDVRRLKQ GLANLK
NRRL_B_30509    MPKMKTHSSLKGRFKITGTGKVTRYKAYRNHLLSHKSKRAKRVLGTNPEMAPGDVRRLKQ GLANLK
NCTC7575        MPKMKTHSSLKGRFKITGTGKVTRYKAYRNHLLSHKSKRAKRVLGTNPEMAPGDVRRLKQ GLANLK
MEZ6            MPKMKTHSSLKGRFKITGTGKVTRYKAYRNHLLSHKSKRAKRVLGTNPEMAPGDVRRLKQ GLANLK
O_211           MPKMKTHSSLKGRFKITGTGKVTRYKAYRNHLLSHKSKRAKRVLGTNPEMAPGDVRRLKQ GLANLK
HK4             MPKMKTHSSLKGRFKITGTGKVTRYKAYRNHLLSHKSKRAKRVLGTNPEMAPGDVRRLKQ GLANLK
A18             MPKMKTHSSLKGRFKITGTGKVTRYKAYRNHLLSHKSKRAKRVLGTNPEMAPGDVRRLKQ GLANLK
C12             MPKMKTHSSLKGRFKITGTGKVTRYKAYRNHLLSHKSKRAKRVLGTNPEMAPGDVRRLKQ GLANLK
TH2H2           MPKMKTHSSLKGRFKITGTGKVTRYKAYRNHLLSHKSKRAKRVLGTNPEMAPGDVRRLKQ GLANLK
EB4_G3          MPKMKTHSSLKGRFKITGTGKVTRYKAYRNHLLSHKSKRAKRVLGTNPEMAPGDVRRLKQ GLANLK
CICC_10580      MPKMKTHSSLKGRFKITGTGKVTRYKAYRNHLLSHKSKRAKRVLGTNPEMAPGDVRRLKQ GLANLK
JE201           MPKMKTHSSLKGRFKITGTGKVTRYKAYRNHLLSHKSKRAKRVLGTNPEMAPGDVRRLKQ GLANLK
DCB2            MPKMKTHSSLKGRFKITGTGKVTRYKAYRNHLLSHKSKRAKRVLGTNPEMAPGDVRRLKQ GLANLK
ZF129           MPKMKTHSSLKGRFKITGTGKVTRYKAYRNHLLSHKSKRAKRVLGTNPEMAPGDVRRLKQ GLANLK
KP10            MPKMKTHSSLKGRFKITGTGKVTRYKAYRNHLLSHKSKRAKRVLGTNPEMAPGDVRRLKQ GLANLK
IIF8SW          MPKMKTHSSLKGRFKITGTGKVTRYKAYRNHLLSHKSKRAKRVLGTNPEMAPGDVRRLKQ GLANLK
2A_2A           MPKMKTHSSLKGRFKITGTGKVTRYKAYRNHLLSHKSKRAKRVLGTNPEMAPGDVRRLKQ GLANLK
YS18            MPKMKTHSSLKGRFKITGTGKVTRYKAYRNHLLSHKSKRAKRVLGTNPEMAPGDVRRLKQ GLANLK
181MFCol51      MPKMKTHSSLKGRFKITGTGKVTRYKAYRNHLLSHKSKRAKRVLGTNPEMAPGDVRRLKQ GLANLK
EBL06           MPKMKTHSSLKGRFKITGTGKVTRYKAYRNHLLSHKSKRAKRVLGTNPEMAPGDVRRLKQ GLANLK
HY96            MPKMKTHSSLKGRFKITGTGKVTRYKAYRNHLLSHKSKRAKRVLGTNPEMAPGDVRRLKQ GLANLK
ZJ_9            MPKMKTHSSLKGRFKITGTGKVTRYKAYRNHLLSHKSKRAKRVLGTNPEMAPGDVRRLKQ GLANLK
SQR_21          MPKMKTHSSLKGRFKITGTGKVTRYKAYRNHLLSHKSKRAKRVLGTNPEMAPGDVRRLKQ GLANLK
CF05            MPKMKTHSSLKGRFKITGTGKVTRYKAYRNHLLSHKSKRAKRVLGTNPEMAPGDVRRLKQ GLANLK
KCCM_40454      MPKMKTHSSLKGRFKITGTGKVTRYKAYRNHLLSHKSKRAKRVLGTNPEMAPGDVRRLKQ GLANLK
S3              MPKMKTHSSLKGRFKITGTGKVTRYKAYRNHLLSHKSKRAKRVLGTNPEMAPGDVRRLKQ GLANLK
ZF197           MPKMKTHSSLKGRFKITGTGKVTRYKAYRNHLLSHKSKRAKRVLGTNPEMAPGDVRRLKQ GLANLK
ND24            MPKMKTHSSLKGRFKITGTGKVTRYKAYRNHLLSHKSKRAKRVLGTNPEMAPGDVRRLKQ GLANLK
P.ottowii       MPKMKTHSSLKGRFKITGTGKVTRYKAYRNHLLSHKSKRAKRVLGTNPEMAPGDVRRLKQ GLANLK
Sb3             MPKMKTHSSLKGRFKITGTGKVTRYKAYRNHLLSHKSKRAKRVLGTNPEMAPGDVRRLKQ GLANLK
CJX518          MPKMKTHSSLKGRFKITGTGKVTRYKAYRNHLLSHKSKRAKRVLGTNPEMAPGDVRRLKQ GLANLK
KF_1            MPKMKTHSSLKGRFKITGTGKVTRYKAYRNHLLSHKSKRAKRVLGTNPEMAPGDVRRLKQ GLANLK
ND25            MPKMKTHSSLKGRFKITGTGKVTRYKAYRNHLLSHKSKRAKRVLGTNPEMAPGDVRRLKQ GLANLK
1_43            MPKMKTHSSLKGRFKITGTGKVTRYKAYRNHLLSHKSKRAKRVLGTNPEMAPGDVRRLKQ GLANLK
SC2             MPKMKTHSSLKGRFKITGTGKVTRYKAYRNHLLSHKSKRAKRVLGTNPEMAPGDVRRLKQ GLANLK
M1              MPKMKTHSSLKGRFKITGTGKVTRYKAYRNHLLSHKSKRAKRVLGTNPEMAPGDVRRLKQ GLANLK
YC0573          MPKMKTHSSLKGRFKITGTGKVMRYKAYKNHLLSHKSKRAKRVLGTNPEMAPGDVKRLKQ GLANLK
LY214           MPKMKTHSSLKGRFKITGTGKVMRYKAYKNHLLSHKSKRAKRVLGTNPEMAPGDVKRLKQ GLANLK
CR1             MPKMKTHSSLKGRFKITGTGKVMRYKAYKNHLLSHKSKRAKRVLGTNPEMAPGDVKRLKQ GLANLK
K16             MPKMKTHSSLKGRFKITGTGKVMRYKAYKNHLLSHKSKRAKRVLGTNPEMAPGDVKRLKQ GLANLK
CFSAN034343     MPKMKTHSSLKGRFKITGTGKVMRYKAYKNHLLSHKSKRAKRVLGTNPEMAPGDVKRLKQ GLANLK
CFSAN034342     MPKMKTHSSLKGRFKITGTGKVMRYKAYKNHLLSHKSKRAKRVLGTNPEMAPGDVKRLKQ GLANLK
ATCC_15970      MPKMKTHSSLKGRFKITGTGKVMRYKAYKNHLLSHKSKRAKRVLGTNPEMAPGDVKRLKQ GLANLK
DSM_292         MPKMKTHSSLKGRFKITGTGKVMRYKAYKNHLLSHKSKRAKRVLGTNPEMAPGDVKRLKQ GLANLK
WLY78           MPKMKTHSSLKGRFKITGTGKVMRYKAYKNHLLSHKSKRAKRVLGTNPEMAPGDVKRLKQ GLANLK
DSM_365         MPKMKTHSSLKGRFKITGTGKVMRYKAYKNHLLSHKSKRAKRVLGTNPEMAPGDVKRLKQ GLANLK
LMG_27872       MPKMKTHSSLKGRFKITGTGKVMRYKAYKNHLLSHKSKRAKRVLGTNPEMAPGDVKRLKQ GLANLK
CFSAN034341     MPKMKTHSSLKGRFKITGTGKVMRYKAYKNHLLSHKSKRAKRVLGTNPEMAPGDVKRLKQ GLANLK
Hob6            MPKMKTHSSLKGRFKITGTGKVMRYKAYKNHLLSHKSKRAKRVLGTNPEMAPGDVKRLKQ GLANLK
MES110          MPKMKTHSSLKGRFKITGTGKVMRYKAYKNHLLSHKSKRAKRVLGTNPEMAPGDVKRLKQ GLANLK
MES20           MPKMKTHSSLKGRFKITGTGKVMRYKAYKNHLLSHKSKRAKRVLGTNPEMAPGDVKRLKQ GLANLK
MES17           MPKMKTHSSLKGRFKITGTGKVMRYKAYKNHLLSHKSKRAKRVLGTNPEMAPGDVKRLKQ GLANLK
MES108          MPKMKTHSSLKGRFKITGTGKVMRYKAYKNHLLSHKSKRAKRVLGTNPEMAPGDVKRLKQ GLANLK
CCI_25          MPKMKTHSSLKGRFKITGTGKVMRYKAYKNHLLSHKSKRAKRVLGTNPEMAPGDVKRLKQ GLANLK
J               MPKMKTHSSLKGRFKITGTGKVMRYKAYKNHLLSHKSKRAKRVLGTNPEMAPGDVKRLKQ GLANLK
YUPP_8          MPKMKTHSSLKGRFKITGTGKVMRYKAYKNHLLSHKSKRAKRVLGTNPEMAPGDVKRLKQ GLANLK
E681            MPKMKTHSSLKGRFKITGTGKVMRYKAYKNHLLSHKSKRAKRVLGTNPEMAPGDVKRLKQ GLANLK
YC0136          MPKMKTHSSLKGRFKITGTGKVMRYKAYKNHLLSHKSKRAKRVLGTNPEMAPGDVKRLKQ GLANLK
P3              MPKMKTHSSLKGRFKITGTGKVMRYKAYKNHLLSHKSKRAKRVLGTNPEMAPGDVKRLKQ GLANLK
MES972          MPKMKTHSSLKGRFKITGTGKVMRYKAYKNHLLSHKSKRAKRVLGTNPEMAPGDVRRLKQ GLANLK
                ********************** *****:**************************:**** ******
```

```
Cluster 1       MPKMKTHSSLKGRFKITGTGKVMRYKAYKNHLLSHKSKRAKRVLGTNPEMAPGDVKRLKQGLANLK
Cluster 2,3,4   MPKMKTHSSLKGRFKITGTGKVTRYKAYRNHLLSHKSKRAKRVLGTNPEMAPGDVRRLKQGLANLK
```

Figure S12. Multiple alignment of ribosomal protein L35. The sequences were obtained from the strains belonging to Cluster 1 (yellow), Cluster 2 (gray), Cluster 3 (green), and Cluster 4 (cyan). The consensus sequences of each Cluster are displayed in the box below the alignment. Cluster-specific amino acid substitutions are highlighted using the same colors of the Clusters. The MES972 strain is underlined as it has an R at position 56, which is present in Clusters 1, 2, and 3, but is replaced by K in the other strains belonging to Cluster 1.
